# Mastering Complexity toward High‐Performance Multi‐Principal Element Alloy‐Based Films and Coatings: A Review on Microstructural Regulation and Property Optimization

**DOI:** 10.1002/advs.202514258

**Published:** 2026-03-23

**Authors:** Jiaming Cao, Jiayi Sun, Heqing Li, Zhiqiang Wu, Xianghai An

**Affiliations:** ^1^ School of Aerospace Mechanical and Mechatronic Engineering and Sydney Nano Institute (Sydney Nano) The University of Sydney Sydney NSW 2006 Australia; ^2^ Sanya Research Institute of Hunan University of Science and Technology Sanya Hainan 572025 China

**Keywords:** microstructure regulation, multi‐principal element alloy, nanostructured coatings and thin films, strength/hardness and plasticity, thermal stability, wear and corrosion resistance

## Abstract

Multi‐principal element alloy (MPEA)‐based films and coatings are redefining the boundaries of materials science, offering an unprecedented synergy of mechanical robustness, thermal stability, and chemical resilience. Their performance arises from intrinsic chemical complexity, such as high configurational entropy, lattice distortion, sluggish diffusion, and synergistic elemental interactions, enabling the formation of refined microstructures and positioning them as ideal candidates for high‐performance applications in extreme environments. This review provides a comprehensive exploration of microstructure regulation strategies for MPEA‐based films and coatings, with a focus on the interplay between elemental composition, nanoscale architecture, heterostructures, and interfacial engineering in tailoring their properties and functions. A systematic analysis of their mechanical properties, alongside corrosion tolerance, wear and erosion resistance, and thermal stability is presented, while the fundamental mechanisms governing these exceptional performances are examined in depth. Additionally, their emerging and expanding applications in aerospace, biomedical, and extreme‐environment technologies are highlighted, and chart their future, emphasizing the integration of multi‐scale modeling, high‐throughput synthesis, and machine learning‐driven materials design to achieve predictive microstructure control and properties optimization. Finally, the challenges and opportunities that will shape the next era of high‐performing MPEA‐based films and coatings research are outlined, pushing the limits of what these extraordinary materials can achieve.

## Graphical abstract

This review provides a comprehensive exploration of microstructure regulation strategies for multi‐principal element alloy‐based films and coatings, with a focus on the interplay between elemental composition, nanoscale architecture, heterostructures and interfacial engineering in tailoring their properties and functions. A systematic analysis of their mechanical properties, corrosion tolerance, wear and erosion resistance, and thermal stability is presented.

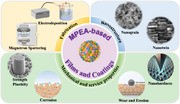

## 1 Introduction

High‐performance metallic materials are the backbone of modern engineering, underpinning critical advancements in transportation, aerospace, energy, mining, and advanced manufacturing. As industries push the boundaries of performance and durability, the demand for metals that achieve exceptional strength and ductility/toughness has never been greater. Traditional alloys, such as titanium alloys,^[^ ^1^ ^]^ magnesium alloys,^[^ ^2^ ^]^ aluminum alloys,^[^ ^3^ ^]^ and steels,^[^ ^4^ ^]^ are typically based on one or two primary elements, with minor alloying additions to fine‐tune their microstructures and properties. The key principle in conventional alloy design is minimizing intermetallic compound formation as that these intrinsically brittle phases can compromise mechanical performance. However, this approach inherently limits compositional flexibility, solid solution strengthening effects, and overall material properties.

In 2004, Yeh et al.^[^ ^5^ ^]^ and Cantor et al.^[^ ^6^ ^]^ introduced a groundbreaking paradigm shift: multi‐principal element alloys (MPEAs). Unlike traditional alloys, MPEAs break free from compositional constraints by incorporating multiple principal elements in equiatomic or near‐equiatomic ratios, dramatically increasing the configurational entropy. This unique strategy stabilizes solid solution phases while suppressing brittle intermetallic formation, unleashing an innovative spectrum of cutting‐edge metallic alloys. Defined by four core characteristics including high configurational entropy, lattice distortion, sluggish diffusion, and the “cocktail” effect,^[^ ^7^ ^]^ MPEAs exhibit exceptional mechanical properties,^[^ ^8^ ^]^ extraordinary corrosion resistance,^[^ ^9^ ^]^ excellent wear and erosion performance,^[^ ^10^ ^]^ and outstanding thermal stability.^[^ ^11^ ^]^ With their unparalleled combination of attributes, MPEAs are poised to redefine the future of next‐generation structural and functional materials, furnishing transformative solutions to the most pressing challenges in materials science.^[^ ^12^, ^13^ ^]^

With the burgeoning development of MPEAs, their applications in thin films and coatings have emerged as a rapidly growing research frontier. MPEA‐based films and coatings, typically ranging from tens of nanometers to several micrometers in thickness, harness the unique properties of MPEA while offering additional advantages, such as enhanced surface performance and adaptability to extreme environments. Remarkably, these films and coatings not only match the exceptional properties of their bulk counterparts but, in some cases, even surpass them. For instance, Liao et al.^[^ ^14^ ^]^ demonstrated this potential by fabricating nanocrystalline CoCrFeNiAl_0.3_ films with a highly preferential orientation via magnetron sputtering, achieving hardness approximately four times greater than the bulk alloy. Similarly, Li et al.^[^ ^15^ ^]^ introduced N atoms during sputtering to react with strong nitride‐binding elements (such as Ti, Nb, and Cr), inducing the formation of (TiNbCr)N nanocrystals embedded in an amorphous matrix and Cu/Ni segregation. This resulting nanocomposite (CuNiTiNbCr)N_X_ film exhibited superior hardness, toughness, and wear resistance, making it ideal for extreme wear environments. Beyond mechanical robustness, MPEA‐based films and coatings also delivered outstanding functional properties, opening new possibilities in corrosion resistance, biocompatibility, and radiation tolerance. Ma et al.^[^ ^16^ ^]^ tailored the sputtering parameters of ZrNbTiCrCu alloy films to achieve a BCC phase with Cu‐rich domains, significantly enhancing their corrosion resistance and biocompatibility. Ge et al.^[^ ^17^ ^]^ developed amorphous TiZrHfTaW films using direct current (DC) magnetron sputtering at room temperature and introduced a “migration‐blocking” strategy, effectively improving radiation resistance for advanced nuclear applications. Zoita et al.^[^ ^18^ ^]^ further expanded the design space by employing a hybrid magnetron sputtering technique to fabricate metallic and nitride coatings, optimizing Al and N content to tailor the microstructure and properties of (TiCrAl_X_NbY)N_Y_ coatings. These studies underscore the immense promise of MPEA films and coatings across diverse fields. In particular, refractory MPEA films and coatings, characterized by their simple crystal structures, excellent thermal stability, and superior mechanical and physical properties, have recently gained traction, offering transformative solutions for next‐generation protective and functional coatings.

The development of MPEA films and coatings has been propelled by advances in cutting‐edge processing techniques, including magnetron sputtering,^[^ ^19^ ^]^ laser cladding,^[^ ^20^ ^]^ plasma spraying,^[^ ^21^ ^]^ and electrochemical deposition.^[^ ^22^ ^]^ These techniques leverage the unique multi‐element nature of MPEAs to accurately manipulate elemental composition,^[^ ^23^, ^24^ ^]^ phase structure,^[^ ^25^ ^]^ and processing parameters,^[^ ^24^ ^]^ enabling tailored properties for diverse applications. Among these techniques, magnetron sputtering stands out due to its superior compositional control, high uniformity, and high‐density film formation, making it an ideal choice for creating high‐performance films. On the other hand, electrodeposition offers a simple, cost‐effective, and scalable approach that can coat complex geometries while allowing microstructural modification through fine‐tuned deposition parameters. However, despite its promise, electrodeposition remains in an exploratory stage for MPEA films and coating. Critical challenges such as optimizing process parameters, unveiling ion deposition mechanisms, and refining electrolyte formulation, still require significant research breakthroughs to unlock its full potential.^[^ ^26^, ^27^, ^28^ ^]^

The microstructure of MPEA‐based films and coatings plays a decisive role in shaping their mechanical, thermal, and functional properties.^[^ ^29^ ^]^ Key microstructural characteristics, such as grain size,^[^ ^30^ ^]^ phase composition,^[^ ^31^ ^]^ dislocation density,^[^ ^32^ ^]^ and nanoscale architecture,^[^ ^33^, ^34^ ^]^ have a profound influence on material performance. Researchers have devised a range of strategies to subtly tailor these structural attributes, including elemental selection, processing parameters optimization, and post‐deposition treatments. The choice of principal elements and their atomic ratios dictate the phase stability and mechanical behavior of MPEA films and coatings. For instance, incorporating elements with varying atomic radii can prominently enhance lattice distortion and solid solution strengthening.^[^ ^35^, ^36^ ^]^ Deposition conditions like sputtering power, substrate bias, current density, deposition temperature, and cooling rate govern phase evolution and grain morphology,^[^ ^37^, ^38^ ^]^ allowing for the judicious control of nanocrystalline, amorphous, or multiphase structures with superior mechanical and functional features. Furthermore, post‐processing techniques such as thermal annealing, hot isostatic pressing, and ion irradiation have been employed to refine microstructure, relieve residual stresses, and heal defects. By precisely tuning the internal structure and eliminating weaknesses, post‐processing elevates the intrinsic capabilities of MPEA films and coatings, transforming them into game‐changers in next‐generation materials engineering.^[^ ^39^, ^40^, ^41^, ^42^, ^43^ ^]^

MPEA‐based films and coatings represent a bold step toward the future of material innovation. Yet, this remarkable progress marks only the beginning. By harnessing advances in microstructural engineering, computational design, and process innovation, these materials hold tremendous potential to redefine performance standards across the most demanding industrial sectors. This review presents a comprehensive examination of recent breakthroughs in microstructure regulation and property optimization of MPEA films and coatings. We critically assess phase evolution pathways, strengthening strategies, and the profound impact of microstructural modifications on mechanical performance, wear and erosion resistance, corrosion tolerance, and thermal stability. Additionally, we explore emerging strategies in process optimization, state‐of‐the‐art characterization techniques, and theoretical computational modeling, highlighting their role in unlocking the full potential of these cutting‐edge materials. By bridging experimental research with theoretical insights, this review outlines a forward‐looking vision for the development of next‐generation high‐performance films and coatings with unparalleled multifunction, driving transformative innovations and accelerating advancements across a wide range of cutting‐edge technologies.

## 2 Microstructure Regulation

The microstructure of MPEA films and coatings is the foundation of their exceptional mechanical, thermal, and functional properties. Unraveling its evolution is not just important; it is the gateway to tailoring performance, pushing the boundaries of material design, and unlocking groundbreaking applications.

### 2.1 Crystal Structures and Phase Evolution

Compared to those produced through the conventional metallurgical processes, the microstructures of films and coatings typically exhibit distinctive characteristics owing to the highly non‐equilibrium nature of their fabrication techniques. In this context, the elemental composition and processing parameters are two fundamental drivers that govern the microstructure evolution, directly influencing phase formation, lattice distortion, and mechanical performance of MPEA‐based films and coatings. Even slight variations in composition or processing conditions can lead to dramatic shifts in crystal structure, unleashing novel properties and expanding their functional potentials.

**Figure 1:**
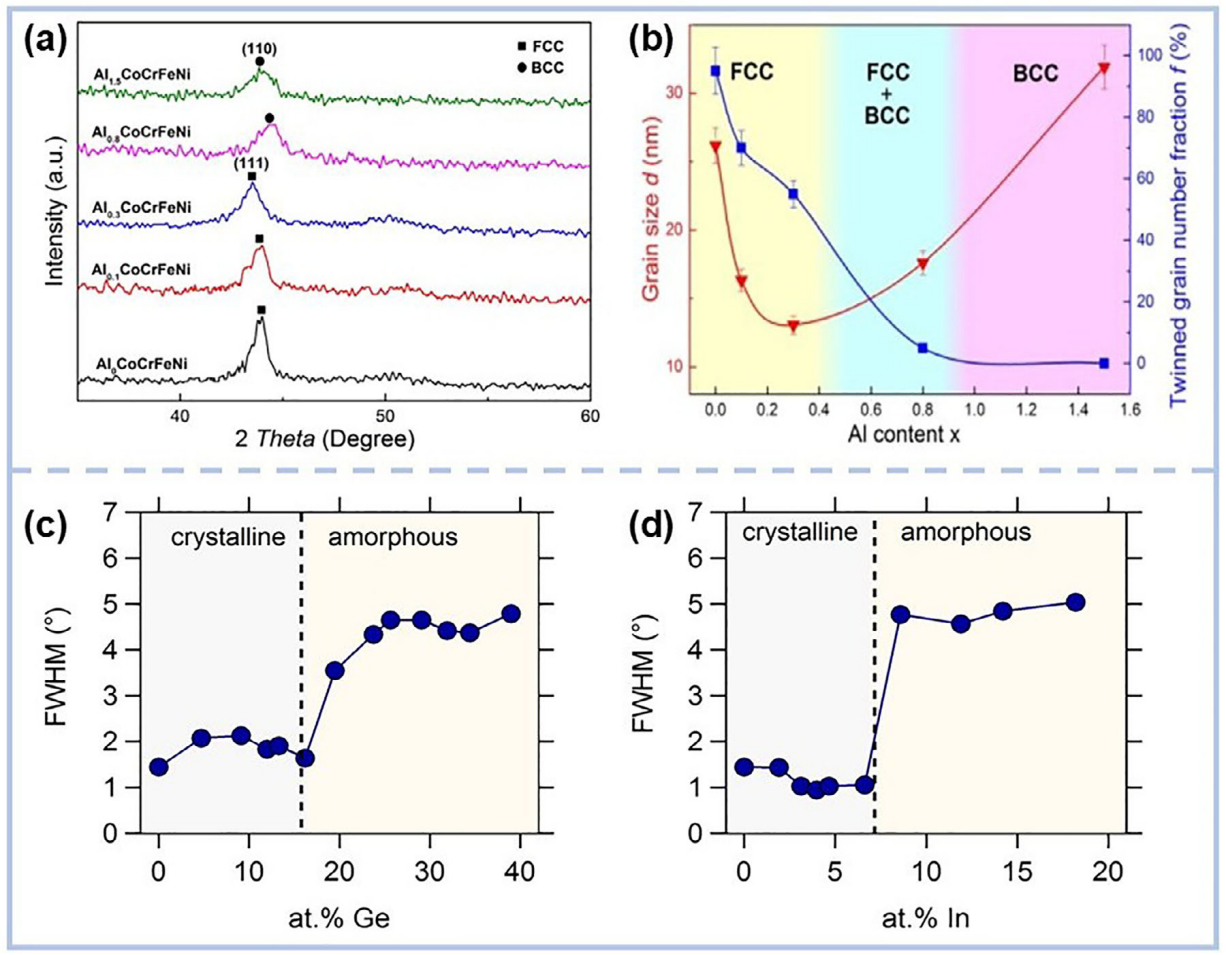
a) X‐ray diffraction (XRD) patterns of the Al_x_CoCrFeNi films; b) The grain size and the twinned grain number fraction of the Al_x_CoCrFeNi films as a function of the Al content^[^ ^44^ ^]^ Copyright 2017, Elsevier. Full width at half maximum (FWHM) of the main diffraction peaks of c) Ge_x_‐and d) In_x_‐CoCrCuFeNi films.^[^ ^46^ ^]^ Copyright 2016, Elsevier.

A striking example comes from Feng et al.,^[^ ^44^ ^]^ who investigated the effect of Al content on the phase structure of CoCrFeNi MPEA films via DC magnetron sputtering. As illustrated in **Figure** **1**a,b, increasing Al concentrations triggered a structural shift from a face‐centered cubic (FCC) to a body‐centered cubic (BCC) lattice. This transition, marked by a diffraction peak shifted toward lower angles, indicated an expansion in lattice parameters, which was a direct consequence of Al‐induced atomic rearrangements. Similarly, Braeckman et al.^[^ ^45^, ^46^ ^]^ systematically examined the effects of Nb, Ge, and In on the microstructure of CoCrCuFeNi MPEA films which prepared by magnetron sputtering. Their findings revealed that a conspicuous evolution: the initial single‐phase FCC structure morphed into an amorphous matrix interspersed with nanocrystals (Figure 1c,d). This transformation, driven by severe lattice distortion due to significant atomic radii variations, highlighted the delicate balance between crystallinity and disorder in MPEA systems.

Beyond metallic elements, reactive sputtering enables the incorporation of non‐metallic elements such as carbon, oxygen, and nitrogen, offering additional pathways for tuning phase evolution. Carbon, for example, can be introduced via co‐sputtering with a graphite target or through reactive magnetron sputtering using methane, acetylene, or other reactive gases. Fritze et al.^[^ ^47^ ^]^ demonstrated that carbon doping during CrNbTaTiW MPEA film deposition using magnetron sputtering altered the phase equilibrium, shifting the structure from BCC to FCC while simultaneously boosting hardness and elastic modulus. Oxygen and Nitrogen exhibited similar transformative effects. The oxygen incorporation into AlCoCrCu_0.5_NiFe MPEA films promoted the formation of a hexagonal close‐packed (HCP) phase,^[^ ^48^ ^]^ while nitrogen doping in the TiZrHfNiCuCo MPEA films induced crystallization, transitioning the material from an amorphous cluster into a dense FCC phase.^[^ ^49^ ^]^

Interestingly, the influence of nitrogen varies significantly across different MPEA systems. In alloys rich in strong nitride‐forming elements (e.g., Al, Cr, Ti, V, Zr, Nb, Mo, Hf, Ta), the original MPEA‐based film tends to be amorphous, while nitrogen incorporation tends to enhance crystallization, stabilizing an FCC phase such as AlCrMnMoNiZr,^[^ ^50^ ^]^ AlCrMoNiTi,^[^ ^51^ ^]^ and AlTiZrTaHf.^[^ ^52^ ^]^ However, in systems dominated by weak nitride‐forming elements (e.g., Co, Fe, Ni, Mn, Cu), nitrogen often disrupts the original crystal lattice, leading to amorphization. For instance, the original AlCoCrCuFeNi and AlCoCrCuFeNiMn films typically exhibited FCC or a BCC + FCC mixed solid solution structure, while as the nitrogen flow rate increased, the phase structure tended to become amorphous.^[^ ^53^, ^54^ ^]^ This contrasting behavior stems from the interplay between metal‐nitrogen bonding strength and system mixing enthalpy. Strong Me─N bonds drive structural ordering, whereas excessive interstitial nitrogen promotes lattice distortion, reducing the Gibbs free energy and favoring an amorphous state.^[^ ^54^ ^]^ Despite these insights, the precise atomic‐scale mechanisms governing nitrogen‐induced amorphization remain unclear, highlighting an important frontier for future research. Expanding on thermodynamic principles, Li et al.^[^ ^15^ ^]^ designed a (CuNiTiNbCr)N_X_ nanocomposite film with a tailored phase structure by leveraging the strong nitrogen affinity of Ti, Nb, and Cr. During deposition, nitrogen preferentially reacted and selectively bonded with these elements, forming a (TiNbCr)N crystalline phase with an FCC structure embedded within a Cu‐ and Ni‐rich amorphous matrix. This transformation yielded a highly refined nanocomposite architecture. Therefore, by carefully tailoring elemental composition, we can fine‐tune the microstructure and phase evolution of MPEA‐based films, driving the emergence of novel micro/nanoscale architecture and optimizing properties for critical applications.

**Figure 2:**
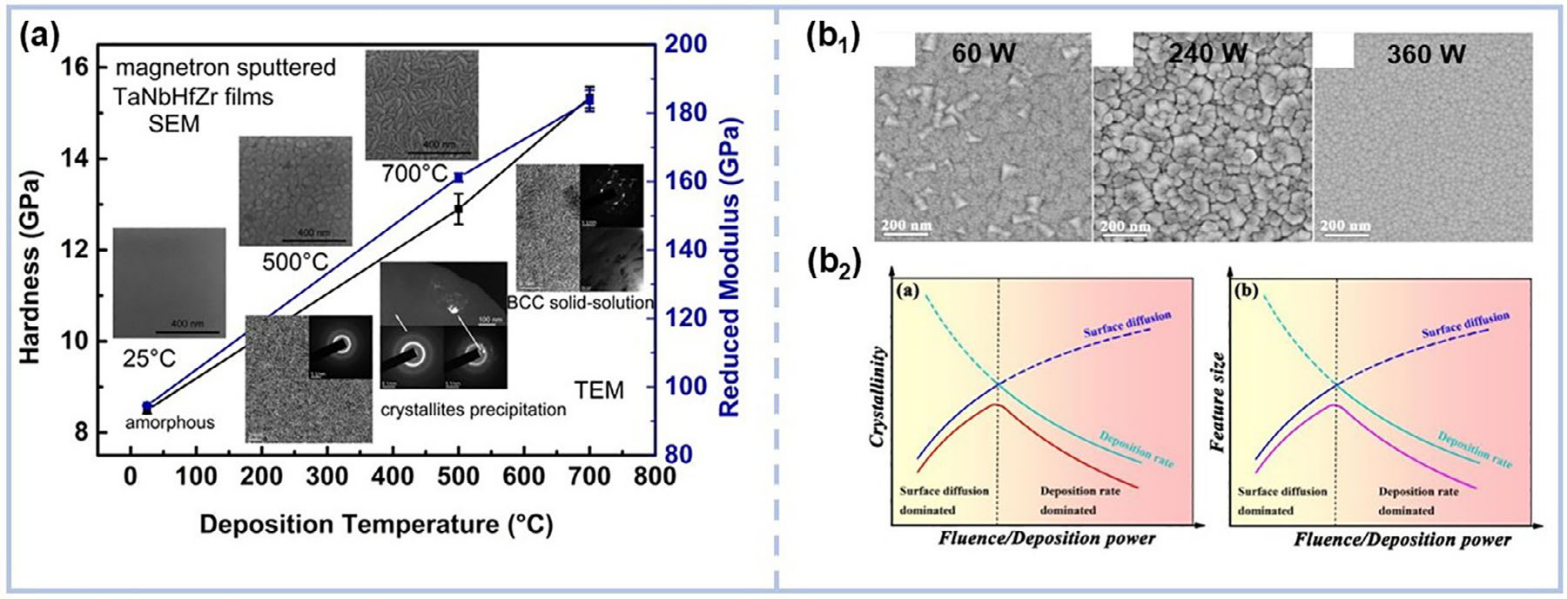
a) Evolution of microstructure, hardness, and reduced modulus of TaNbHfZr films with deposition temperatures.^[^ ^55^ ^]^ Copyright 2019, Elsevier. b_1_) Scanning Electron Microscope (SEM) images of Co‐Ni‐V films at various power conditions; b_2_) Schematically illustration of the variants in crystallinity and feature size with increasing fluence/deposition power, corresponding to the diffusion‐deposition competition.^[^ ^57^ ^]^ Copyright 2021, Elsevier.

The microstructure of MPEA‐based films and coatings is highly sensitive to deposition conditions, with substate temperature and sputtering power playing pivotal roles in phase evolution and grain growth. By meticulous tuning these parameters, we can subtly control the transition between amorphous and fully crystalline phases, optimizing the material's properties for specific applications. For instance, Song et al.^[^ ^55^ ^]^ investigated the effect of substrate temperature in shaping the crystal structure of TaNbHfZr MPEA films, revealing a prominent phase transformation. As illustrated in **Figure** **2**a, increasing the substrate temperature induced a shift from an amorphous structure to a BCC phase. This transition occurred because higher temperature enhanced atomic diffusion on the substrate surface, which facilitated atomic rearrangement, elimination of void boundaries, and promoted grain growth.^[^ ^56^ ^]^ Similarly, Ma et al.^[^ ^16^ ^]^ demonstrated that optimizing sputtering parameters and current levels enabled the fabrication of dense ZrNbTiCrCu MPEA films with a BCC and Cu‐rich phase structure, significantly improving film integrity and uniformity.

The impact of sputtering power on phase composition and grain structure was further elucidated by Hu et al.,^[^ ^57^ ^]^ who examined the CoNiV film under varying power conditions (Figure 2b_1_). Their findings revealed a complex phase evolution: at low sputtering power, the film exhibited a nanocrystalline‐amorphous dual‐phase composite structure; with increasing power, the structure transitioned to fully nanocrystalline and eventually became fully amorphous at higher power levels. This transformation was governed by the interplay between atomic surface diffusion and deposition kinetics. At lower sputtering power, sputtered atoms possessed relatively limited kinetic energy, favoring surface diffusion and enabling orderly atomic rearrangement into nanocrystalline domains. As sputtering power increased, atoms gained higher kinetic energy, accelerating both diffusion and deposition rates. While enhanced diffusion encouraged crystallization, the excessively rapid deposition reduced the time available for atomic rearrangement, thus driving amorphization. This competition between diffusion and depositions was critical in dictating the final microstructure, as illustrated in Figure 2b_2_. The corresponding changes in grain size also reflected this trend, correlating with variations in lattice structure. Similar microstructural trends have been observed in MPEA‐based nitride films, where substrate temperature and sputtering power dictate the balance between crystallinity and amorphization.^[^ ^58^ ^]^ Understanding these intricate relationships allows for strategic control over film properties, enabling the design of MPEAs with tailored microstructures and enhanced functional performance.

**Figure 3:**
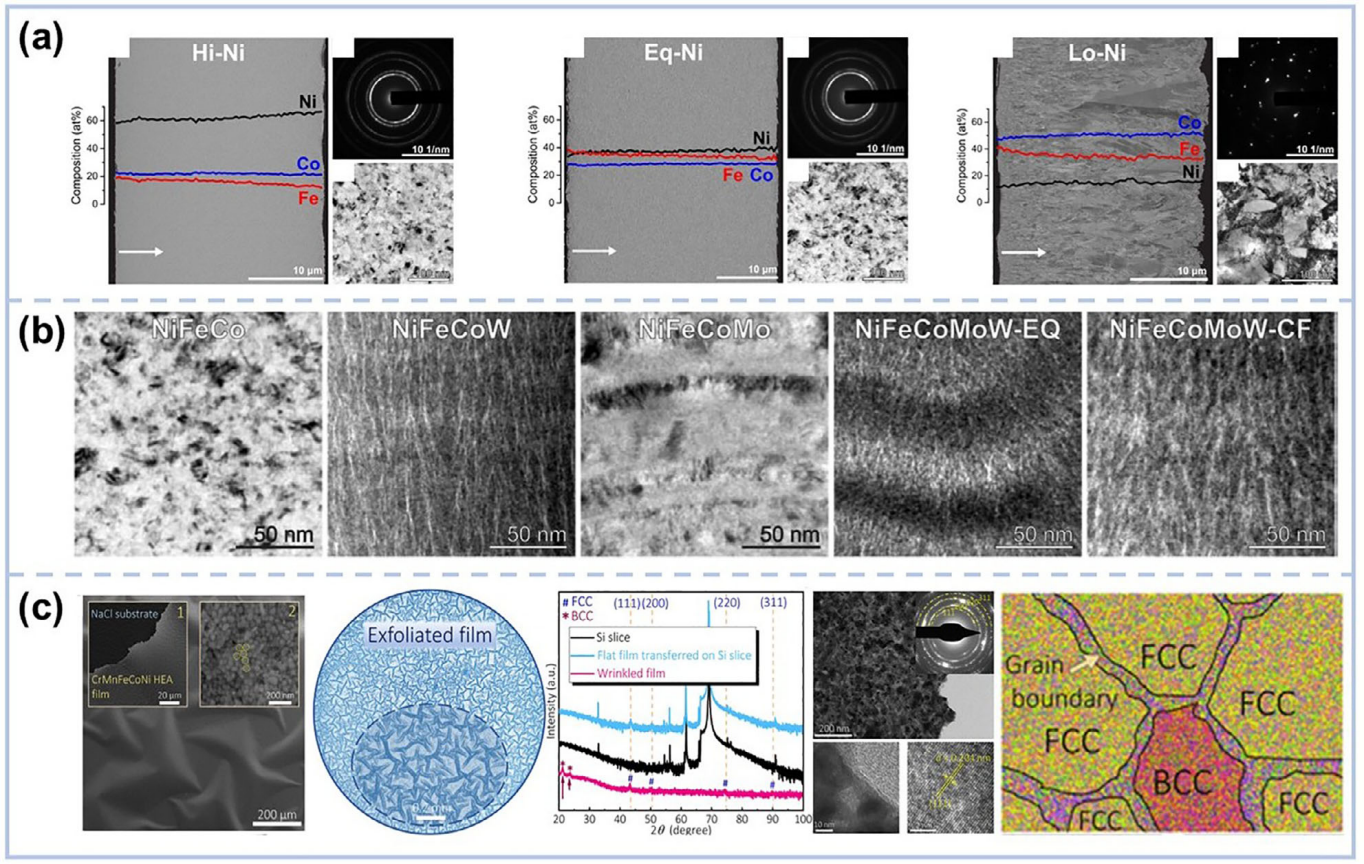
a) The microstructure of as‐deposited Hi‐Ni, Eq‐Ni, and Lo‐Ni NiFeCo electrodeposits^[^ ^27^ ^]^ Copyright 2022, Elsevier. b) Transmission Electron Microscope (TEM) images of the as‐deposited NiFeCo‐based MPEAs)^[^ ^26^ ^]^ Copyright 2023, Elsevier. c) Characterizations of the CrMnFeCoNi film on the NaCl substrate via SEM, optical microscope, XRD, and TEM, as well as a schematic illustration of its phase structure).^[^ ^64^ ^]^ Copyright 2023, American Chemical Society.

Electrochemical deposition has emerged as a cost‐effective and scalable method for fabricating MPEA‐based films and coatings,^[^ ^37^, ^59^ ^]^ allowing judicious manipulation of phase composition through adjustments in current density, applied potential, and plating solution conditions.^[^ ^60^ ^]^ This makes it a versatile and practical technique for tailoring MPEA‐based film and coating properties. A comparative summary of the phase structures achieved via electrodeposition is provided in **Table** **1**, underscoring how fine‐tuned deposition parameters drive significant microstructural transformations. For instance, using DC electrodeposition, Huo et al.^[^ ^61^ ^]^ demonstrated that varying deposition current densities yielded CoNiFe films with an FCC structure and a preferred <111> orientation. Haché et al.^[^ ^27^ ^]^ showcased the transformative effect of Ni content in electrodeposited NiFeCo coatings, revealing a dramatic shift in crystal structure: high‐Ni alloys exhibited a nanocrystalline FCC phase, whereas low‐Ni content led to a coarse‐grained BCC structure (**Figure** **3**a). However, in NiFeCr coatings, high oxygen contamination often resulted in amorphous structures, limiting their applicability in high‐performance environments. To improve deposit quality, Haché et al.^[^ ^26^ ^]^ incorporated alloying elements such as W, Mo, P, and Re,^[^ ^62^, ^63^ ^]^ successfully producing high‐quality NiFeCo‐W, NiFeCo‐Mo, and NiFeCo‐MoW coatings. These coatings, deposited from aqueous solution, displayed distinct amorphous banded structures. Notably, NiFeCo‐W and NiFeCo‐MoW coatings formed elongated nanoglass boundaries along the growth direction (Figure 3b), while NiFeCo‐Mo exhibited alternating nanocrystalline and amorphous bands. These unique structures endowed the films with exceptional mechanical and functional properties, enhancing their technological promise.

**Table 1:** Phase structures of MPEA‐based films and coatings prepared by electrodeposition.

Composition	Type of electrodeposition	Phase structure	Electrolyte based/additive	Ref.
BiFeCoNiMn	DC electrodeposition	Amorphous + FCC (After annealing)	DMF‐CH_3_CN	[59]
MgMnFeCoNiGd	DC electrodeposition	Amorphous	DMF‐CH_3_CN	[71]
AlCrCuFeMnNi	DC electrodeposition	Amorphous + FCC (After annealing)	DMF‐CH_3_CN	[22]
AlFeCoNiCu	DC electrodeposition	BCC + FCC	GO	[72]
MnCrFeCoNi	DC electrodeposition	BCC	GO	[73]
CuFeNiCoCr	DC electrodeposition	BCC + FCC	GO	[74]
AlCrFeCoNiCu	DC electrodeposition	BCC + FCC	GO	[75]
FeCoNiCrMn	DC electrodeposition	FCC	——	[76]
CoCrFeMnNi	pulse electrodeposition	FCC	——	[70, 77]
NiFeCo NiFeCr	DC electrodeposition	FCC/BCC FCC	—— ——	[27]
CoNiFe	DC electrodeposition	FCC	——	[61]
NiFeCo‐W NiFeCo‐Mo NiFeCo‐MoW	DC electrodeposition	——	——	[78]
FeCoNiCrW	DC electrodeposition	FCC	——	[79]
FeCoNi	DC electrodeposition	FCC	DMF‐CH_3_CN	[80]

Expanding beyond conventional DC electroplating, pulse current electrodeposition introduces an additional layer of control by cyclically and rapidly alternating the current or potential, enabling more refined tailoring of film composition and microstructure.^[^ ^65^ ^]^ Beyond current and voltage, three key parameters including pulse‐on time, peak current density, and pulse‐off time, play a crucial role in dictating the final quality of MPEA‐based films and coatings. Each adjustment in these parameters influences the elemental distribution, phase formation, and grain structure of the deposits. Liu et al.^[^ ^66^ ^]^ leveraged this approach to fabricate nanocrystalline NiCr coatings on 30CrNiMo steel. By elaborately adjusting current density, they achieved varying Cr contents and coating thicknesses. XRD analysis revealed that both Ni and NiCr coatings exhibited nanoscale grain structures, with Cr incorporation subtly shifting peak positions of the NiCr coating due to lattice strain, which consisted exclusively of the FCC phase. Taking pulse electrodeposition further, Xu et al.^[^ ^67^ ^]^ developed amorphous/nanocrystalline Ni‐P/Ni‐Mo coatings, capitalizing on the unique advantages of each structure. XRD analysis unveiled as distinctive diffraction pattern: a broadened Ni (111) peak in Ni‐Mo coating, indicative of nanocrystalline domains embedded within an amorphous matrix.^[^ ^68^, ^69^ ^]^ In contrast, the Ni‐P single‐layer coating exhibited a characteristic “bun‐shaped” Ni (111) peak near 45°, confirming a fully amorphous structure. To enhance control over phase formation, ZrO_2_ nanoparticles were introduced, refining the microstructures and reducing crystallite size. Compared to Ni‐P, Ni‐Mo, and Ni‐P/Ni‐Mo dual‐phase coatings, the Ni‐P/Ni‐Mo‐ZrO_2_ dual‐phase coating displayed a denser and more uniform morphology, demonstrating the effectiveness of nanoparticle‐assisted phase tuning.

Apart from electrochemical deposition, pulse laser deposition (PLD) offers another avenue for structural engineering. Ling et al.^[^ ^64^ ^]^ harnessed the expansion and contraction of a NaCl substrate along with weak van der Waals interactions at the interface to create self‐supporting CrMnFeCoNi MPEA films via PLD. Upon transfer to an adhesive substrate, these films developed strain‐induced wrinkles, triggering a flexomagnetic effect characterized by a localized, non‐uniform strain gradient that induced a partial structural phase transition from FCC to BCC (Figure 3c). This phase transformation prominently boosted the room‐temperature saturation magnetization by an astonishing factor of ten compared to the flat, strain‐free MPEA‐based film, showcasing how strain engineering can drive dramatic functional enhancements.

Yoosefan et al.^[^ ^70^ ^]^ further refined pulse electrodeposition by systematically investigating the effects of frequency and duty cycle on the microstructure of the CoCrFeMnNi MPEA films. At a fixed frequency of 5000 Hz, reducing the duty cycle significantly increased Fe content from 24.15% to 30.76%. When applying a frequency of 2500 Hz, the Fe content in the deposits increased from 24.75% to 27.38%. Additionally, Cr content also responded dynamically, increasing from 7.23% to 9.94% at 2500 Hz and from 8.01% to 11.80% at 5000 Hz as the duty cycle rose. Interestingly, frequency adjustments affected grain size in a complex, non‐linear manner: at a 50% duty cycle, higher frequency led to smaller grains, while at a 60% duty cycle, the opposite trend occurred. XRD analysis confirmed a single‐phase FCC solid solution, reinforcing the delicacy of pulse electrodeposition in achieving tailored microstructures.

### 2.2 Nanostructure Engineering

#### 2.2.1 Nanocrystalline Structure

MPEA‐based films and coatings with nanocrystalline microstructures have attracted widespread attention due to their exceptional mechanical strength, hardness, and wear resistance.^[^ ^81^ ^]^ However, the high density of grain boundaries (GBs) inherent in nanocrystalline materials introduces a critical limitation: the elevated free energy of the system accelerates grain growth, undermining thermal stability.^[^ ^82^, ^83^ ^]^ Furthermore, their limited capacity to sustain dislocation accumulation weakens strain‐hardening ability, thereby restricting the deformability. As such, the targeted judicious modification of nanocrystalline structures in MPEA films and coatings remains a frontier in materials research.

**Figure 4:**
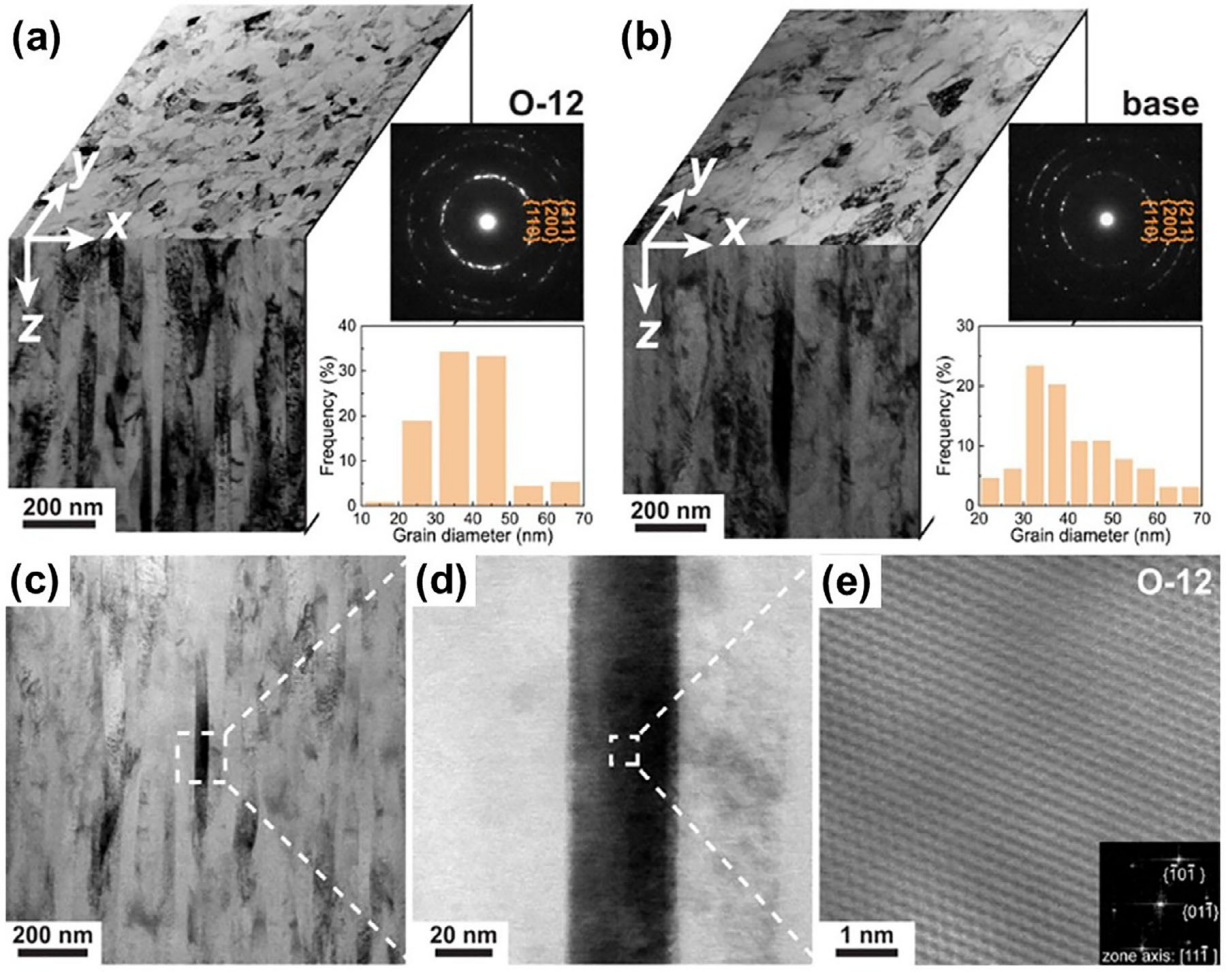
Microstructures of a,c–e) the (TiNbZr)_86_O_12_C_1_N_1_ (O‐12) alloy, and b) the equiatomic TiNbZr alloy.^[^ ^85^ ^]^ Copyright 2022, Springer Nature.

Within this context, advanced deposition techniques have opened new pathways to overcome these challenges through microstructural tailoring at the atomic scale. Among them, magnetron sputtering stands out due to its inherently non‐equilibrium nature, facilitating the formation of columnar nanocrystalline structures along the film growth direction. Leveraging the unique chemical disorder and lattice distortion intrinsic to MPEAs, researchers have begun pushing the limits of this technique.^[^ ^84^ ^]^ For instance, Liu et al.^[^ ^85^ ^]^ pioneered the development of a novel class of massive interstitial solid solution alloys by incorporating high concentrations of interstitial elements into a TiNbZr MPEA system. As displayed in **Figure** **4**, the introduction of up to 12 at.% oxygen along with trace amounts of carbon and nitrogen resulted in the formation of a TiNbZr‐O‐C‐N alloy that retained a nanocrystalline structure without forming secondary oxides. This remarkable outcome was achieved by exploiting the solubility of oxygen in the highly distorted lattice and using carbon and nitrogen atoms to stabilize GBs through segregation. These interstitials not only elevated strength but also endowed the films with enhanced plastic formability, offering an exceptional plasticity‐hardness combination. Similar N‐supersaturation strategies have been applied to FeMnCoCr MPEA films, enabling fine‐tuning of grain size, defect distribution, phase stability, and mechanical performance.^[^ ^86^, ^87^ ^]^

Leveraging the thermodynamic principles, Liu et al.^[^ ^19^ ^]^ engineered a (TiZrNbHf)_98_Ni_2_ nanograined MPEA film via magnetron sputtering. The strategic addition of Ni, with its large negative mixing enthalpy and distinct electronegativity, promoted compositional fluctuations and chemical ordering within the grains. Moreover, Ni atoms preferentially segregated to GBs, effectively lowering their energy and increasing the hardening effect of GBs. Pushing this concept even further, Duan et al.^[^ ^8^ ^]^ introduced a sophisticated order–disorder core–shell nanostructure in a NiCoFeAlTiB alloy which fabricated by conventional arc melting, wherein multielement co‐segregation formed a thin disordered FCC nanolayer around an ordered grain interior. This core–shell architecture achieved via controlled segregation and lattice coherency, delivered extraordinary thermal stability and mechanical performance, illustrating the untapped potential of nanoscale architecture engineering.

While sputtering‐based approaches offer precise atomic‐scale control, the quest for nanocrystalline MPEA films via electrodeposition, a cost‐effective and scalable technique, presents additional hurdles. Chief among them is microscale compositional inhomogeneity, which often stems from kinetic limitations during ion transport and nucleation.^[^ ^88^ ^]^ Nevertheless, recent breakthroughs are beginning to surmount these barriers. Péter et al.^[^ ^89^ ^]^ demonstrated the successful fabrication of CoFeNiZn nanocrystalline films using pulse electrodeposition. By carefully modulating current parameters, they achieved uniform elemental distribution along the film thickness and a columnar grain structure with an average size of ≈12 nm. This refinement led to significant enhancements in both hardness and elastic modulus, establishing the promise of pulse‐modulated deposition techniques. Expanding on this approach, Song et al.^[^ ^80^ ^]^ pioneered a hybrid technique, namely friction‐assisted electrodeposition, to selectively grow FeCoNi coatings on GCr15 steel substrate. This method mechanically disrupted the multiple layers during deposition, refining the grain structure and accelerating nucleation. Selected area electron diffraction (SAED) pattern revealed concentric diffraction rings, indicative of a dense nanocrystalline structure composed of randomly oriented ultrafine grains.^[^ ^90^, ^91^ ^]^

Despite these encouraging advancements, the field still grapples with the unpredictability of nanocrystal formation. The complex interplay between elemental composition and deposition conditions makes it difficult to achieve precise tuning of grain size and phase distribution. Developing a predictive framework, powered by data‐driven modeling and machine learning, for nanocrystalline MPEA‐based films would be a game‐changer, paving the way for materials that combine exceptional strength, ductility, and thermal resilience, and ultimately redefining the future of high‐performance coatings.

#### 2.2.2 Nanotwinned Structure

Nanotwinned structures, characterized by densely packed twin boundaries (TBs) within nanometer‐scale twin lamellae, represent a cutting‐edge microstructural design that elevates the mechanical, thermal, and physical performance of materials. These TBs, mirror‐like interfaces within the crystal lattice, act as effective barriers to dislocation motion, significantly enhancing strength. Unlike high‐angle GBs, however, TBs retain crystallographic continuity, allowing for improved plastic deformation and strain hardening. This unique combination results in materials that are not only exceptionally strong, but also incredibly ductile and thermally stable, making nanotwinned architectures highly desirable for high‐performance coatings, microelectronics, and structural applications under extreme conditions.

A seminal study in the field of nanotwinned materials was conducted by Lu et al.,^[^ ^92^ ^]^ who introduced high‐density nanotwins into ultrafine‐grained copper using an electrodeposition technique. Their work revealed a striking relationship between twin thickness and mechanical performance. As twin thickness decreased, both ductility and strain‐hardening capability consistently improved. However, maximum strength was achieved at a critical twin thickness of 15 nm. Beyond this threshold, increasing twin thickness led to a reduction in strength due to decreased barrier effects on dislocation motion. Conversely, when the twin thickness fell below 15 nm, strength dropped sharply, likely due to the transition from a Hall‐Petch type of strengthening to a dislocation‐nucleation‐controlled softening mechanism at ultra‐fine spacings. This pioneering insight established a fundamental principle in nanotwin engineering that there exists an optimal twin spacing which balances strength and ductility. Since then, this principle has guided numerous studies exploring the potential of nanotwinned microstructures in high‐performance coatings and films, particularly in the context of MPEAs.

**Figure 5:**
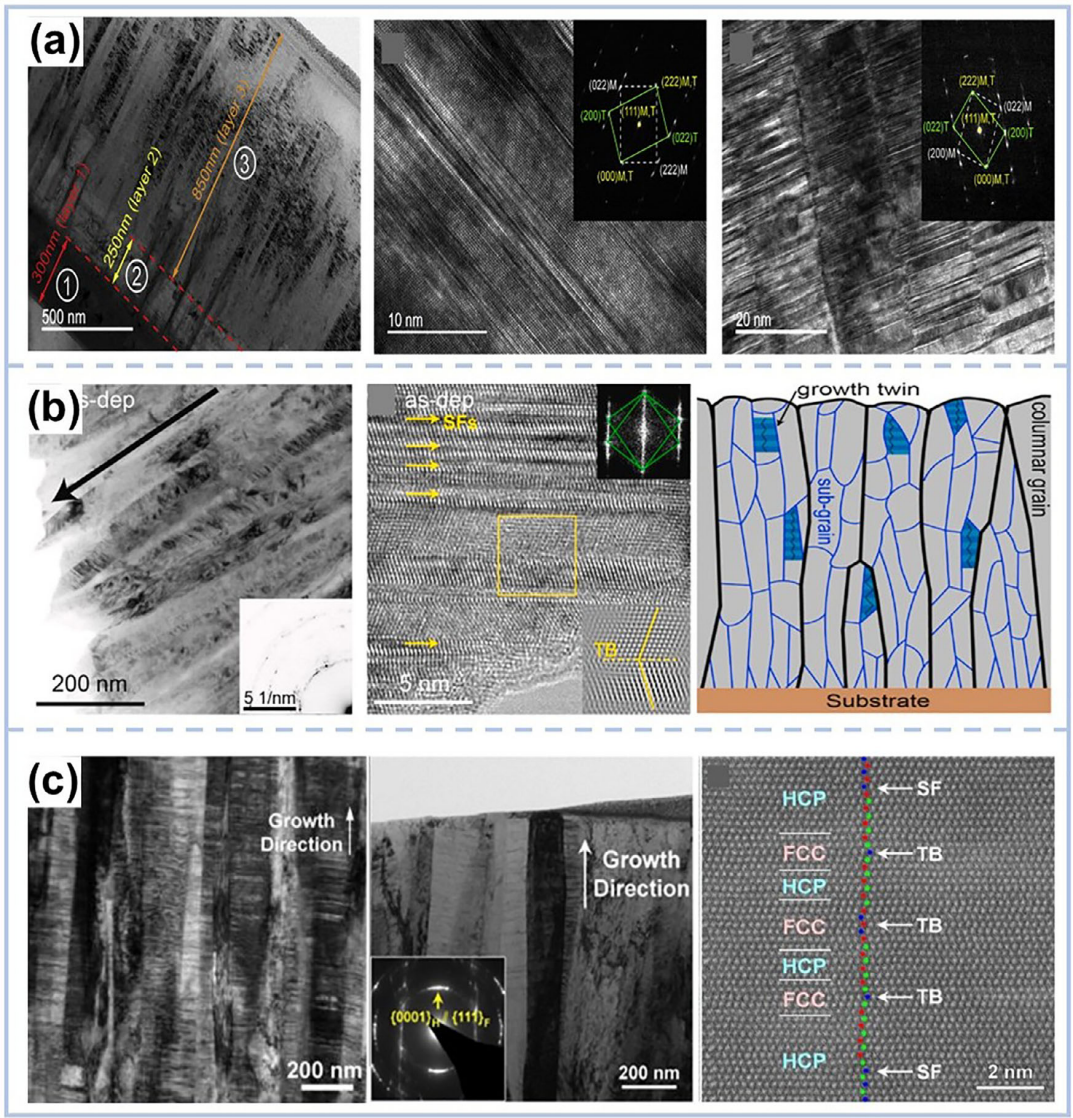
a) TEM images of the as‐deposited CoCrFeMnNi film with the thickness of 1400 nm^[^ ^93^ ^]^ Copyright 2022, Elsevier. b) TEM characterizations of FeCoNiCrCu films, and the schematic illustration of its microstructure)^[^ ^94^ ^]^ Copyright 2021, Elsevier. c) Microstructures of the as‐deposited CrCoNi film including columnar grains and high‐density defects.^[^ ^86^, ^95^ ^]^ Copyright 2021, Elsevier, Copyright 2018, AIP.

A particularly illustrative study by Wang et al.^[^ ^93^ ^]^ successfully fabricated CoCrFeMnNi MPEA coatings using radio frequency magnetron sputtering, revealing a three‐layered structure as deposition thickness reached 1400 nm (**Figure** **5**a): i) an amorphous base layer with nanocrystalline fragments, ii) a middle layer featuring single‐crystal nanograins with twin spacings of 2.2–5.6 nm, and iii) a top columnar layer with twin spacings of 1.2–2.4 nm. These structural transitions critically influenced mechanical behavior. Coatings with larger twin spacing (2.2–5.6 nm) demonstrated high hardness and moderate fatigue resistance, while smaller twin spacings (1.2–2.4 nm) led to exceptional fatigue resistance alongside high strength. These effects stemmed from a shift in deformation mechanisms, where dislocation blocking dominated at twin spacings greater than 2 nm, whereas twinning‐mediated plasticity prevailed below 2 nm, consistent with Lu's twin‐thickness theory.

Several subsequent studies further validated the functional role of nanotwins in MPEA‐based coatings. Zhang et al.^[^ ^94^ ^]^ prepared metastable single‐phase FCC FeCoNiCrCu films via magnetron sputtering and observed a high density of growth nanotwins and stacking faults, as shown in Figure 5b. Similarly, Wang et al.^[^ ^96^ ^]^ produced CoCrFeMnNi films via radio frequency magnetron sputtering with high nanotwin densities on (100)‐Si substrates. Fatigue lifetime testing confirmed that these nanotwins significantly prolonged fatigue life via detwinning mechanisms under cyclic loading.^[^ ^93^ ^]^ Atomistic simulations by Zhou et al.^[^ ^97^ ^]^ reinforced these findings by demonstrating that nanotwins enhanced crack resistance through detwinning and crack‐closure processes along the fatigue path. Further evidence comes from Chen et al.,^[^ ^86^, ^95^, ^98^, ^99^ ^]^ who fabricated CrCoNi films via magnetron sputtering composed of alternating FCC and HCP nanolayers with high densities of stacking faults and nanotwins (twin spacing: ≈50–150 nm), as illustrated in Figure 5c. These films exhibited excellent mechanical properties and a novel bidirectional phase transformation, underscoring the robustness of twin‐enabled strengthening and unparalleled microscopic plastic mechanisms in MPEA systems.

A more complex picture of the structure evolution was revealed by Feng et al.,^[^ ^44^ ^]^ who systematically studied the influence of nanotwins in Al_X_CoCrFeNi (x = 0, 0.1, 0.3) films fabricated via magnetron sputtering. They observed that, as the Al content increased, the microstructure transitioned from an FCC to a dual‐phase region, followed by a significant increase in the fraction of single‐phase BCC structures. Concurrently, the proportion of nanotwins decreased noticeably. The study found that these extremely fine nanotwins impart two contrasting effects. On one hand, they tend to soften the material, while on the other, they enhance its strain‐rate sensitivity, a combination that is unusual yet highly advantageous for dynamic mechanical applications. This dual behavior highlights the nuanced role of nanotwins and their interplay with grain size and phase structure in tuning mechanical response. Expanding on this work, Feng et al.^[^ ^100^ ^]^ developed nanolaminated Cu/FeCoCrNi films with nanotwinned structures (based on earlier efforts^[^ ^101^ ^]^) via DC magnetron sputtering. By incorporating a soft and ductile Cu layer, the films achieved enhanced strength and ductility through the activation of microcrack initiation and coordinated deformation, highlighting another nanotwin‐inspired design strategy for MPEA‐based films.

Beyond mechanical performance, nanotwinned structures have shown promise in functional applications. Wang et al.^[^ ^102^ ^]^ introduced a defect engineering strategy by doping FeCoNiCuMoB films with B interstitial atoms via pulsed DC reactive magnetron sputtering. This reduced the system's stacking fault energy (SFE) and enhanced GB stability, promoting the formation of fivefold twin structures,^[^ ^103^, ^104^ ^]^ which markedly improved both catalytic performance and structural stability.

Together, these studies paint a vivid picture of the transformative potential of nanotwinned architectures in MPEA‐based coatings. Far beyond simply reinforcing the material, nanotwins introduce a sophisticated mechanism of strengthening that simultaneously preserves ductility, improves fatigue life, enhances strain‐rate sensitivity, and even unlocks new functional properties such as catalytic activity. What emerges is a new paradigm in microstructural design: one where TBs act not just as barriers, but as dynamic interfaces, mediating plasticity, guiding crack propagation, and enhancing structural resilience under extreme conditions. This level of tunability is particularly crucial in the context of MPEAs, whose inherent chemical complexity and structural diversity offer fertile ground for synergistic interactions between twins, grains, and phases.^[^ ^105^, ^106^, ^107^ ^]^ As fabrication techniques continue to mature, the future of nanotwinned MPEA‐based coatings appears not only promising, but essential. These structures are no longer just mechanical reinforcements, while they are active, intelligent design elements, redefining what is possible in advanced materials for extreme environments.

### 2.3 Heterostructure

The integration of heterostructure design into metallic systems has emerged as a powerful strategy to overcome the longstanding trade‐off between strength and ductility. Recently, this approach has gained significant traction in the development of MPEA‐based films and coatings. These materials with inherent compositional complexity are proving to be ideal platforms for engineering hierarchical heterogeneous microstructures, enabling simultaneous enhancement of mechanical performance and functional properties.^[^ ^12^, ^108^ ^]^ Researchers have leveraged this potential by exploring a diverse array of heterostructural configurations encompassing localized chemical composition fluctuations,^[^ ^109^ ^]^ medium/short‐range ordered structures,^[^ ^110^, ^111^ ^]^ gradient grain structures,^[^ ^112^ ^]^ bimodal structures,^[^ ^113^ ^]^ lamellar structures,^[^ ^114^ ^]^ gradient nanotwin structures,^[^ ^115^ ^]^ and multiphase structures,^[^ ^116^ ^]^ among others. The essence of this strategy lies in harnessing spatial heterogeneity to stimulate mechanisms such as dislocation trapping, strain partitioning, and energy dissipation, thereby achieving synergistic property enhancements.

A compelling example of this can be found in the work by Zhang et al.,^[^ ^94^ ^]^ who synthesized FeCoNiCrCu films via magnetron sputtering. Upon moderate heat treatment at 773 K, these films exhibited remarkable plasticity and strain hardening while maintaining high strength. The underlying mechanism was traced to the emergence of deformation twins, triggered by nanoscale chemical fluctuations that were absent in the as deposited and lightly annealed states. Atomistic simulations supported this finding, confirming that such nanoscale fluctuations could promote twinning and, in turn, strengthen–ductility synergy.

Nature, as it turns out, has long exploited structural gradients to optimize material function. From the hierarchical lamellae in seashells to the porosity gradient in tree trunks and the bone density variation in skeletal systems, gradients are used to distribute stress, absorb energy, and resist failure. Inspired by these natural architectures, Cheng and colleagues developed nano‐twinned Cu with a controlled structural gradient by tuning electrolyte temperatures during electrodeposition.^[^ ^117^ ^]^ This gradient design enabled a progressive change in twin lamella thickness and grain size along the film's thickness. Remarkably, a larger gradient not only elevated the yield strength, reaching 481 MPa, but also improved strain hardening while maintaining good plasticity. The strongest region was no longer the limiting factor; the synergy among different zones enabled the whole material to perform better than its parts. Lin et al.^[^ ^118^ ^]^ successfully fabricated a dual‐gradient structure in Ni‐P alloy coatings by continuously varying the current density and additive concentration during the DC electrodeposition process. This dual‐gradient structure integrated both grain size and chemical composition gradients. By introducing this gradient structure, interfacial deformation was better accommodated, and crack initiation and propagation within the coating were effectively suppressed, achieving a desirable combination of high interfacial bonding strength and hardness for Ni‐P alloy coatings. Furthermore, by optimizing the gradient distribution within the coating, the bending strength of the gradient coating could be further enhanced.

**Figure 6:**
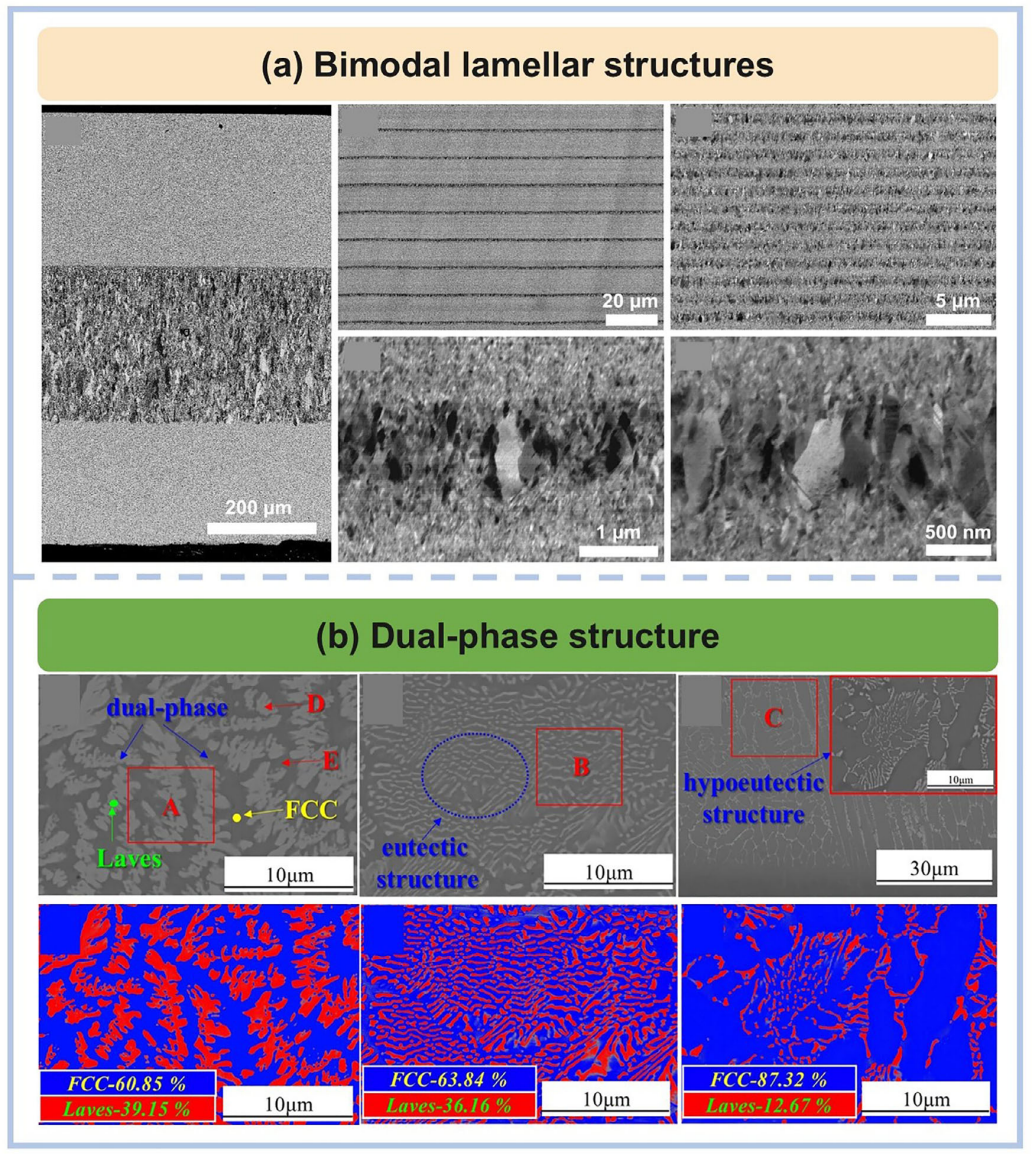
a) SEM and TEM images of NiCo multilayers, showing its bimodal lamellar structures^[^ ^119^ ^]^ Copyright 2020, Elsevier. b) SEM morphologies and dual‐phase maps of FeCoCrNi‐W coatings, taken from the top, middle, and bottom regions in the cross‐sectional plane.^[^ ^120^ ^]^ Copyright 2024, Elsevier.

Beyond gradients, the concept of bimodal structures has gained traction. These designs introduce alternating soft and hard domains or variations in grain size, allowing deformation to be localized and managed across different regions. Daly et al.,^[^ ^119^ ^]^ for instance, prepared NiCo coatings using pulsed electrodeposition that feature a bimodal lamellar architecture (**Figure** **6**a): hard nanocrystalline layers with a grain size of 20 nm alternated with softer ultrafine‐grained layers. This configuration allowed strain to be accommodated by the softer layers, while the harder layers provided strength, achieving an elegant balance of ductility and strength. Similar principles guided the design of eutectic MPEA‐based coatings. Guan et al.^[^ ^120^ ^]^ fabricated FeCoCrNi‐W coatings via laser cladding where the dual‐phase structure, consisting of an FCC phase and a hard Laves phase, was controlled through bidirectional diffusion of tungsten (Figure 6b). The Laves phase contributed to hardness and wear resistance, while the FCC matrix improved oxidation resistance and lubricity. Together, they provided superior performance under high‐temperature tribological conditions.

These advances collectively demonstrate the immense potential of heterostructure design in tuning the mechanical properties of MPEA‐based films and coatings. These growing insights have catalyzed a shift toward cross‐scale heterogeneous grain architectures as a next‐generation design strategy for MPEA‐based coatings. By precisely tuning magnetron sputtering or electrodeposition parameters, it is now possible to engineer microstructures that incorporate heterogeneity across multiple length scales, ranging from nanotwins and stacking faults to layered nanograins and phase boundaries.^[^ ^121^ ^]^ Such architectures capitalize on synergistic strengthening mechanisms: they combine heterogeneous deformation‐induced hardening with twin‐ and fault‐mediated plasticity, enabling materials to sustain high strength without sacrificing ductility.^[^ ^122^, ^123^ ^]^ Moreover, the complex internal interfaces inherent to these structures can activate additional deformation modes under mechanical loading, such as twinning, detwinning, and faulting, leading to enhanced strain‐hardening capacity and superior resistance to fatigue or failure.^[^ ^124^, ^125^ ^]^ This multiscale design paradigm represents a transformative approach to tailoring the mechanical performance of MPEA‐based films and coatings and offers a powerful pathway toward achieving the elusive combination of ultra‐high strength, exceptional ductility, and dynamic mechanical resilience. Yet, despite the promising results, the field remains in its infancy. Most studies to date have been exploratory, and there remains much to uncover about the mechanisms of deformation, interface behavior, and long‐term stability in these complex systems. Moreover, realizing the full promise of these structural heterogeneities will require deeper insight into the structure–property relationships and scalable strategies for industrial implementation.

### 2.4 Multilayer and Nanolaminate Structure

Leveraging the remarkable versatility of magnetron sputtering and electrochemical deposition, researchers have unlocked unprecedented control over the architecture of MPEA films, particularly in crafting multilayered and nanolaminated structures that harness interface phenomena to extraordinary effect. These techniques offer high precision in tuning layer thickness, composition, and interfacial characteristics, enabling the deliberate engineering of microstructures that transcend the limitations of conventional monolithic coatings. Such finely tailored architectures serve as powerful platforms for activating multiple strengthening and toughening mechanisms simultaneously. From interface‐mediated hardening and confined dislocation motion to strain accommodation via phase transformation, these structurally engineered MPEA‐based coatings embody a new generation of materials that are not only mechanically superior but also adaptable to extreme environments. In this context, multilayer and nanolaminate designs are no longer just structural enhancements, while they are strategic innovations, redefining how performance is built into materials from the nanoscale up.

Among the paradigm shift in structural engineering, dual‐phase heterostructures featuring FCC and BCC constituents have demonstrated remarkable synergistic effects. For instance, in CoCrNi/Ti multilayer films deposited on an M2 steel substrate using a DC magnetron sputtering,^[^ ^126^ ^]^ the CoCrNi layer at the top exhibited an FCC structure, while the bottom layer displayed a BCC + FCC structure. The resulting columnar grains at each interface facilitated the formation of stable shear bands during deformation, leading to significantly enhanced damage tolerance compared to monolithic counterparts. Extending this concept, Cao et al.^[^ ^127^, ^128^ ^]^ engineered CoCrNi/NbMoWTa (FCC/BCC) multilayers via DC magnetron sputtering, where sublayer thickness decisively influenced the interfacial phase stability. While ultra‐thin layers promoted amorphization, optimal thickness enabled the synergistic interplay of FCC and BCC phases, delivering a balanced combination of high strength and uniform plastic strain. Similarly, Lee et al.^[^ ^129^ ^]^ fabricated (AlCrNbSiTi)N/TiN multilayers via high‐frequency pulsed magnetron sputtering. As the number of alternating layers increased to 128, hardness rose steadily, peaking at 29.5 GPa without compromising its toughness and ductility, which can be attributed to interface strengthening, microstructural refinement, and the columnar growth mode enabled by the deposition technique.

**Figure 7:**
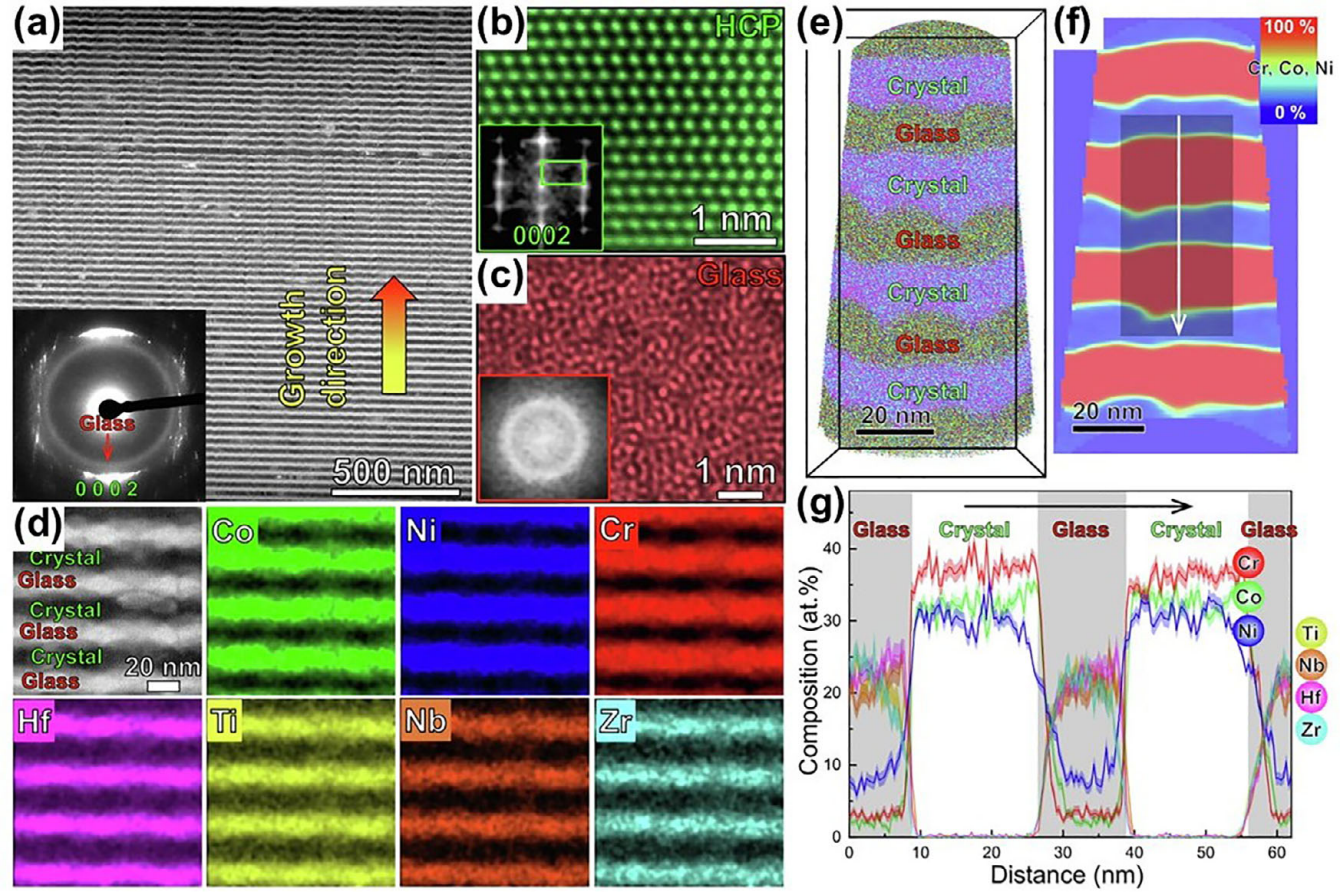
Microstructure of the CoCrNi/TiZrHfNb: a) High‐angle annular dark‐field (HAADF) images; b,c) Scanning TEM (STEM) images of CoCrNi and TiZrHfNb, showing HCP structure and amorphous structure, respectively; d) Energy dispersive spectroscopy (EDS) mappings; e) 3D reconstruction of atom probe tomography (APT); f) 2D distribution plot of Co, Cr, and Ni; g) 1D compositional profiles along the arrow in (f).^[^ ^130^ ^]^ Copyright 2021, Elsevier.

A particularly exciting frontier lies in crystalline–amorphous and amorphous–amorphous nanolaminates, where phase contrast and structural discontinuities offer unique avenues for tuning deformation behavior. In a sophisticated design, researchers combined crystalline Cr‐Ni‐Co layers with amorphous Zr‐Ti‐Nb‐Hf‐Ni‐Co, leveraging negative mixing enthalpy to induce a stress‐driven crystalline‐to‐amorphous transformation. This phase evolution, accompanied by dislocation absorption at the interfaces, enabled ultra‐high strength and uniform plastic deformation, demonstrating that heterointerfaces can function as both barriers and buffers under load. Wu et al.^[^ ^130^ ^]^ introduced the concept of “symbiotic MPEA films” with a CoCrNi/TiZrHfNb film structure via magnetron sputtering, where CoCrNi exhibited an HCP phase and TiZrHfNb showed an amorphous phase, as shown in **Figure** **7**a–g. During the sputtering process, dynamic element redistribution between the two phases stabilized the amorphous phase even at high temperatures. The resulting dual‐phase HCP/amorphous architecture delivered a superior strength–plasticity combination.

**Figure 8:**
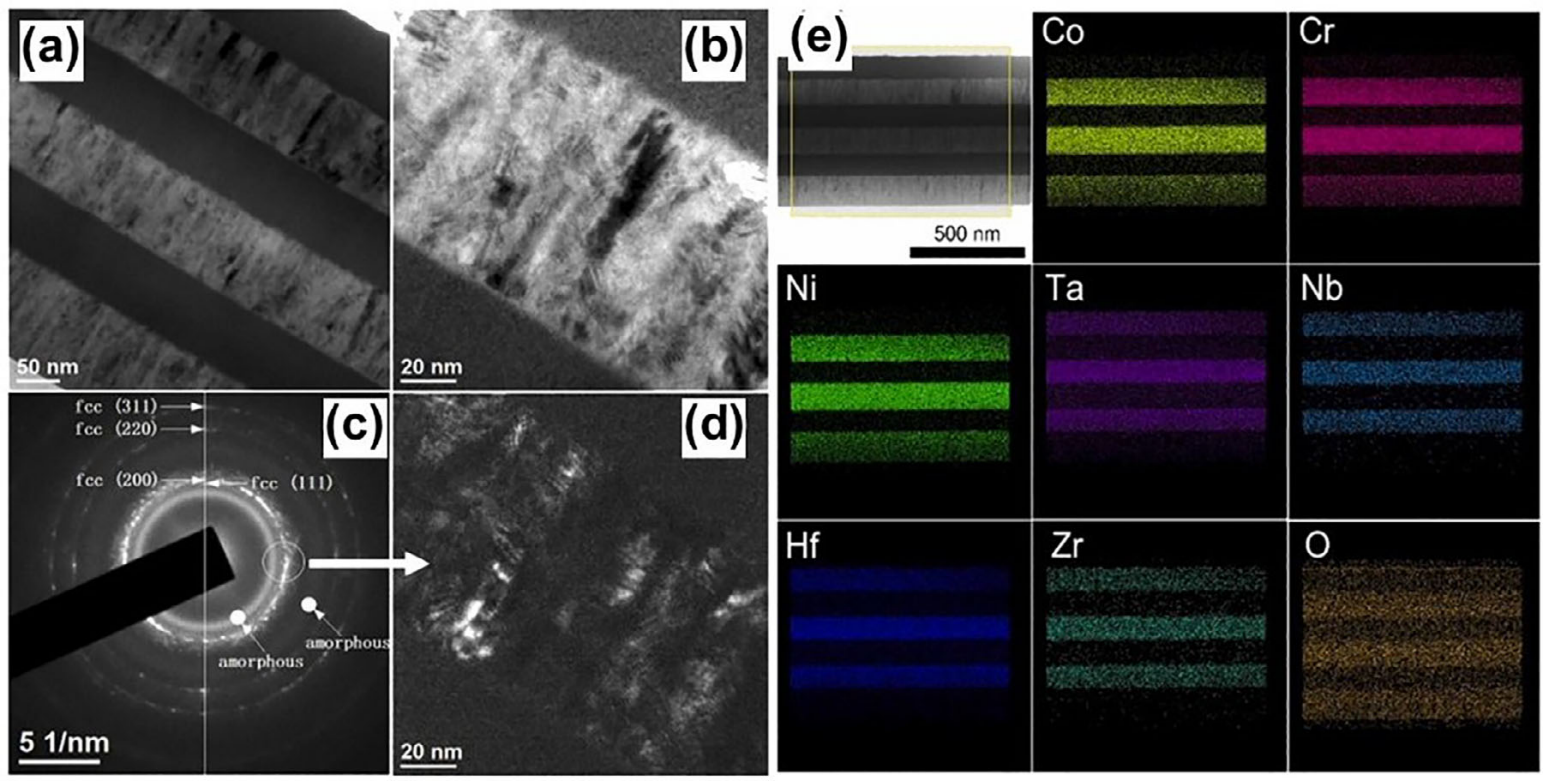
a,b) Morphologies of TaNbHfZr/CoCrNi multilayer coating; c) SAED image; d) Dark field image corresponding to (c); e) Elemental distribution by EDS mapping.^[^ ^131^ ^]^ Copyright 2022, Elsevier.

Further advancing this design logic, Song et al.^[^ ^131^ ^]^ employed the concept of high‐entropy metallic glass to fabricate TaNbHfZr/CoCrNi multilayer coatings through magnetron sputtering with an amorphous/FCC structure. As exhibited in **Figure** **8**, TEM characterization revealed flat, well‐bonded interfaces and uniformly distributed nanograins (≈20 nm) without compositional segregation. This architecture significantly reduced stress concentration^[^ ^132^ ^]^ and suppressed the growth of coarse columnar grains, improving the coating's resistance to crack propagation and enhancing its overall mechanical integrity.^[^ ^133^ ^]^ Another leap forward came from embedding ultrahigh‐density nanotwins within a metallic glass matrix by incorporating glass‐forming elements (B and Si) into a CrFeCoNi matrix. The nanotwinned–amorphous structure achieved a strength of 4.1 GPa, approaching the theoretical shear strength limit for metals, while maintaining toughness, illustrating the potent synergy between crystalline and amorphous domains.

**Figure 9:**
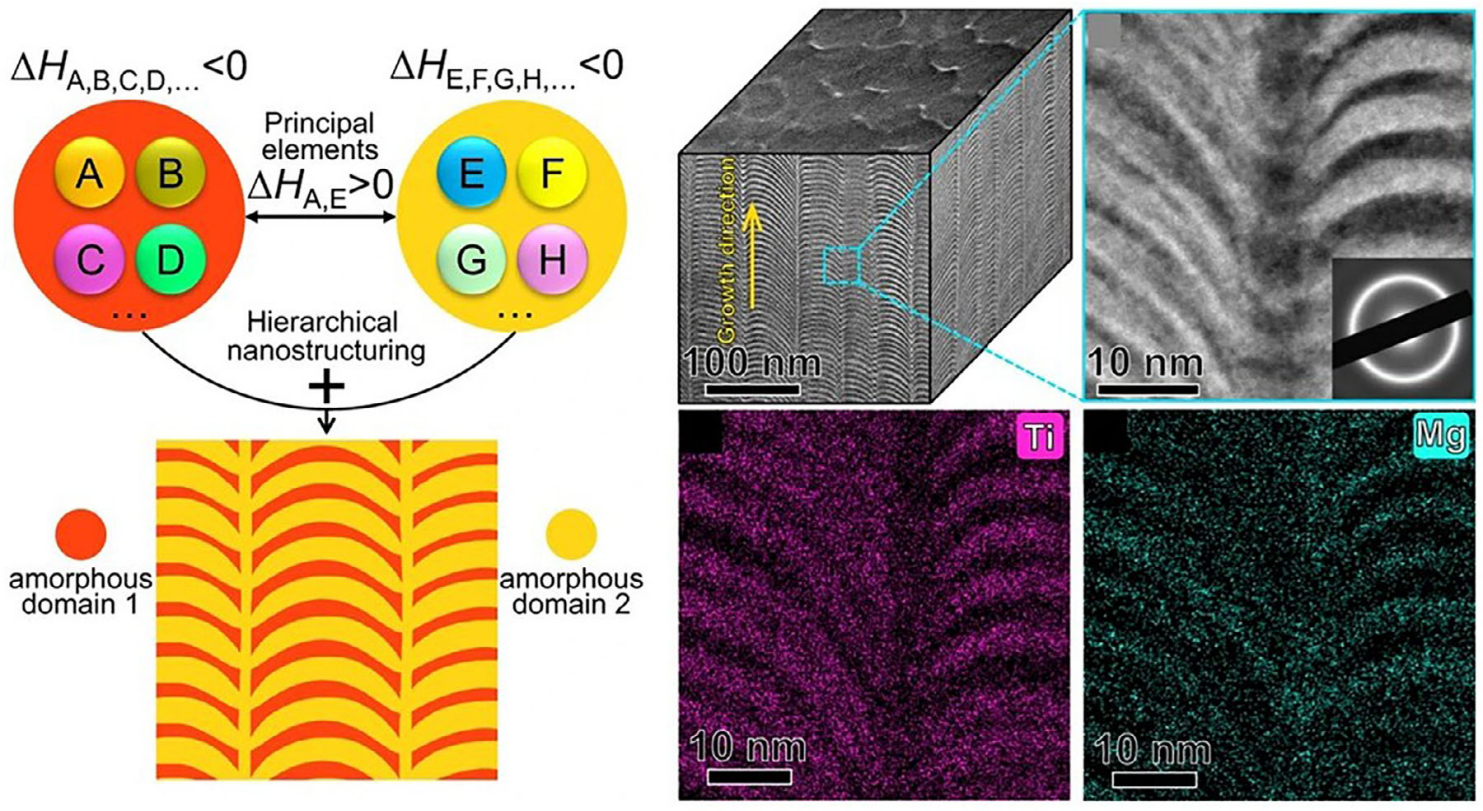
Enthalpy‐guided alloy design strategy for the hierarchically nanolaminated amorphous alloy.^[^ ^135^ ^]^ Copyright 2023, Springer Nature.

Pushing the boundaries even further, Cheng and colleagues developed an amorphous multilayer MPEA film consisting of alternating layers of (AlCrFeNi)N and TiN, fabricated by reactive radio‐frequency magnetron sputtering.^[^ ^134^ ^]^ Each layer, only 6 nm thick, allowed for gradual stress distribution during frictional contact and facilitated the formation of lubricating oxide products at the surface. As a result, the multilayer film exhibited wear rates 2.8 times lower than the single‐layer (AlCrFeNi)N and 8.4 times lower than pure TiN, clearly demonstrating the advantage of heterointerfaces in mechanical durability. Moreover, researchers constructed an amorphous–amorphous nanolaminate composed of alternating Ti‐Zr‐Nb‐Si‐XX/Mg‐Zn‐Ca‐YY layers with high compositional contrast via magnetron sputtering (**Figure** **9**).^[^ ^135^ ^]^ The pronounced mismatch in mixing enthalpy suppressed interdiffusion, stabilized sharp interfaces, and inhibited shear band formation. This architectural control facilitated homogeneous plastic flow, a rare yet highly desirable attribute in metallic glass systems.

Collectively, these multilayers and nanolaminate MPEA architectures represent a transformative shift in materials design, where performance is no longer dictated solely by composition, but strategically engineered through nanoscale structuring and interfacial control. Whether through phase contrast, enthalpy mismatch, or dual‐phase synergy, these heterostructured systems activate multiple, often complementary, deformation mechanisms that transcend the limitations of conventional monolithic films. By harnessing the full spectrum of interface phenomena such as dislocation blocking, phase transformation, strain delocalization, and stress‐driven amorphization, the deliberate construction of such nanoscale architectures stands poised to redefine the frontier of next‐generation coatings for high‐performance applications.

### 2.5 Interface Structure

The design of MPEA interfaces has emerged as a game‐changer in the quest for advanced energy storage solutions, particularly in sodium and lithium‐ion batteries. In these systems, controlling ion deposition and stripping at the interface is crucial for improving battery efficiency and longevity, and then design of robust, high‐performance interfaces is highly required. However, the synthesis of MPEA interfaces is far from straightforward. It demands complex, energy‐intensive fabrication techniques, often involving high‐energy ball milling, high‐temperature processing, or high‐pressure sintering. Moreover, the inherent reactivity and instability of sodium and lithium metals complicate the situation further, with conventional high‐temperature treatments frequently pushing the metals past their melting points. Meanwhile, achieving uniform interface coatings remains a significant challenge, especially when using mechanical alloying via high‐energy ball milling. Given these hurdles, developing stable and effective MPEA interface coatings on metal surfaces is critical for the future of high‐capacity, long‐life batteries.

**Figure 10:**
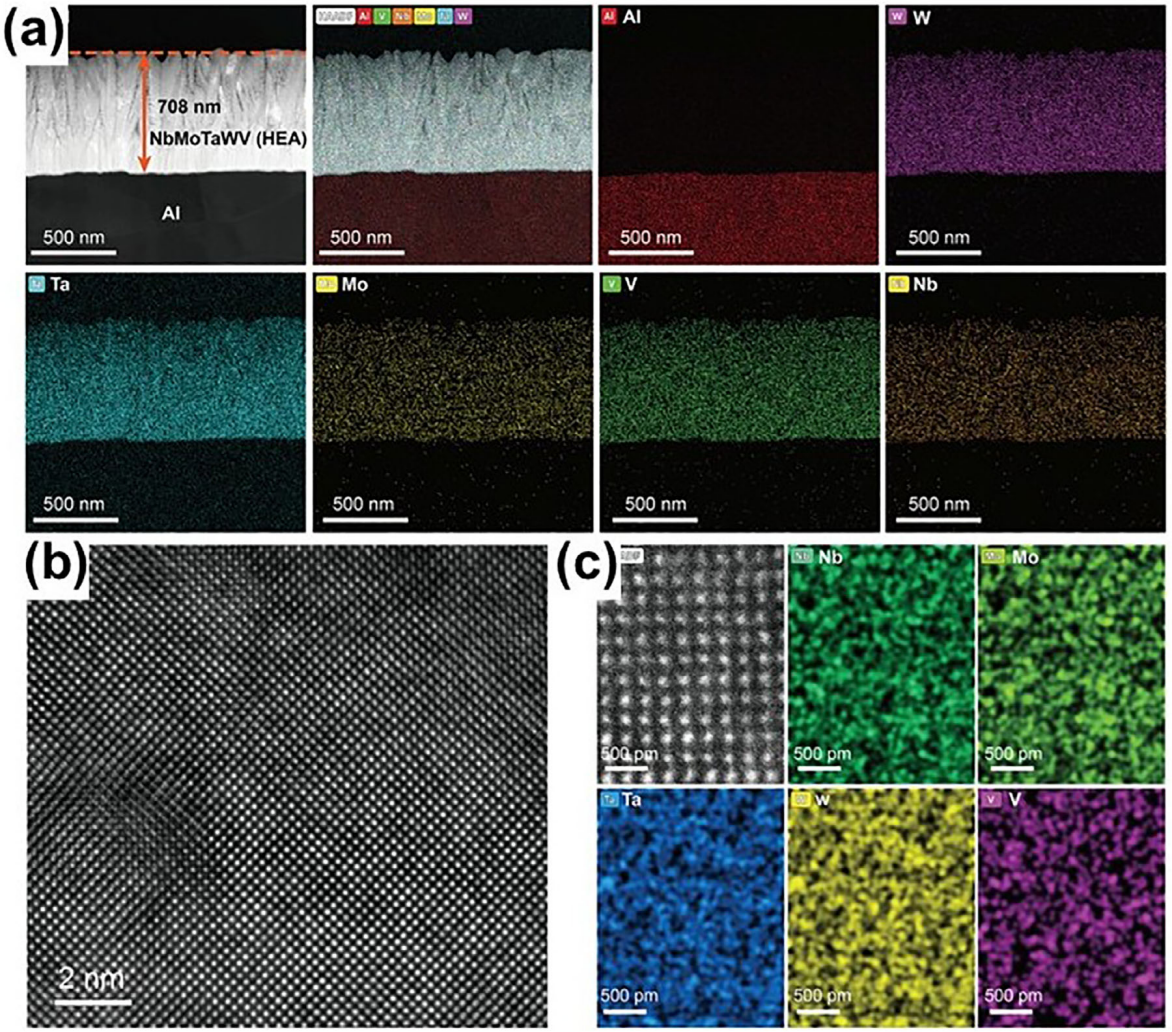
a) Cross‐sectional HAADF‐STEM image and the corresponding EDS mappings of the NbMoTaWV@Al; b,c) Atomic structure of the NbMoTaWV and the atomic‐scale EDS mappings.^[^ ^136^ ^]^ Copyright 2025, Wiley‐VCH.

In response to these challenges, Liu et al.^[^ ^136^ ^]^ capitalized on the unique advantages of MPEAs, such as their high‐entropy effects and the “cocktail” effect, to develop a thin NbMoTaWV alloy interface layer on a commercial aluminum foil current collector via magnetron sputtering. By ensuring uniform distribution of the elements (**Figure** **10**a–c), they created a highly active interface that promotes ion diffusion and regulates the uniform growth of sodium ions. First‐principles calculations confirmed that the NbMoTaWV alloy exhibited stronger binding energy with sodium than aluminum, facilitating more efficient sodium nucleation and improving overall ion transport. Similarly, Zheng et al.^[^ ^137^ ^]^ pioneered the creation of an amorphous MPEA interfacial phase on lithium metal using room‐temperature magnetron sputtering. Here, the combination of elements such as Al, Zn, Co, Ni, and Fe resulted in an interface rich in lithium‐philic sites that effectively controlled the flux distribution of lithium, ensuring consistent and uniform deposition. Further advancing the field, Xin et al.^[^ ^138^ ^]^ employed electrodeposition to fabricate a 3D ternary alloy layer on commercial zinc foil. Their Zn‐Sn‐Bi@Zn anode utilized the synergistic effects of Sn and Bi to solve critical interfacial issues in zinc anodes, significantly improving the electrochemical performance of aqueous zinc‐ion batteries.

These innovative studies underscore the great potential of MPEA interfaces in stabilizing sodium and lithium metal batteries, offering new strategies for enhancing their efficiency and lifetime. By leveraging the synergistic effects of multiple principal elements, these interfaces can facilitate uniform ion growth, improve stability, and significantly enhance electrochemical performance. As fabrication techniques evolve, MPEA interfaces are poised to overcome longstanding challenges and accelerate the development of high‐performance energy storage systems.

## 3 Mechanical, Corrosive, Wear and Erosive, and Thermal Properties

MPEA‐based films and coatings hold immense potential in redefining the limits of material performance, offering a remarkable suite of mechanical properties tailored for extreme‐service applications. By harnessing their inherent chemical and configurational complexity, lattice distortion, and sluggish diffusion mechanisms, MPEA‐based films and coatings have demonstrated exceptional promise in resisting mechanical degradation, surviving aggressive chemical environments, and maintaining structural integrity under high temperatures. These attributes are tunable through compositional design and processing methods that refine microstructure. This section explores the fundamental structure–property relationships, with a focus on recent advances that are pushing the boundaries of films and coating mechanical robustness and functionality, which is not only scientifically intriguing but also critical for unlocking their full potential as next‐generation protective and functional materials.

### 3.1 Mechanical Properties

Extensive research has demonstrated that MPEA‐based films and coatings possess exceptional mechanical properties, including high hardness and strength, excellent elastic modulus, remarkable deformability, and superior fatigue resistance. These outstanding characteristics are largely governed by their microstructural features, which, in turn, are heavily influenced by the fabrication technique. In particular, MPEA films and coatings prepared via magnetron sputtering or electrodeposition show a strong correlation between microstructure and mechanical performance, highlighting the importance of precise structural control in optimizing material properties.

#### 3.1.1 Micro/Nanohardness

The development of MPEA‐based films and coatings has opened exciting avenues for designing advanced materials with ultra‐high hardness and mechanical resilience, suited for demanding applications in microelectronics, aerospace, and protective coatings. The incorporation of multiple principal elements, solid solution strengthening, and tailored nanoscale architectures such as nanotwins, amorphous regions, and multilayer structures collectively endow MPEA films and coatings with outstanding mechanical characteristics.

Nanoindentation and microhardness testing are widely employed to assess the hardness and elastic modulus of these coatings, which reflect their resistance to deformation and structural integrity. A growing body of work has demonstrated that specific compositional and structural design strategies can dramatically enhance these properties. For instance, Liao et al.^[^ ^139^ ^]^ synthesized FCC‐structured Al_0.3_CoCrFeNi MPEA films using magnetron sputtering, exhibiting excellent hardness (≈11.09 GPa) and a remarkable yield strength of 1024 MPa in nanopillars, three times higher than those of their bulk counterparts. This enhancement was attributed to extensive GB hardening and nanocrystalline structures. Similarly, Li et al.^[^ ^140^ ^]^ produced dense FeAlCuCrCoMn MPEA films via DC magnetron sputtering. With increased deposition time, a single‐phase FCC solid solution developed, yielding a maximum hardness of 17.5 GPa and Young's modulus of 186 GPa. Chen et al.^[^ ^86^, ^95^, ^98^ ^]^ fabricated CrCoNi films composed of alternating FCC and HCP nanolayers with high densities of stacking faults and nanotwins, also via DC magnetron sputtering. These films exhibited a hardness of ≈10 GPa, which is about four times higher than that of as‐cast CrCoNi, due to the hierarchical heterogeneous architecture and nanoscale features.

**Figure 11:**
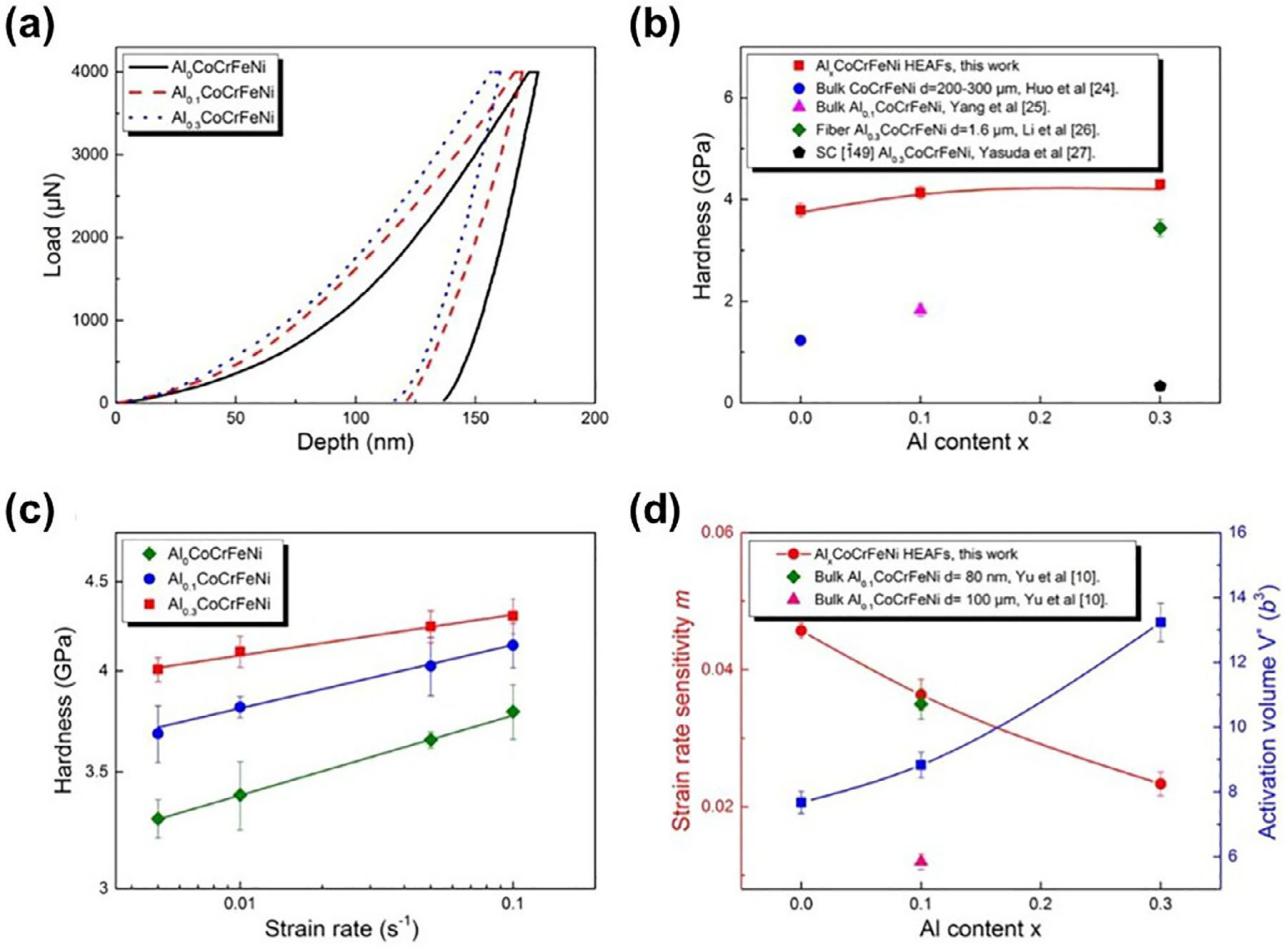
Nanoindentation results of Al_X_CoCrFeNi films with varying Al contents.^[^ ^44^ ^]^ Copyright 2017, Elsevier.

Furthermore, Feng et al.^[^ ^44^ ^]^ studied the role of nanotwins on the plastic deformation of Al_X_CoCrFeNi films using DC magnetron sputtering. As shown in **Figure** **11**, the hardness gradually increased with the addition of Al, reaching a maximum value of 4.3 GPa at x = 0.3, associated with a transition from FCC to dual‐phase and then to BCC‐dominated structures and decreased nanotwins density. Although these nanotwins slightly softened the material, they significantly enhanced its strain‐rate sensitivity. Cui et al.^[^ ^141^ ^]^ deposited BCC‐structured AlCoCrFeNi MPEA coating on 304 stainless steel substrates using laser metal deposition, incorporating a CoFe_2_Ni intermediate layer to enhance interfacial bonding. The AlCoCrFeNi layer reached an average hardness of 418 HV, while the intermediate layer measured 275 HV. This layered architecture eliminated crack formation and improved mechanical stability. Zhang et al.^[^ ^142^ ^]^ investigated NbTaW and Nb‐Ta‐W‐Hf films fabricated via multi‐target DC magnetron co‐sputtering. The NbTaW films exhibited a single‐phase BCC solid solution with a grain size of 16–30 nm, achieving hardness values of 25–29 GPa and an elastic modulus of 294–325 GPa. When Hf was introduced, the structure evolved from BCC into the fully amorphous phase, and the NbTaWHf films displayed superior hardness (22.1 ± 1.3 GPa) and modulus (239.3 ± 6.5 GPa). These improvements were attributed to solid solution strengthening and amorphization effects.

**Figure 12:**
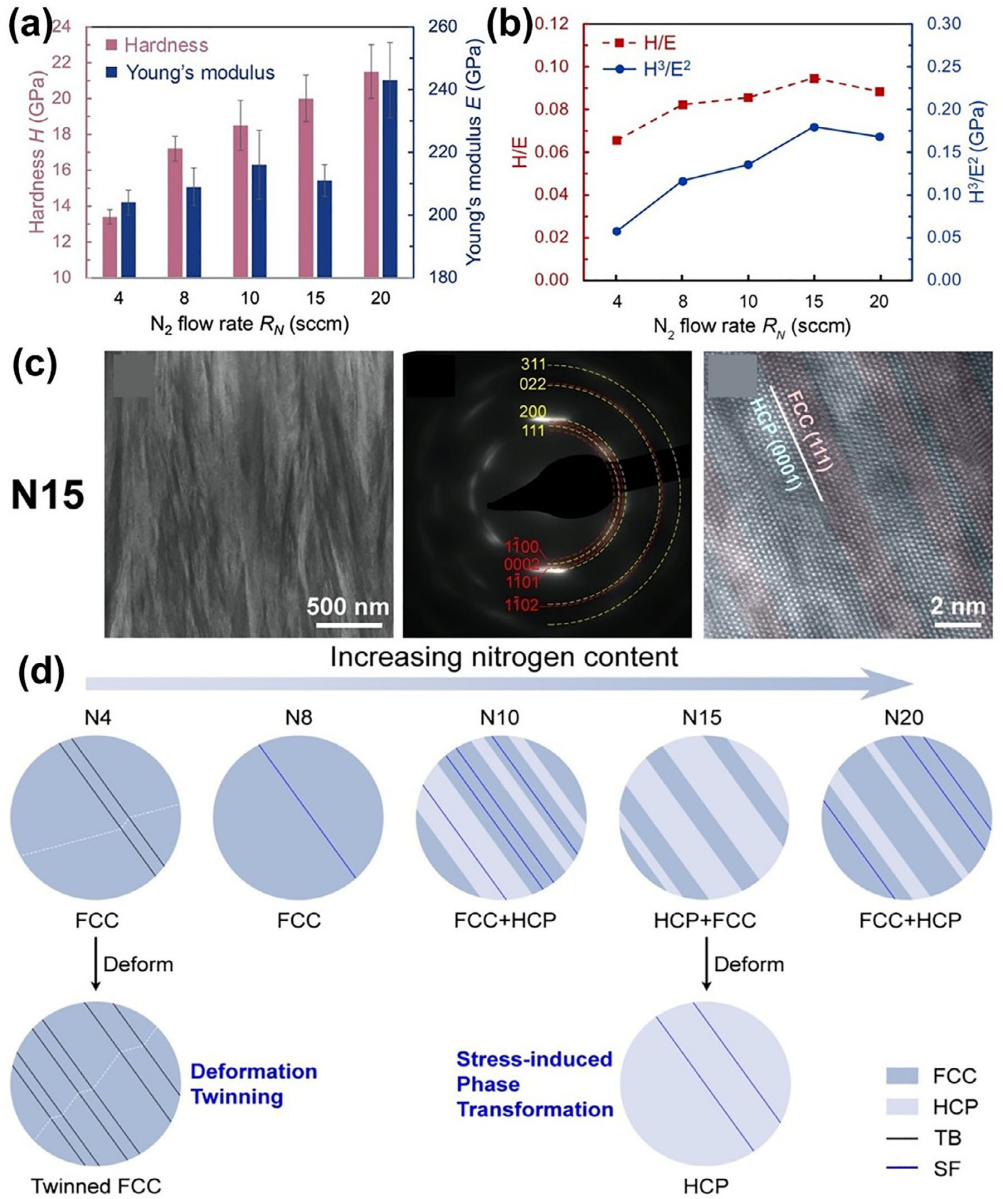
a,b) Mechanical properties of the (Fe_50_Mn_30_Co_10_Cr_10_)N_X_; c) TEM images of the as‐prepared (Fe_50_Mn_30_Co_10_Cr_10_)N_15_; d) Schematic illustration of the microstructural evolution as a function of nitrogen content.^[^ ^143^ ^]^ Copyright 2024, Elsevier.

Due to its excellent solubility and strengthening effects, nitrogen has gained significant attention in MPEA systems. Chen et al.^[^ ^143^ ^]^ synthesized (Fe_50_Mn_30_Co_10_Cr_10_)N_X_ films with distinctive and massive nitrogen contents by varying N_2_ flow rates during magnetron sputtering. As shown in **Figure** **12**, when the N_2_ flow rate was 15 sccm, the films achieved ultra‐high hardness (≈20 GPa), comparable to ceramics, and exceptional damage tolerance due to extensive solid solution strengthening, the coexistence of FCC and HCP phases, and phase‐transformation‐induced plasticity. The highest nitrogen content (20 sccm) yielded peak values of 21.5 GPa hardness and 243 GPa Young's modulus.

**Figure 13:**
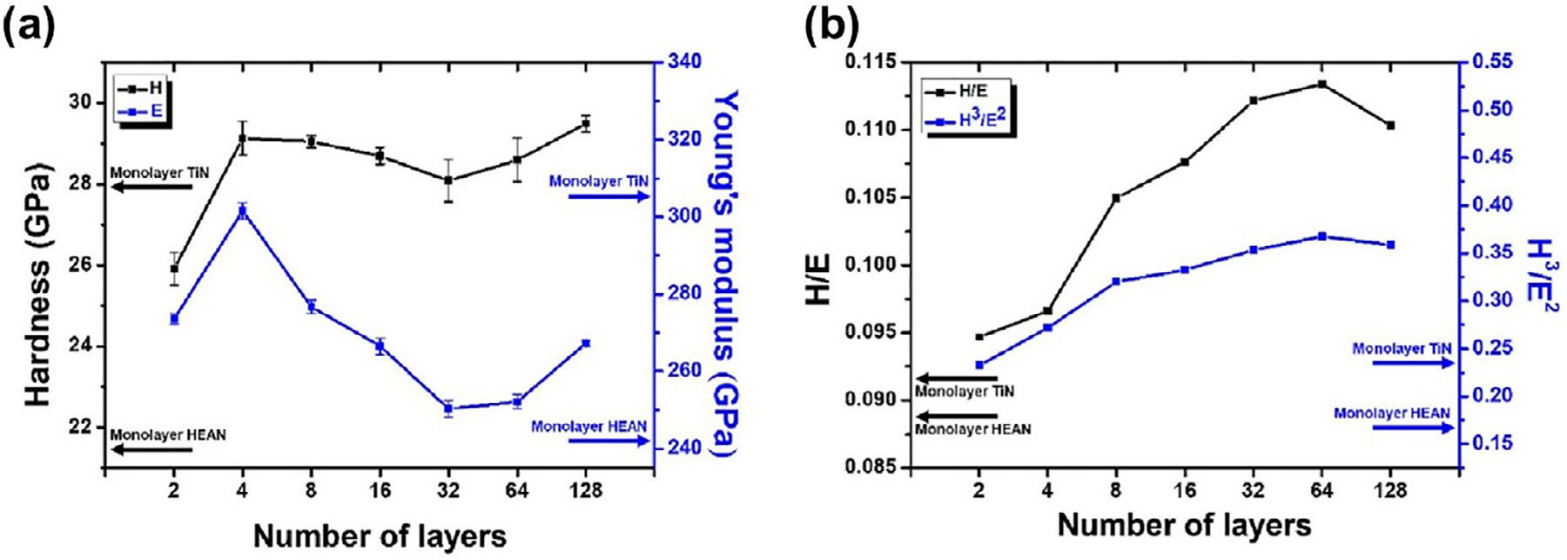
a) Hardness and Young's modulus, and b) H/E, and H^3^/E^2^ values of (AlCrNbSiTi)N/TiN films with different numbers of layers.^[^ ^129^ ^]^ Copyright 2023, Elsevier.

In more complex coating systems, Anupam et al.^[^ ^144^ ^]^ employed atmospheric plasma spray technique to deposit mechanically alloyed AlCoCrFeNi coatings, producing a heterogeneous alloy‐oxide structure. Microscopy and spectroscopy revealed significant compositional and phase variation, with “white” regions corresponding to Al‐depleted Al_14_(CoCrFeNi)_86_, “mottled” regions representing nearly equiatomic Al_2_(CoCrFeNi)_98_, and oxide‐rich “grey” and “black” regions comprising various Al‐Cr‐Fe oxides and alumina splats. Nanoindentation revealed corresponding hardness values of 6 ± 2 GPa (white), 9 ± 2 GPa (grey), and 11 ± 3 GPa (black), illustrating the mechanical heterogeneity arising from phase composition. Lee et al.^[^ ^129^ ^]^ synthesized (AlCrNbSiTi)N/TiN multilayer films using high‐frequency pulsed magnetron sputtering at a high deposition rate. As shown in **Figure** **13**a,b, increasing the number of layers improved hardness through interface strengthening, a refined columnar microstructure, and high‐density interfaces. At 128 layers, the maximum hardness reached 29.5 GPa, accompanied by superior toughness and plasticity.

**Table** **2** summarizes the hardness and elastic modulus of selected MPEA‐based films and coatings. As evident from the data, MPEA compound films and coatings generally exhibit superior hardness and elastic modulus compared to their metallic counterparts, highlighting the critical role of chemical complexity and phase composition. Notably, the deposition method plays a pivotal role in determining film quality. Electrodeposition and DC magnetron sputtering tended to produce relatively inferior coatings compared to the radio frequency magnetron sputtering that enables greater control over film microstructure, producing denser, smoother, and more uniform coatings with improved hardness and modulus. Moreover, increasing the substrate bias voltage during magnetron sputtering has been shown to further enhance the hardness of the films and coatings.^[^ ^145^ ^]^ This is primarily attributed to the densification of the film and the introduction of compressive residual stresses, which collectively improve mechanical strength and deformation resistance.

**Table 2:** Hardness and elastic modulus of MPEA‐based films and coatings.

Composition	Method	Structure	Hardness [GPa]	Modulus [GPa]	Ref.
NiFeCo	Electrodeposition	FCC	5.5	——	[27]
NiFeCr	Electrodeposition	FCC	8.5	——	[27]
HfNbTiVZr	DC magnetron sputtering	BCC	9.2	118	[146]
ZrNbTaTiW	Magnetron sputtering	FCC	11.5	190.4	[147]
AlCoCrCu_0.5_FeNi	RF magnetron sputtering	BCC+FCC	13	204	[148]
Al_2.5_CoCrCuFeNi	DC Magnetron sputtering	BCC	15.4	203.8	[149]
FeAlCuCrCoMn	DC magnetron sputtering	FCC	17.5	186	[140]
Fe_50_Mn_30_Co_10_Cr_10_	Reactive magnetron sputtering	FCC+HCP	21.5	243	[143]
(TiAlCrNbY)C	Reactive DC magnetron sputtering	FCC	22.6	——	[150]
(CuSiTiYZr)C	Reactive magnetron sputtering	FCC	29.5	——	[151]
(AlCrMoTaTiZr)N	Reactive RF magnetron sputtering	FCC	40.2	400	[152]
(AlCrNbSiTiV)N	Reactive RF magnetron sputtering	FCC	41	360	[153]
(CrNbTaTiW)C	DC magnetron sputtering	FCC	36	487	[154]
(AlCrTaTiZr)N	Reactive RF magnetron sputtering	FCC	36.9	——	[155]
(Al_1.5_CrNb_0.5_Si_0.5_Ti)_50_N_50_	Reactive magnetron sputtering	FCC	36	425	[156]
(TiVCrZrHf)N	Reactive magnetron sputtering	FCC	23.8	267.3	[157]
(AlCrMoNiTi)N	Reactive RF magnetron sputtering	FCC	15.4	200	[51]
(AlCrMoZrTi)N	Reactive RF magnetron sputtering	FCC	19.6	236	[51]
(AlCrMnMoNiZr)N	Reactive DC magnetron sputtering	FCC	11.9	202	[50]
(CrTaNbMoV)N	Reactive DC magnetron sputtering	FCC	21.6	241.5	[158]
(TiVCrZrHf)N	Reactive RF magnetron sputtering	FCC	31.4	316.6	[159]
(AlCrNbSiTi)N	Reactive RF magnetron sputtering	FCC	39.8	436.7	[160]
(AlCrNbSiTi)N/TiN	High‐power impulse magnetron sputtering	FCC	29.5	269	[129]
(AlCrNbSiTiMo)N	Reactive RF magnetron sputtering	FCC	34.5	228	[161]
(AlCrTiZrHf)N	Reactive magnetron sputtering	FCC	33.1	347.3	[162]
(MoNbTaVW)N	DC magnetron sputtering	FCC	30	——	[162]
(FeMnNiCoCr)N	DC magnetron sputtering	BCC	17.1	238.5	[163]
(NbTiAlSiW)N	Magnetron sputtering	Amorphous	13.6	154	[164]
(MoSiTiVZr)N	DC magnetron sputtering	FCC	48.4	386.7	[145]

Remarks: RF—radio frequency.

Despite recent advances, only a limited number of MPEA‐based coatings currently achieve hardness values exceeding 40 GPa, which is considered a benchmark for next‐generation ultra‐high hardness materials. This indicates significant untapped potential and pints to ample room for further innovation in the design of high‐performance MPEA‐based films and coatings. Promising strategies to achieve such exceptional performance include doping with interstitial elements such as carbon and nitrogen to promote solid solution strengthening and carbide/nitride formation; refining deposition techniques(e.g., high‐power impulse magnetron sputtering or plasma‐enhanced approaches); optimizing processing parameters (such as gas pressure, substrate temperature, and deposition rate); and engineering nanoscale architectures including multilayers, amorphous/crystalline interfaces, and GB tailoring. Together, these strategies hold great promise for pushing the mechanical limits of MPEA‐based films and coatings to meet the needs of next‐generation applications.

#### 3.1.2 Strength and Plasticity

The deployment of MPEA‐based films and coatings in high‐temperature, high‐stress, and complex service environments demands exceptional mechanical robustness. A critical challenge in this regard lies in the synergistic optimization of high strength and plasticity. Conventionally, strengthening mechanisms tend to embrittle materials, while strategies aimed at enhancing ductility often reduce hardness and wear resistance, compromising long‐term structural integrity and lifespan. To overcome this dilemma, recent studies have focused on tailored microstructural designs, including the introduction of nanoscale features^[^ ^165^, ^166^, ^167^, ^168^ ^]^ (e.g., nanotwins, amorphous domains), dual‐phase architectures,^[^ ^169^, ^170^, ^171^ ^]^ and optimization of deposition parameters.^[^ ^26^, ^172^ ^]^ These efforts offer promising pathways toward concurrent improvements in both strength and plasticity, offering theoretical foundations and practical strategies for applications in extreme conditions.

**Figure 14:**
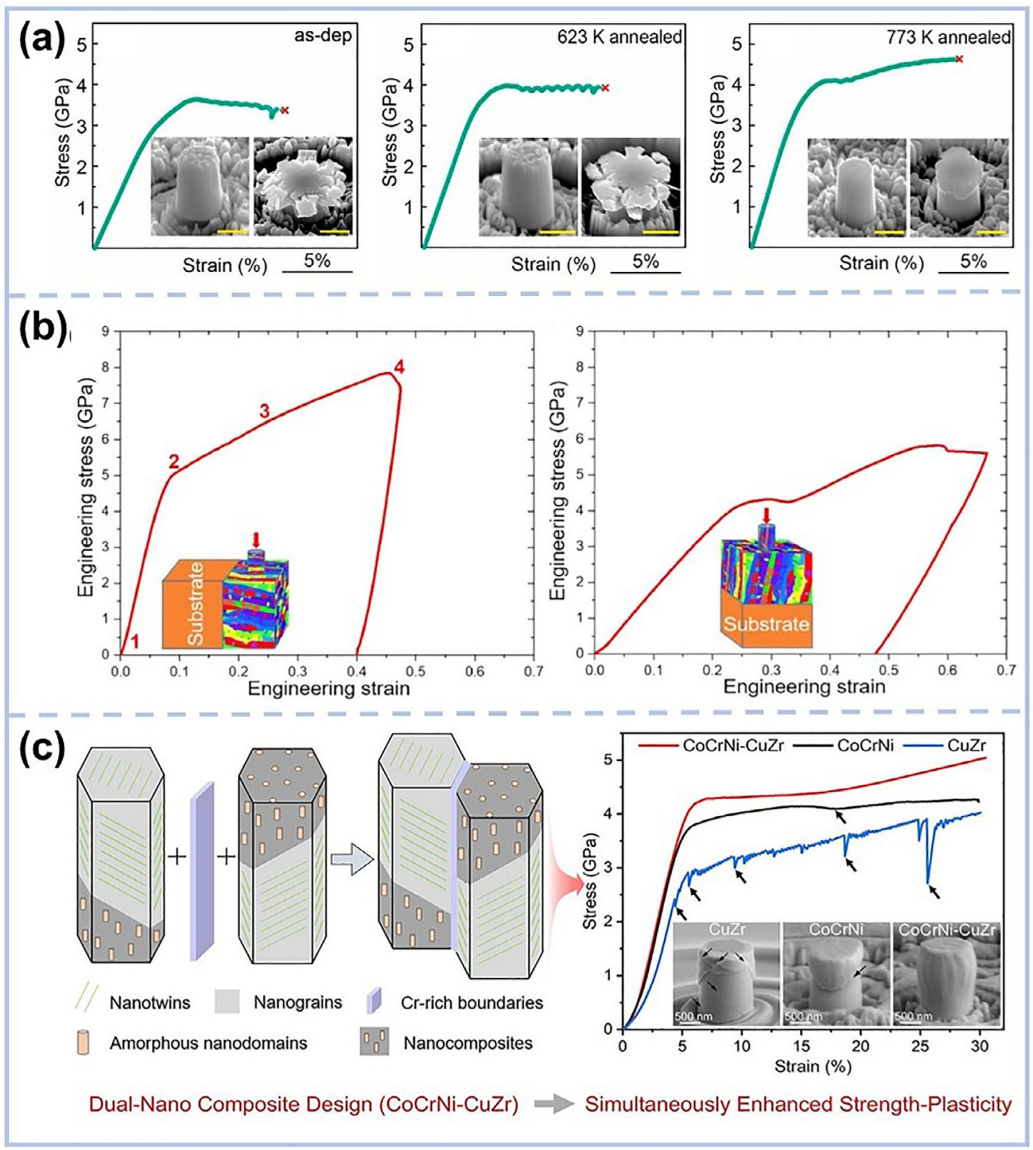
a) Stress–strain curves of as‐dep, 623 K‐annealed, and 773 K‐annealed FeCoNiCrCu film from microcompression tests^[^ ^94^ ^]^ Copyright 2021, Elsevier. b) Engineering stress–strain curves of CrCoNi^[^ ^174^ ^]^ Copyright 2022, Elsevier. c) Mechanical behavior of the as‐deposited (CoCrNi)_91_(CuZr_)9_, CoCrNi, and CuZr films, and the schematic illustration of corresponding deformation mechanisms.^[^ ^175^ ^]^ Copyright 2024, American Chemical Society.

A major breakthrough in addressing the long‐standing strength–plasticity trade‐off in MPEA films lies in the engineering of nano‐twinned structures, a strategy that enables materials to retain, or even enhance, ductility while achieving remarkable strength. Building on pioneering work in nanotwinned copper (NT‐Cu),^[^ ^92^, ^173^ ^]^ researchers have successfully extended this concept to MPEA systems with impressive results. For instance, Zhang et al.^[^ ^94^ ^]^ demonstrated this by synthesizing FeCoNiCrCu MPEA films via magnetron sputtering. Benefiting from synergistic interplay of GB strengthening and nanotwin strengthening (**Figure** **14**a), both as‐deposited and annealed alloy films achieved outstanding yield strength of 3.4–4.2 GPa under microcompression. Notably, moderate annealing at 773 K further improved strain hardening and plasticity without sacrificing strength. These improvements were driven by the formation of deformation twins, triggered by nanoscale chemical fluctuations, which were absent in the as‐deposited and lightly annealed states. Atomistic simulations confirmed that such chemical fluctuations promote twin formation, unlocking the simultaneous enhancement of plasticity and strength.

Pushing the performance boundary further, Gao et al.^[^ ^174^ ^]^ prepared columnar nanocrystalline CrCoNi MPEA films with an average grain size of ≈26 nm via magnetron sputtering. These films not only achieved an ultra‐high yield strength of 5 GPa but also demonstrated an engineering plastic strain exceeding 30% (Figure 14b). Such exceptional plasticity was enabled by a synergetic combination of deformation twinning, grains refinement during deformation, GB sliding, and random grain orientation, providing multiple and complementary mechanisms to accommodate plastic deformation. Chen et al. added another dimension to this narrative by designing hierarchically structured CrCoNi MPEA films embedded with dense twins and stacking faults via DC magnetron sputtering. These films reached yield strengths of up to ≈4 GPa while exhibiting extensive plasticity through a unique microscopic mechanism: a dynamic, reversible FCC–HCP–FCC phase transformation.^[^ ^86^, ^95^, ^98^, ^99^ ^]^ This bidirectional transformation continuously accommodated strain without inducing shear localization.

Expanding this frontier further, Zhuang et al.^[^ ^175^ ^]^ engineered (CoCrNi)_91_(CuZr)_9_ MPEA films by selecting low‐SFE CoCrNi and an amorphous CuZr alloy, while subtly optimizing the sputtering process parameters. The resulting films exhibited a typical columnar nanostructure containing embedded nanocrystalline‐amorphous dual‐phase structure domains (Figure 14c). Micropillar compression tests revealed an ultrahigh yield strength of 4.0 ± 0.2 GPa, which was 18% higher than nanocrystalline CoCrNi and 67% higher than amorphous CuZr, while maintaining uniform deformation beyond 30% (Figure 14c). This impressive performance was attributed to a trifecta of sub‐nanometer twins, embedded amorphous phases, and Cr‐enriched GBs, collectively suppressing strain localization stabilized plastic flow.

**Figure 15:**
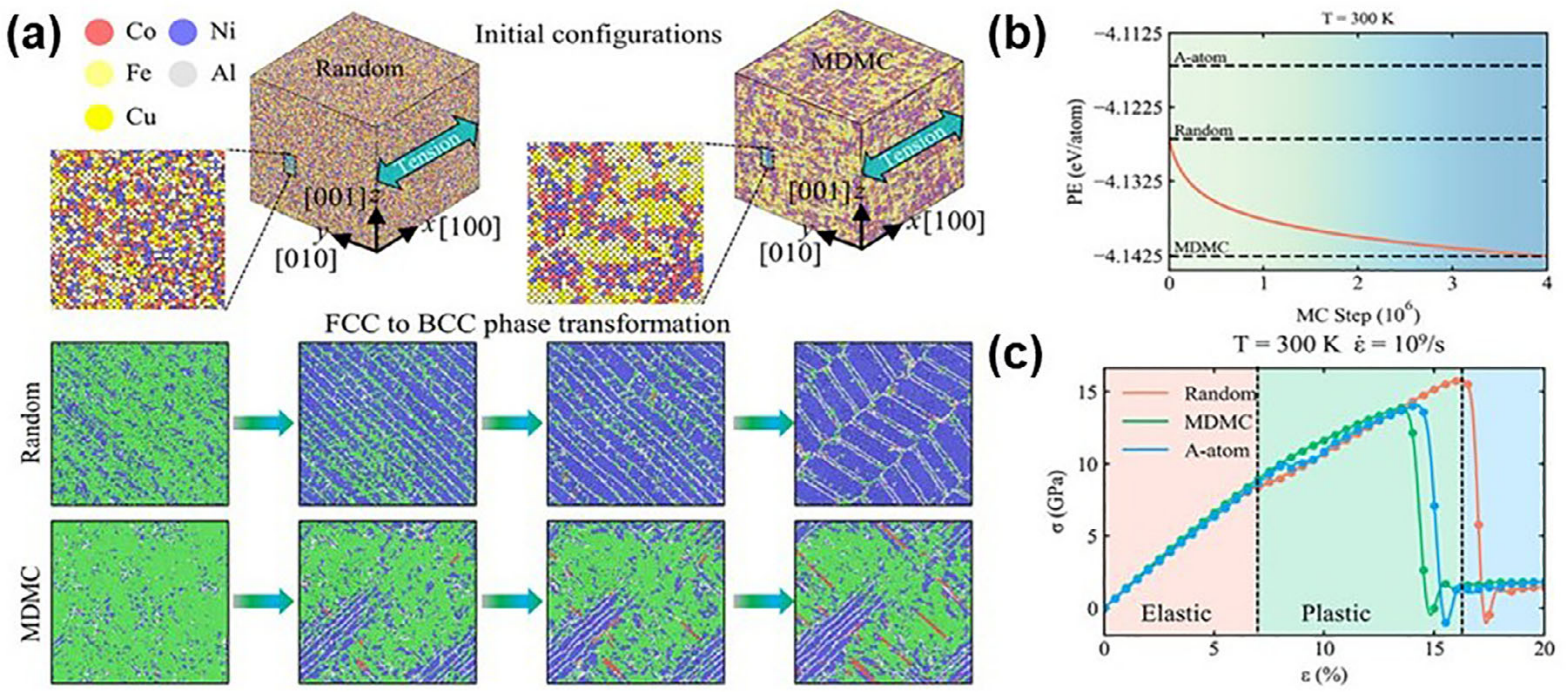
a) Microstructures of the Random and MDMC samples; b) variation of potential energy during hybrid MD/MC relaxation at T = 300 K; c) Stress–strain curve of Co_25_Ni_25_Fe_25_Al_7.5_Cu_17.5_ MPEA with different configurations (Random: fully relaxed; MDMC: after Monte Carlo and molecular dynamics relaxation and A‐atom: a virtual element sample with an average‐atom interatomic potential).^[^ ^176^ ^]^ Copyright 2023, Elsevier.

Building upon the success of nanotwinned architectures, recent research has increasingly focused on multi‐level microstructural engineering, such as integrating additional strengthening and toughening mechanisms, such as stress‐driven phase transformations, local compositional engineering, interstitial solid solution strengthening, and heterogeneous amorphous layering. These approaches, often working synergistically with twinning, introduce tailored phase compositions, controlled lattice distortions, and hierarchical interfaces to enable superior mechanical resilience. By combining these design strategies, MPEA films and coatings can achieve unprecedented combinations of ultrahigh strength and extensive plastic deformation, offering promising solutions for next‐generation structural and protective materials. For instance, while FCC‐structured MPEAs are typically ductile but relatively soft, and BCC‐based ones are strong yet brittle, researchers have increasingly turned to hybrid strategies that combine the merits of both crystal structures. Wu et al.^[^ ^176^ ^]^ investigated a single‐phase FCC Co_25_Ni_25_Fe_25_Al_7.5_Cu_17.5_ MPEA using integrated Monte Carlo and molecular dynamics simulations to probe the atomic‐scale mechanisms of phase transformation under tensile loading (**Figure** **15**a). Their study revealed that under ultra‐high strain rates and across a wide temperature range, a stress‐induced FCC‐to‐BCC phase transformation occurred, enhancing both fracture strength and plasticity (Figure 15b,c). The findings highlight the potential of phase transformation pathways to unlock superior mechanical performance in FCC‐based MPEAs by leveraging the mechanical merits of BCC structures.

Sophisticated local compositional engineering has also shown great promise. Liu et al.^[^ ^19^ ^]^ introduced Ni into a nanocrystalline (TiZrNbHf)_98_Ni_2_ alloy by magnetron sputtering, strategically placing Ni both at GBs and within grains. Due to Ni's strong negative mixing enthalpy and contrasting electronegativity relative to base elements, it created chemical inhomogeneity within nanograins and modified GB chemistry. The combination of GB segregation and localized chemical inhomogeneity within the nanocrystals enabled the nanocrystalline alloy to achieve ultrahigh strength and ductility. The resultant alloy achieved a 2.5 GPa yield strength, together with work hardening capability and large homogeneous deformability to 65% compressive strain, a rare and valuable combination for nanocrystalline alloys.

**Figure 16:**
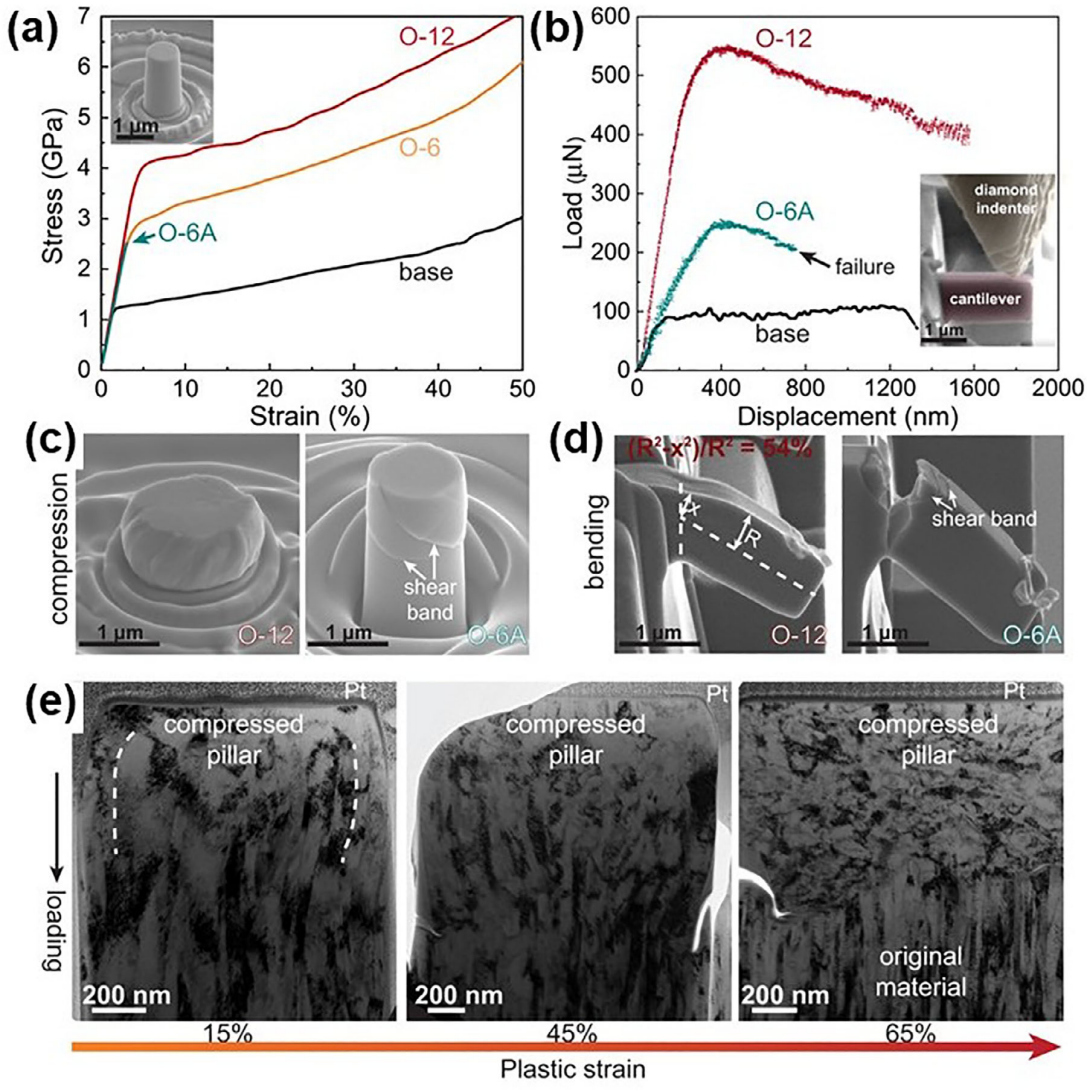
a) Compressive engineering stress–strain of TiNbZr alloy (base), ((TiNbZr)_92_O_6_C_1_N_1_ (O‐6 and O‐6A indicating the alloy with amorphous structure), and O‐12 (TiNbZr)_86_O_12_C_1_N_1_ (O‐12); b) Load‐displacement curves measured by bending of cantilever beams of base, O‐6A, and O‐12 alloys; SEM of the alloys after deformation: c) micro‐pillars and d) cantilevers of O‐12 and O‐6A alloys; e) Microstructural evolution of O‐6A alloy with increasing compressive strains.^[^ ^85^ ^]^ Copyright 2022, Springer Nature.

Interstitial solid solution strengthening offers yet another route because interstitial elements, such as carbon, nitrogen, and oxygen, create intense lattice distortions, effectively hindering dislocation motion and thereby strengthening metals significantly. Liu et al.^[^ ^85^ ^]^ leveraged this strategy to develop BCC‐type (TiNbZr)_86_O_12_C_1_N_1_ alloy using magnetron sputtering, incorporating a high concentration of oxygen (12 at%) along with minor additions of carbon and nitrogen. Remarkably, despite the high oxygen content, no ceramic or oxide phase were observed. The alloy exhibited an ultrahigh yield strength of 4.2 GPa under compression, approaching the theoretical strength limit (**Figure** **16**a–d). This near‐theoretical compressive strength stemmed from the tremendous dislocation‐impeding effect of interstitial oxygen atoms, while carbon and nitrogen segregated to GBs, suppressing strain localization. Under micropillar compression, these mechanisms facilitated deformation‐driven grain refinement, enabling the material to sustain up to an exceptional strain of ≈65% without localized shear failure (Figure 16e).

In addition to composition and phase manipulation, heterostructuring has emerged as a powerful approach. In a study by,^[^ ^135^ ^]^ researchers fabricated a heterogeneous nanolayer amorphous film via alternating magnetron sputtering. The introduction of distinct amorphous layers harnessed intrinsic size effects to suppress shear banding, a common failure mode in metallic glasses. The result was a yield strength of 1.6 GPa paired with ≈40% homogeneous plastic deformation, effectively improving formability without compromising strength.

The remarkable advancements in microstructural and compositional design of MPEA‐based films have opened new pathways toward achieving the long‐sought synergy between strength and plasticity. Abovementioned innovative strategies have demonstrated that it is indeed possible to transcend traditional trade‐offs and unlock exceptional mechanical resilience. Despite these promising developments, the field is still in its early stages, with many fundamental questions yet to be answered. Key knowledge gaps remain in understanding the interplay between complex chemical environments, defect dynamics, and deformation mechanisms at the nanoscale. Moreover, the role of metastable phases and interface‐driven phenomena in dictating plasticity and failure resistance remains underexplored. Addressing these challenges will require a concerted effort combining high‐resolution characterization, advanced simulations, and data‐driven design methodologies. The continued evolution of MPEA‐based films offers a bright and transformative future for structural and functional materials, especially in applications demanding reliability, adaptability, and performance under harsh service environments.

#### 3.1.3 Fatigue Properties

Ensuring long‐term structural integrity under cyclic loading is a fundamental requirement for advanced materials in critical structural applications such as aerospace, automotive, and microelectromechanical systems. Fatigue failure, often sudden and catastrophic, remains one of the most critical challenges in engineering design, accounting for a substantial portion of mechanical failures in service environments.^[^ ^177^, ^178^, ^179^, ^180^ ^]^ As a new frontier in structural materials, MPEA films and coatings hold immense promise for fatigue‐critical applications, owing to their exceptional strength‐ductility synergy and thermal stability.^[^ ^181^, ^182^ ^]^

However, the path to unlocking their full potential is hindered by major technical barriers, particularly in characterizing fatigue behavior at small scales. Fatigue in thin films and coatings is typically triggered by cyclic strain accumulation arising from thermomechanical mismatches between the film and substrate.^[^ ^183^ ^]^ These localized stress concentrations can lead to premature crack initiation and propagation. Yet, the conventional uniaxial fatigue testing methods used for bulk materials are unsuitable for such confined geometries, making accurate and reproducible fatigue assessment especially challenging.

To address these challenges, Wang et al.^[^ ^96^ ^]^ pioneered the use of nanoindentation‐based dynamic mechanical analysis to investigate the fatigue response of CoCrFeMnNi MPEA films. These films, fabricated via radio frequency magnetron sputtering without substrate bias, contained high‐density nanotwins, which were hypothesized to enhance fatigue resistance. Remarkably, the fatigue strength trends derived from nanoindentation closely aligned with those of traditional uniaxial fatigue tests, validating this approach for nanoscale evaluation. Furthermore, the presence of nanotwins was shown to facilitate fatigue endurance via detwinning mechanisms during cyclic loading, effectively delaying crack formation and promoting stable plastic accommodation.

Looking forward, our current understanding of fatigue mechanisms in MPEA‐based films and coatings remains in its infancy, leaving a critical gap between their remarkable intrinsic properties and reliable long‐term performance, which represents a vital and exciting research direction. Emerging characterization tools, combined with tailored microstructural design,^[^ ^124^, ^184^, ^185^, ^186^, ^187^, ^188^, ^189^, ^190^, ^191^ ^]^ such as nanotwinning, gradient architectures, and heterophase interfaces, offer tremendous potential to engineer MPEA films and coatings with unprecedented fatigue performance. As testing techniques continue to evolve, the path toward fatigue‐resistant, application‐ready MPEA systems will become increasingly viable. By bridging this knowledge gap, we can accelerate the deployment of MPEA‐based films in next‐generation structural, protective, and functional applications where cyclic durability is not just desirable, but essential.

### 3.2 Corrosion Resistance

The corrosion resistance of MPEAs is one of their most promising attributes, making them ideal candidates for demanding environments where material degradation is a critical concern. Their unique combination of high‐entropy effects, lattice distortion, sluggish diffusion, and synergistic elemental interactions foster highly uniform and stable microstructures. These features not only slow down corrosive processes but also enable the formation of robust passive films. As thin films and coatings, MPEAs exhibit enhanced surface uniformity and tailored chemistries, offering a new class of advanced materials for applications in marine, biomedical, energy, and aerospace systems where corrosion protection is paramount.

Thanks to rapid quenching and high‐entropy stabilization, MPEA‐based coatings typically adopt either a single‐phase solid solution or an amorphous structure, both of which are highly advantageous for corrosion resistance. The single‐phase solid solution promotes a homogeneous elemental distribution, minimizing local electrochemical potential differences and suppressing galvanic corrosion. Meanwhile, the amorphous structure, devoid of GBs and dislocations, offers a highly stable and inert surface, effectively blocking corrosion pathways. For instance, Dou et al.^[^ ^192^ ^]^ reported that FeAlCoCuNiV coatings deposited by DC magnetron sputtering, with a simple FCC structure and no elemental segregation, outperformed 201 stainless steel in corrosive environments including 3.5% NaCl, 5% NaOH, and 10% H_2_SO_4_.

Beyond structure, the deliberate selection and tuning of elemental constituents remains a powerful strategy to enhance corrosion resistance. Elements like Cr, Ni, Al, and Hf are particularly effective in promoting the formation of stable passive films. Huo et al.^[^ ^61^ ^]^ showcased this by electrodepositing CoNiFe films with varying microstructures, where the film deposited at a current density of 44.4 A dm^−2^ exhibited a nearly equimolar chemical composition and a strong (111) FCC texture. In a 3.5 wt.% NaCl solution, this film achieved an impressively low self‐corrosion current density was 4.72 × 10^−6^ A cm^−2^ using electrochemical impedance spectroscopy (EIS) tests, indicating exceptional corrosion resistance. Similarly, Lu et al.^[^ ^193^ ^]^ developed FCC‐structured CoCrFeNiAl_0.3_ films using pulsed laser sputtering, which displayed markedly better corrosion resistance than 316L stainless steel. Moreover, the integration of corrosion‐resistant elements can drastically alter microstructure and performance. Zhang et al.^[^ ^142^ ^]^ explored the influence of Hf addition in NbTaW films, using multi‐target DC magnetron co‐sputtering to fabricate both NbTaW and NbTawHf variants. The incorporation of Hf transformed the film into a fully amorphous and dense structure, free of GBs and dislocations, which critically enhanced corrosion resistance. As a result, the NbTaWHf coatings achieved corrosion current densities nearly two orders of magnitude lower than those of 304 stainless steel, alongside significantly improved polarization resistance.

**Figure 17:**
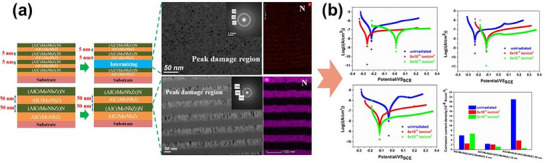
a) Cross‐sectional TEM image of as‐deposited AlCrMoNbZr/(AlCrMoNbZr)N multilayer coating; b) Corrosion resistance of AlCrMoNbZr/(AlCrMoNbZr)N multilayer coating in 3.5% NaCl solution.^[^ ^194^ ^]^ Copyright 2019, Elsevier.

Multilayer architectures represent another effective strategy for enhancing corrosion resistance, as their abundant interfaces act as formidable barriers to defect migration, crack propagation, and the diffusion of corrosive species. As shown in **Figure** **17**a, Zhang et al.^[^ ^194^ ^]^ systematically investigated AlCrMoNbZr/(AlCrMoNbZr)N multilayer coatings prepared by magnetron co‐sputtering with varying bilayer periods. Compared with single‐layer coatings, the multilayer design offered substantially improved corrosion performance. Notably, the 50/50 nm multilayer structure exhibited the highest resistance against high‐temperature water corrosion (360 °C, 18.7 MPa, 30 days), attributed to its ability to suppress Al migration and inhibit the formation of boehmite (AlOOH). The alternating amorphous AlCrMoNbZr and FCC (AlCrMoNbZr)N layers also functioned as defect sinks and barriers to crack propagation, further reinforcing long‐term stability of the coating (Figure 17b).

Extending this concept, Zhang et al.^[^ ^195^ ^]^ examined hybrid‐structured (TiTaNbZrNi)N coatings produced by reactive gas pulse sputtering. The incorporation of multilayer architecture with compositionally graded regions prominently reduced oxygen diffusion depth, yielding a weight gain an order of magnitude lower than that of uncoated Zr alloys after 50 days of autoclave testing (360 °C, 18.6 MPa). The periodic interfaces served as diffusion barriers, while compositional gradients optimized the chemical environment, jointly suppressing localized corrosion. This highlights the effectiveness of synergistic microstructural tailoring, particularly through multilayering, in drastically boosting corrosion resistance.

The significance of interface stability under extreme conditions was further demonstrated by Zhang et al.^[^ ^196^ ^]^ who investigated AlCrMoNbZr/(AlCrMoNbZr)N multilayer coating prepared by magnetron sputtering under irradiation. They found that coatings with thicker bilayer periods (50 nm) retained sharper interfaces and maintained higher corrosion resistance than thinner multilayers (5 nm) after high‐fluence He ion irradiation. This enhanced performance originated from the ability of stable interfaces to block defect migration and mitigate irradiation‐induced degradation. Collectively, these results underscore that multilayer architectures not only improve corrosion resistance in conventional environments but also deliver durability under extreme service conditions, including irradiation. By suppressing detrimental elemental migration, blocking diffusion pathways, and stabilizing microstructures, multilayer design enables MPEA coatings to achieve long‐term chemical and structural integrity. This positions multilayering as a versatile and forward‐looking structural engineering strategy with exceptional promise for next‐generation corrosion‐resistant coatings.

**Figure 18:**
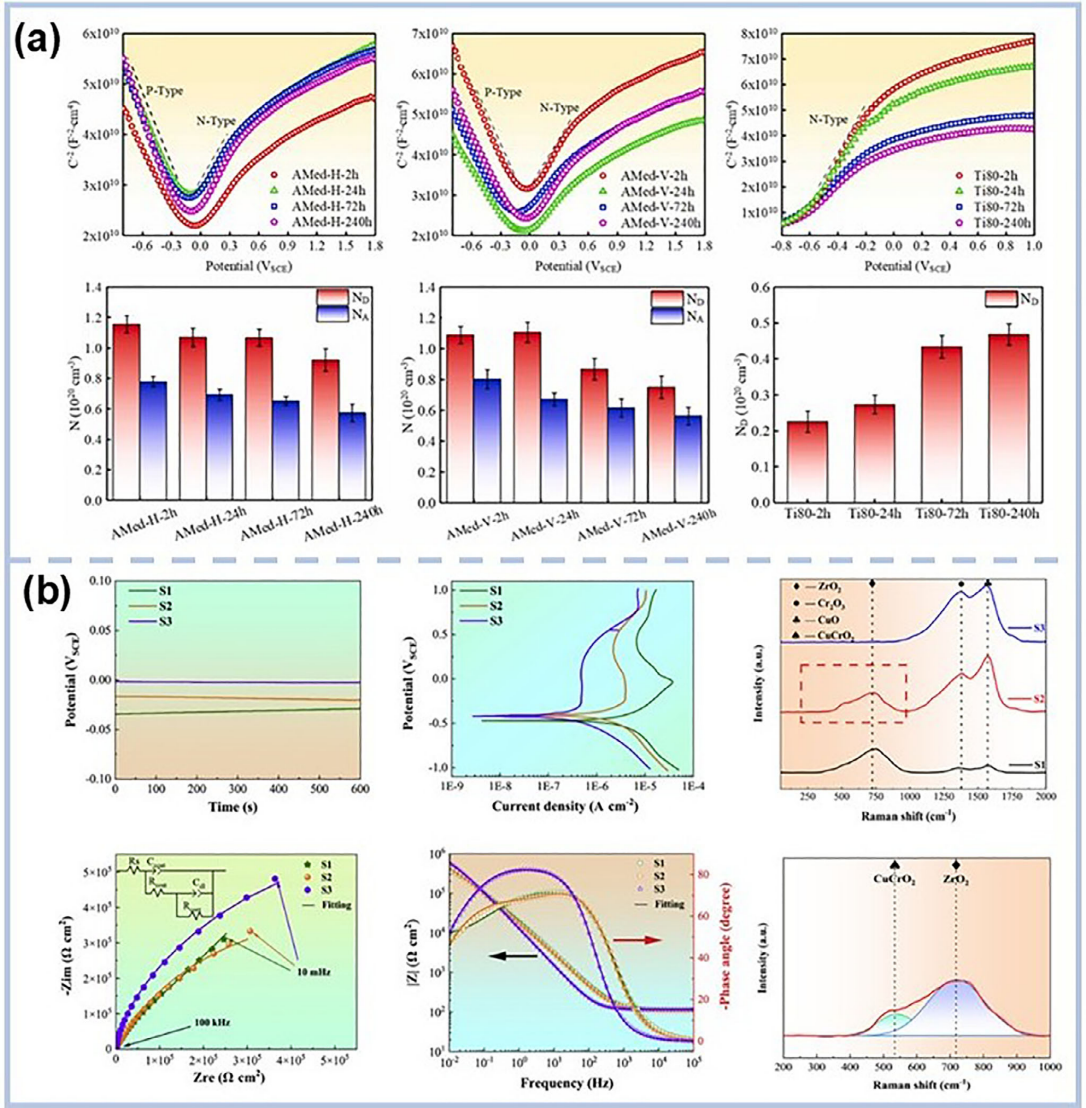
a) Mott–Schottky results of the TiVZrNbAl films immersed in 0.6 m NaCl solution at room temperature for different times^[^ ^197^ ^]^ Copyright 2024, Elsevier. b) Electrochemical corrosion tests and Raman spectra of the ZrNbTiCrCu films.^[^ ^16^ ^]^ Copyright 2025, Elsevier.

To further expand the application of MPEAs in corrosive environments, there is growing interest in designing lightweight, corrosion‐resistant MPEA‐based films and coatings for advancing structural and biomedical applications by incorporating elements such as Ti, V, Zr, Ta, Nb, and Al. These elements not only reduce density but also contribute to the formation of stable passive films and corrosion‐resistant microstructures. For instance, Li et al.^[^ ^197^ ^]^ investigated the corrosion behavior of BCC‐structured Ti_45_V_30_Zr_12_Nb_12_Al_1_ films fabricated by laser direct energy deposition in 0.6M NaCl solution, comparing their performance to conventional titanium alloys (Ti‐6Al‐3Nb‐2Zr‐1Mo and Ti80 alloy). As illustrated in **Figure** **18**a, the Mott‐Schottky analysis revealed that for the Ti‐based MPEA films (AMed‐H and AMed‐V), both donor density (ND) and acceptor density (NA) decreased over time, suggesting a gradual enhancement in the stability of the passive film, a phenomenon referred to as the self‐stabilization effect. In contrast, Ti80 exhibited an increasing ND over time, indicating degradation of its passive film stability. The enhanced stability in the MPEA‐based films was attributed to doping with high‐valence cations, which reduce vacancy defects in the passive film and enhance its protective capabilities.

Further innovations in composition and process control have yielded similarly promising results. Ma et al.^[^ ^16^ ^]^ optimized sputtering parameters to fabricate ZrNbTiCrCu films with a BCC structure and Cu‐rich phases. As shown in Figure 18b, the addition of Cu significantly enhanced the corrosion resistance of the film in simulated body fluid, reducing the corrosion current density to 4.0 × 10^−7^ A cm^−2^. This improvement was attributed to the formation of a new protective CuCrO_2_ bimetallic oxide within the passive film, which greatly reinforced both its stability and protective function.

In addition to metallic elements, the incorporation of carbon offers another pathway to densify the microstructure, reduce porosity, and thereby enhance corrosion resistance. Braic et al.^[^ ^151^ ^]^ investigated the effect of carbon content on the corrosion behavior of (CuSiTiYZr)C_X_ films deposited by magnetron sputtering and evaluated in 3.5% NaCl electrolyte. The results showed that increasing carbon content enhanced corrosion resistance, largely due to the formation of amorphous free carbon. This amorphous phase inhibited carbide crystallization, refined overall microstructure, and reduced porosity, significantly contributing to the observed corrosion resistance improvement.

Enhancing surface quality and film density is another effective strategy for improving the corrosion resistance of MPEA‐based films and coatings. A denser, more uniform morphology minimizes pathways for corrosive agents, while reducing structural defects that often act as initiation sites for corrosion. Hsueh et al.^[^ ^198^ ^]^ prepared (AlCrSiTiZr)_100‐X_N_X_ films using DC reactive magnetron sputtering and investigated the effects of nitrogen flow rate and substrate bias on the corrosion resistance of the films. Without substrate bias, the corrosion resistance peaked at a nitrogen flow rate of 30%. When the substrate bias of −100 V was applied, the corrosion resistance was further enhanced. This improvement was originated from film densification triggered by the substrate bias and the introduction of beneficial compressive stress, both of which helped suppress corrosive degradation.

Similarly, Li et al.^[^ ^145^ ^]^ deposited (MoSiTiVZr)N coatings via DC reactive sputtering, applying varying levels of a radio frequency bias (0–150 W). Increasing bias power led to ion bombardment and surface etching, transforming the film morphology from a columnar structure to a featureless dense structure. At a bias power of 100 W, the coating achieved the lowest corrosion current density of 2.45 × 10^−8^ A cm^−2^, indicating optimal corrosion resistance due to its compact, defect‐free surface. Yuki et al.^[^ ^28^ ^]^ demonstrated a green and effective route for successfully electrodepositing CrCoNi coatings. By using a mixed ionic and aqueous electrolyte and carefully controlling the deposition potential, they achieved a smooth and dense metallic coating. Compared to conventional hard Cr coatings electrodeposited from toxic Cr(VI)‐containing solutions, the CrCoNi coatings exhibited not only a more environmentally friendly process but also significantly superior corrosion resistance.

Despite the significant progress achieved, the field of corrosion‐resistant MPEA‐based films and coatings remains in its early stages, with critical knowledge gaps yet to be addressed. The underlying mechanisms that govern corrosion resistance, particularly pertinent to the formation, evolution, and breakdown of passive films, are still not fully understood. A systematic exploration of how non‐corrosion‐resistant elements affect overall performance, as well as how synergistic elemental interactions influence film stability, is urgently needed. Moreover, the complex relationship between processing conditions, resulting microstructure, and corrosion behavior demands deeper investigation through well‐designed, high‐throughput experiments. Within this context, microstructural tailoring strategies such as multilayer architectures have shown great promise, but their effectiveness in ensuring long‐term passivation stability, resisting irradiation damage, and mitigating localized corrosion still demands comprehensive evaluation. To advance the field, the development of advanced in situ and operando characterization techniques capable of tracking passivation, degradation kinetics, and nanoscale corrosion processes in real time will be crucial. Integrating these experimental approaches with multiscale modeling and machine learning could provide unprecedented insight into corrosion mechanisms and allow the establishment of accurate composition‐structure‐property‐processing correlations. Looking forward, such an integrated framework will unlock the full potential of MPEAs, enabling the rational design of coatings with tunable chemistries, dense and defect‐resistant microstructures, and optimized passive behavior. More importantly, it will deliver protection solutions that not only extend durability under harsh service conditions but also surpass the limits of conventional alloy systems, positioning MPEA films and coatings as a transformative platform for next‐generation corrosion‐resistant technologies.

### 3.3 Wear and Erosion Resistance

As advanced engineering systems are increasingly deployed in extreme service environments, characterized by heavy mechanical loads, fluctuating thermal cycles, sustained frictional contact, and often high‐velocity particle or liquid impingement, the demand for surface materials with superior resistance to both sliding wear and erosion has become more pressing than ever. Mechanical wear and particle‐ or jet‐induced erosion not only undermine the structural integrity of critical components but also accelerate system degradation and escalate maintenance costs. In response to these challenges, MPEA‐based films and coatings have emerged as highly promising candidates owing to their exceptional hardness, thermal stability, and compositional flexibility. Their unique multi‐element chemistry allows fine‐tuning of strength‐toughness synergies, rendering them particularly well suited for complex tribological and erosive conditions. Realizing this potential, however, requires a comprehensive understanding of how elemental selection, microstructural design, deposition techniques, and interfacial properties collectively govern frictional wear and erosion resistance. Such insights are essential for optimizing the performance of MPEA coatings in demanding engineering applications and for guiding the rational design of next‐generation protective surfaces.

The strategic incorporation of specific alloying elements plays a pivotal role in tailoring the microstructure and enhancing the wear resistance of MPEA‐based films and coatings. By carefully selecting elements such as Mo, V, Al, and N, it is possible to drive the formation of higher‐strength grain structures and phase compositions that contribute to superior tribological performance. These elements promote phase transformations from relatively soft FCC structures to harder BCC or amorphous phases, which are inherently more resistant to plastic deformation and abrasive wear. Combined with solid solution strengthening, refined grain size, and enhanced lattice distortion, these synergistic effects lead to increased surface hardness, reduced wear rates, and improved mechanical integrity under harsh contact conditions. For instance, Zhao et al.^[^ ^199^ ^]^ demonstrated that Mo addition to CoCrFeNiMn films led to significant grain refinement and densification during deposition, accompanied by a phase transition from a single FCC to a dual FCC+BCC phase. The incorporation of larger Mo atoms caused intensified lattice distortion, increasing the film's hardness from 8.5 to 12 GPa and significantly enhancing its damage tolerance. Wear tests revealed that the films with higher Mo content exhibited reduced wear debris and shallower plowing grooves, verifying the substantial enhancement in wear resistance. Similarly, Fang et al.^[^ ^200^ ^]^ reported that the introduction of a small amount of V element induced the formation of nanoscale twins in CoCrFeNiMn films prepared by RF magnetron co‐sputtering. These nanotwin structures acted as effective barriers to dislocation motion, thereby enhancing the work‐hardening ability, increasing both hardness and yield strength, and significantly improving the film's resistance to plastic deformation under tribological loading.

**Figure 19:**
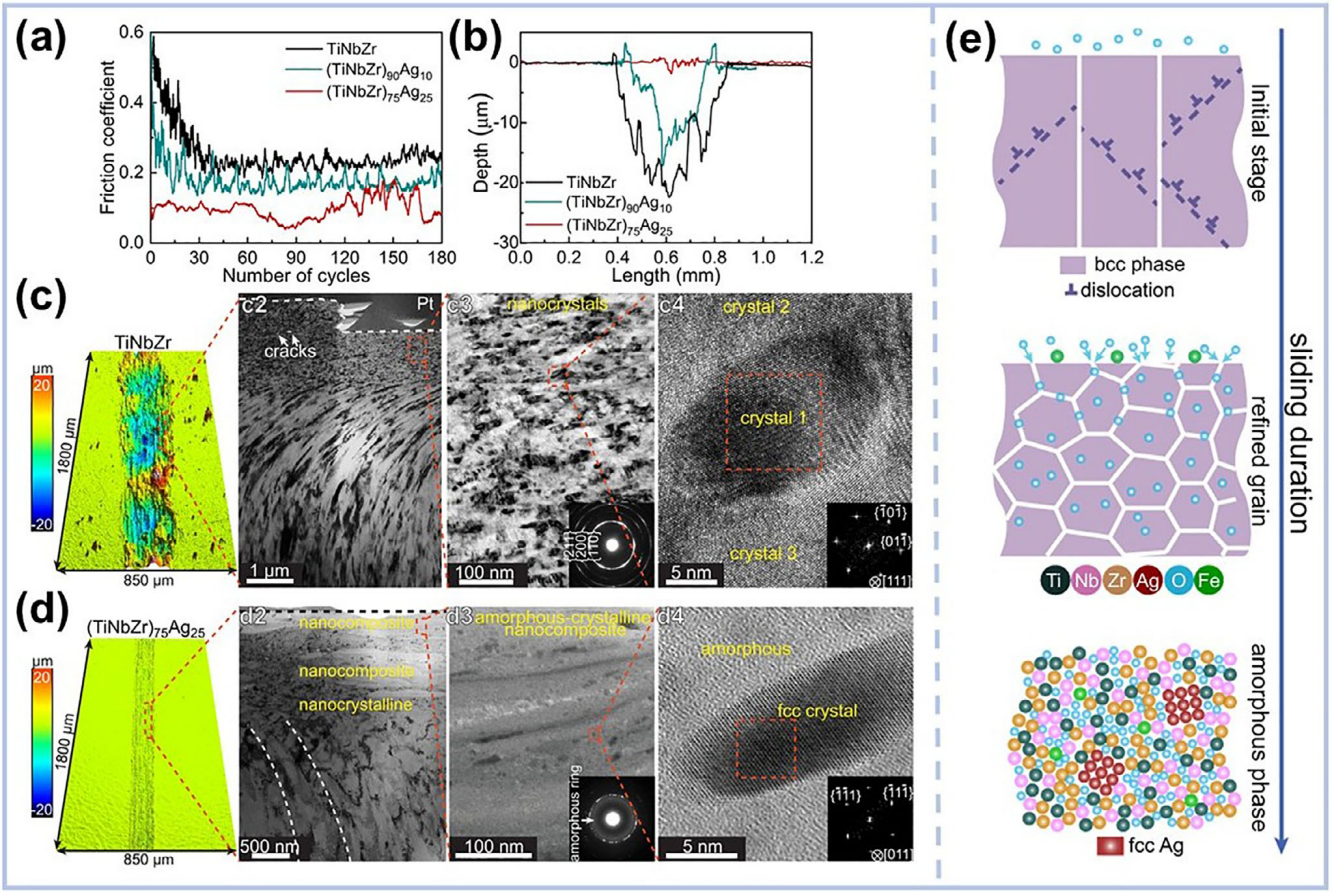
a–d) Wear behavior of TiNbZr, (TiNbZr)_90_Ag_10_, and (TiNbZr)_75_Ag_25_ alloys; e) Formation mechanism of amorphous‐crystalline oxide nanocomposite during wear test.^[^ ^201^ ^]^ Copyright 2021, Springer Nature.

To further enhance the wear resistance of MPEA‐based films and coatings, emerging design strategies have begun to leverage tribochemical effects and nanostructured architectures. These approaches focus on tailoring the alloy composition and layer structure to induce self‐adaptive behaviors under friction, such as the in situ formation of protective oxide layers or the development of self‐lubricating interfaces. By intelligently incorporating reactive elements and lubricating agents, these innovations significantly extend the mechanical durability and tribological performance of MPEAs under extreme service conditions. Liu et al.^[^ ^201^ ^]^ proposed a novel wear‐resistant MPEAs design strategy that utilized friction‐induced reaction between the alloy surface and ambient oxygen to form an in situ amorphous‐nanocrystalline composite oxide layer. This layer exhibited high strength and uniform plastic deformation, significantly improving the wear resistance (**Figure** **19**a–d). Specifically, (TiNbZr)_75_Ag_25_ MPEAs fabricated via magnetron sputtering formed a nanocomposite surface during friction in air, the in situ‐formed, where amorphous oxides encapsulated Ag nanocrystals (≈10 nm) (Figure 19e). This structure exhibited an ultrahigh yield strength of 2.4 GPa and could sustain up to 20% strain in nano‐compression tests, leading to a wear rate that was two orders of magnitude lower than TiNbZr under identical friction conditions, and one order of magnitude lower than the same alloy tested in an Ar atmosphere, and thereby demonstrating the effectiveness of this “reactive wear reduction” mechanism (Figure 19a,b).

**Figure 20:**
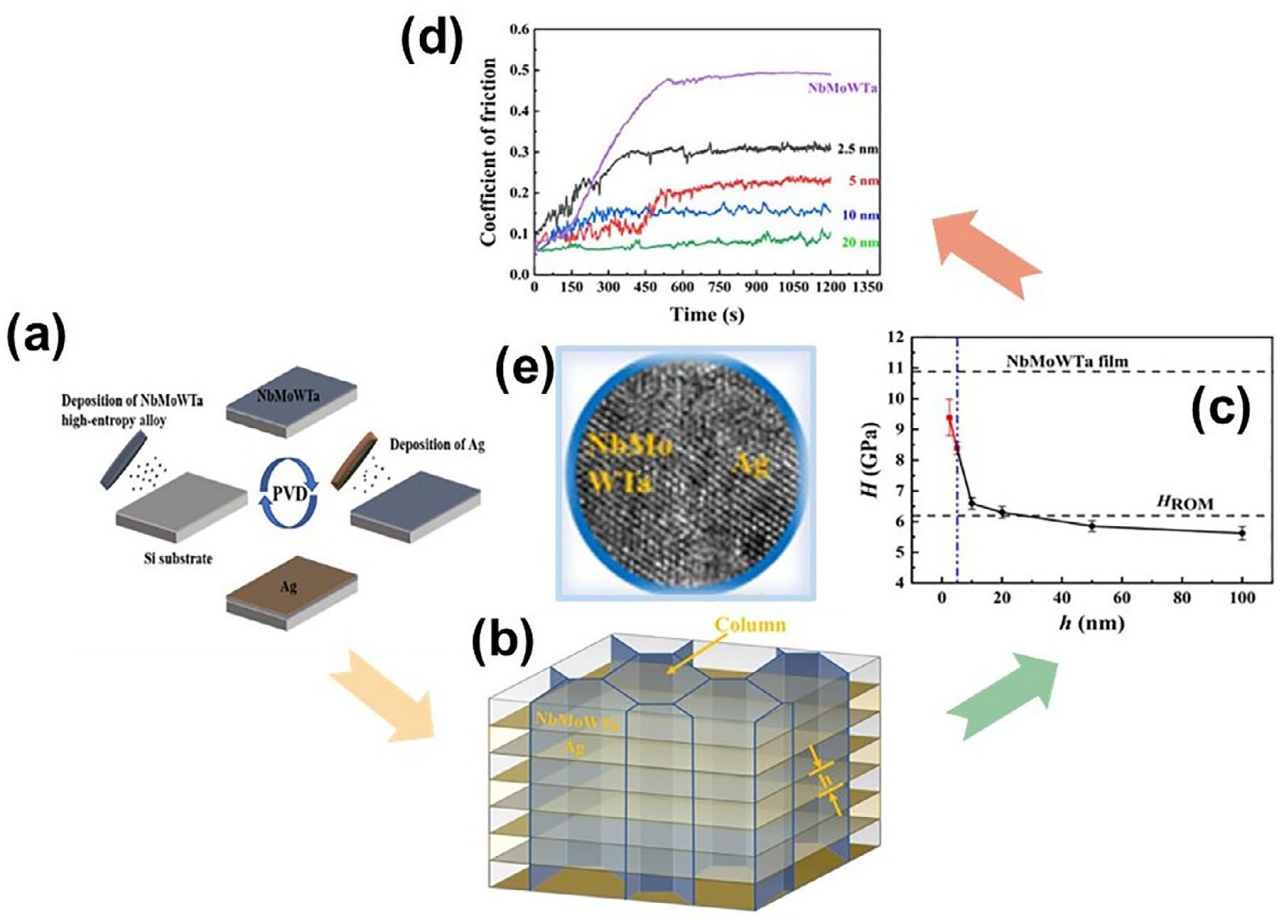
a,b) Schematic diagram and c–e) Hardness, tribological properties, and microstructure of NbMoWTa/Ag multilayer films.^[^ ^202^ ^]^ Copyright 2020, American Chemical Society.

In another advancement, Luo et al.^[^ ^202^ ^]^ developed NbMoWTa/Ag self‐lubricating multilayer films with varied sublayer thicknesses using magnetron sputtering. The addition of Ag prominently improved wear resistance and lubrication by facilitating a transition in the deformation mechanism (**Figure** **20**a,b). As the sublayer thickness decreased, the primary strengthening mechanism shifted from the Hall–Petch effect to a coherent interface strengthening mechanism, enhancing load‐bearing capacity. Furthermore, the formation of a durable lubrication film due to the addition of Ag effectively reduced interfacial shear force, effectively overcoming the inherent brittleness of NbMoWTa films and profoundly improving their tribological performance (Figure 20c–e).

Rational elemental selection serves as a cornerstone in the design of wear‐resistant MPEA‐based films and coatings, enabling precise control over microstructural evolution and the formation of reinforcing and lubricating phases. By leveraging the thermodynamic tendencies of selected elements, it is plausible to tailor phase structures, such as carbides, nitrides, and nanocomposites, significantly enhancing hardness, toughness, and tribological performance. Strategic additions of nitrogen or carbon, for example, not only induce phase transformations from softer matrices to harder crystalline or amorphous structures but also enable in situ formation of ceramic reinforcements and lubricating phases. These effects collectively contribute to reduced friction, improved resistance to plastic deformation, and enhanced durability under aggressive wear conditions.

Braic et al.^[^ ^150^ ^]^ investigated the tribological and wear resistance properties of TiAlCrNbY MPEA films produced by reactive magnetron sputtering, along with their corresponding carbide films with varying carbon content. They found that the friction coefficient of the carbide films was significantly lower than that of the alloy films, and further decreased with increasing carbon content. This reduction in friction was attributed to the formation of a carbon‐rich surface layer that acted as a solid lubricating phase, a phenomenon also observed in (CuSiTiYZr)C_X_ MPEA carbide films.^[^ ^151^ ^]^ Such in situ‐formed lubricating layers are particularly effective in minimizing shear stress and wear during sliding contact.

Ren et al.^[^ ^51^ ^]^ extended this approach to nitride systems by preparing (AlCrMoNiTi)N_X_ and (AlCrMoZrTi)N_X_ films via magnetron sputtering. Their results demonstrated that the nitride films exhibited markedly improved wear resistance compared to their metallic counterparts, primarily due to their enhanced hardness and structural stability. Notably, the (AlCrMoZrTi)N_C_ films outformed (AlCrMoNiTi)N_X_ films, a difference attributed to the larger atomic radius and stronger nitride‐forming ability of Zr. The incorporation of Zr led to improved mechanical properties by refining the microstructure and enhancing phase stability under stress.

**Figure 21:**
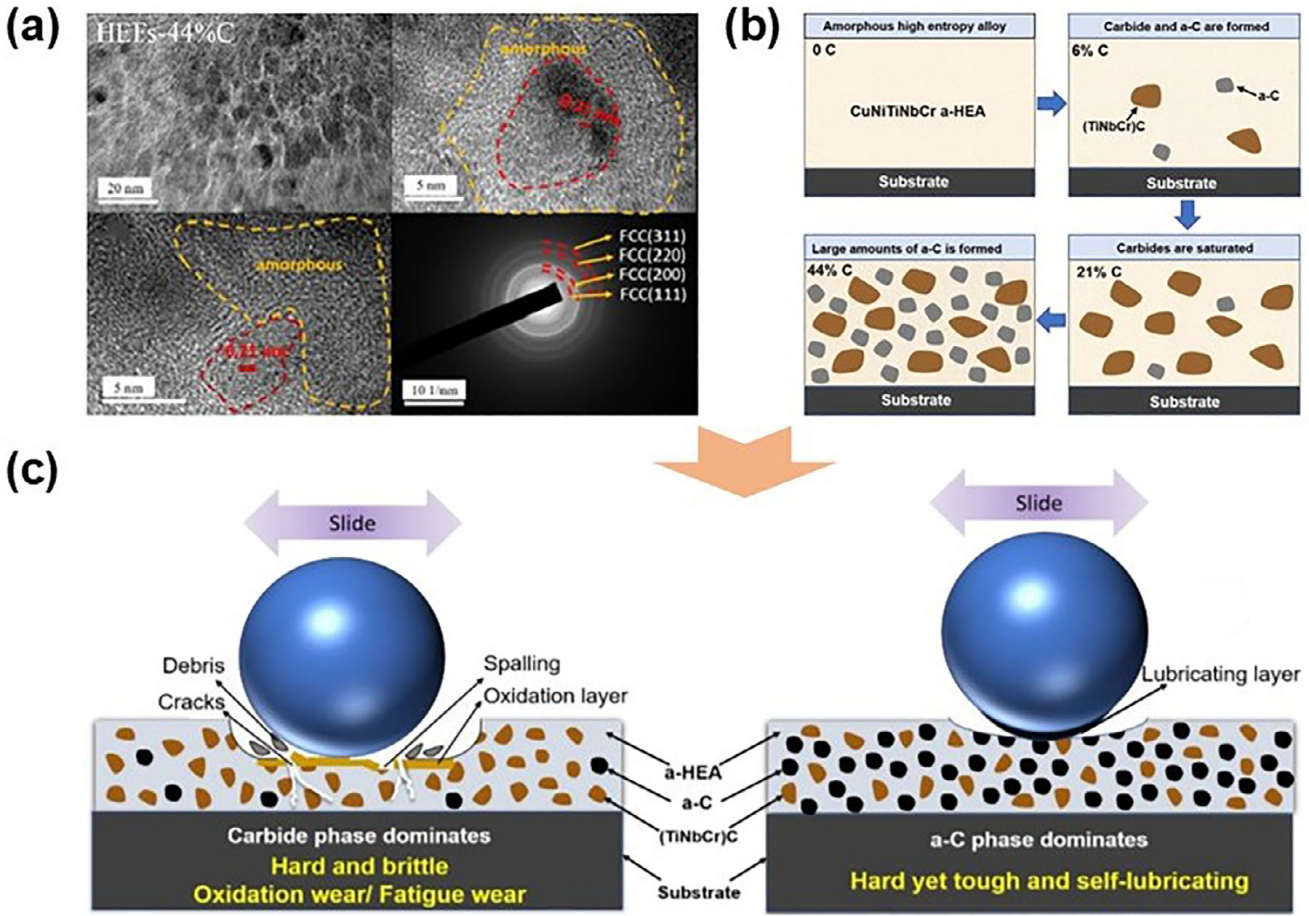
a) TEM images and the corresponding diffraction pattern of the (CuNiTiNbCr)C_X_ (X = 44%) film; Schematic diagram of b) structural evolution and c) wear mechanism of (CuNiTiNbCr)C_X_ films.^[^ ^203^ ^]^ Copyright 2024, Elsevier.

In another study, Li et al.^[^ ^203^ ^]^ prepared (CuNiTiNbCr)C_X_ nanocomposite MPEA films using dual‐target co‐sputtering, systematically varying carbon content to assess its impact on microstructure and tribological performance. With increasing carbon content, carbon atoms preferentially bonded with Ti, Nb, and Cr to form a nanocrystalline (TiNbCr)C ceramic reinforcement phase, while excessed carbon precipitated as amorphous carbon (a‐C). As shown in **Figure** **21**a,b, this induced a structure transitioned from an amorphous phase to a nanocomposite architecture comprising a (CuNiTiNbCr + a‐C) amorphous matrix embedded with (TiNbCr)C nanocrystals. The film with 44.0 at.% carbon exhibited the best balanced of hardness and toughness, with outstanding resistance to fatigue crack propagation and the formation of an interfacial lubricating layer during friction (Figure 21c), thereby significantly improving its wear resistance.

**Figure 22:**
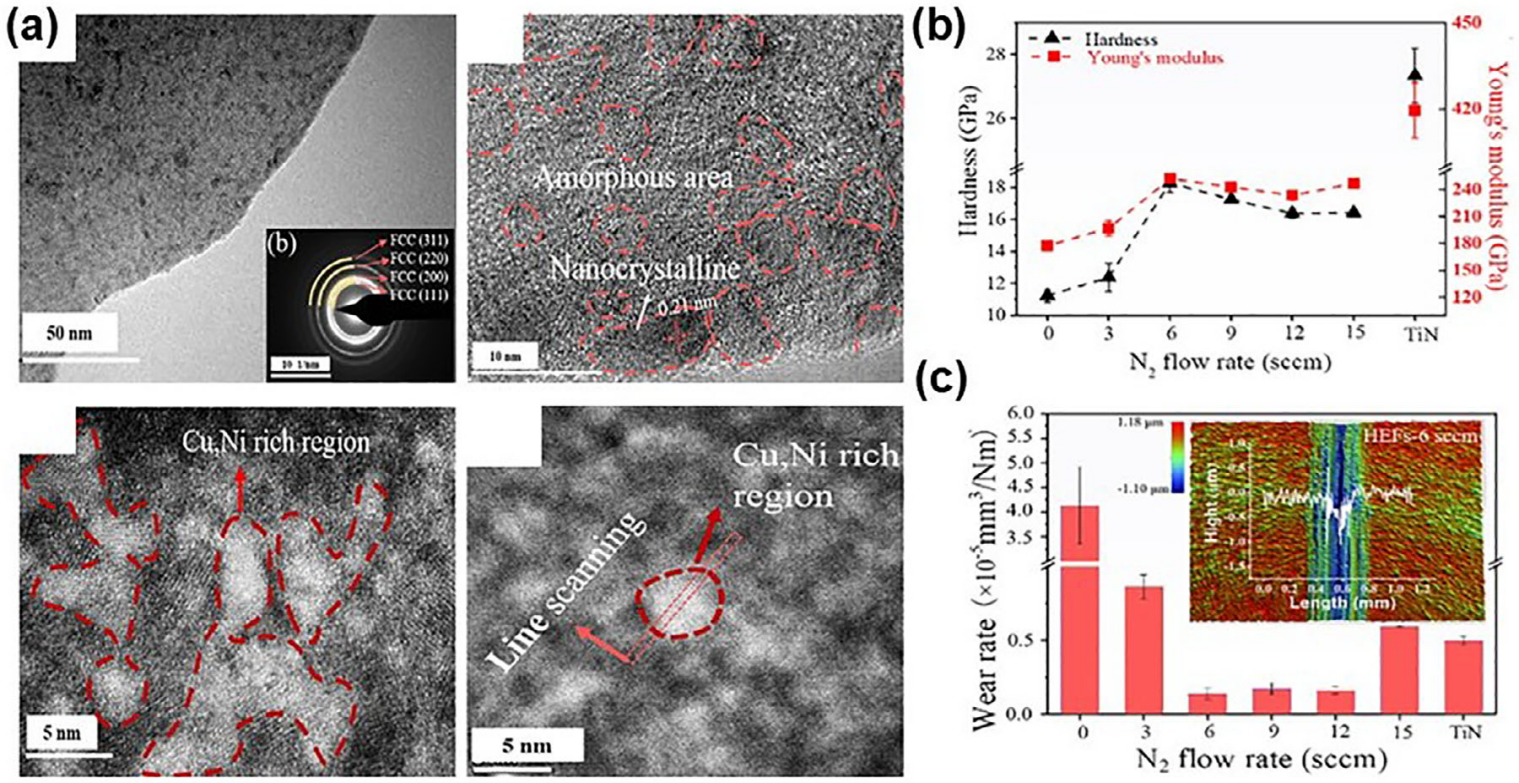
a) TEM images of (CuNiTiNbCr)N films deposited at F_N_ = 6 sccm; b) Evolution hardness and indentation modulus of (CuNiTiNbCr)N films with N_2_ flow rate; c) Variations in wear rate of the (CuNiTiNbCr)N films with N_2_ flow rate.^[^ ^15^ ^]^ Copyright 2024, Elsevier.

Similarly, Sha et al.^[^ ^163^ ^]^ explored the incorporation of nitrogen into CoCrFeNiMn alloy films to develop high‐entropy nitride coatings which prepared by magnetron sputtering. Nitrogen addition triggered a phase transformation toward a harder BCC phase and significantly increased film hardness. Due to the high hardness and oxidation resistance of nitrides, the resultant nitride films showed reduced plowing and oxidative wear. Films with higher nitrogen content displayed smoother and more uniform wear tracks, indicating reduced wear damage. Moreover, their dense nanocrystalline structure effectively resisted chloride‐induced corrosion, endowing them with excellent corrosion and wear resistance.^[^ ^204^ ^]^ Building on this strategy, Li et al.^[^ ^15^ ^]^ designed (CuNiTiNbCr)N_X_ nanocomposite films through element selection guided by thermodynamic principles of phase formation. During deposition, the introduced nitrogen atoms reacted with Ti, Nb, and Cr to form a (TiNbCr)N phase, while Cu and Ni preferentially segregated into the amorphous matrix. As depicted in **Figure** **22**a, this led to a transition from a fully amorphous structure to a nanocomposite structure comprising (TiNbCr)N nanocrystals within a Cu‐ and Ni‐rich amorphous matrix. The resulting (CuNiTiNbCr)N_X_ films exhibited high hardness and toughness, enabling them to accommodate plastic deformation without cracking or localized brittle fracture. This synergistic combination of structural refinement and phase distribution led to a notable enhancement in wear resistance (Figure 22b,c).^[^ ^201^, ^205^ ^]^

Improving the adhesion strength between MPEA film/coating and the substrate is crucial, as stronger interfacial bonding enables the film/coating to withstand higher mechanical loads and endure prolonged sliding contacts, thereby significantly enhancing wear resistance. This is because interfacial adhesion plays a pivotal role in the friction and wear process: when the adhesion is weak, the coating is more susceptible to delamination or premature failure, which compromises its protective function. To address this challenge, Cheng et al.^[^ ^206^ ^]^ introduced a transition layer (Ti, Cr) of appropriate thickness prior to depositing the (AlCrTaTiZr)N film. This transitional layer effectively improved the adhesion between the film and the substrate, allowing the film to endure longer sliding durations under identical loading conditions.

In addition to interfacial engineering, improving the surface quality of MPEA‐based films and coatings is another effective strategy for enhancing wear performance. Rough surfaces with high contact stress and small real contact areas are more likely to reach their load limits during sliding, which increases the risk of surface damage and delamination. To mitigate this, Lo et al.^[^ ^161^ ^]^ investigated the effect of substrate bias voltage on the wear resistance of (AlCrNbSiTiMo)N films. They found that increasing the substrate bias voltage substantially improved wear resistance. This improvement was attributed to the higher kinetic energy of sputtered atoms at elevated bias voltages, inducing a re‐sputtering effect and creating numerous vacancy defects that inhibited grain growth. Concurrently, adsorbed atoms gained additional energy from high‐energy particle flux, enhancing their surface mobility and promoting nucleation at these defect sites. As a result, films deposited under higher bias voltages exhibited smoother, denser, and finer‐grained microstructures, enabling superior wear resistance.

**Figure 23:**
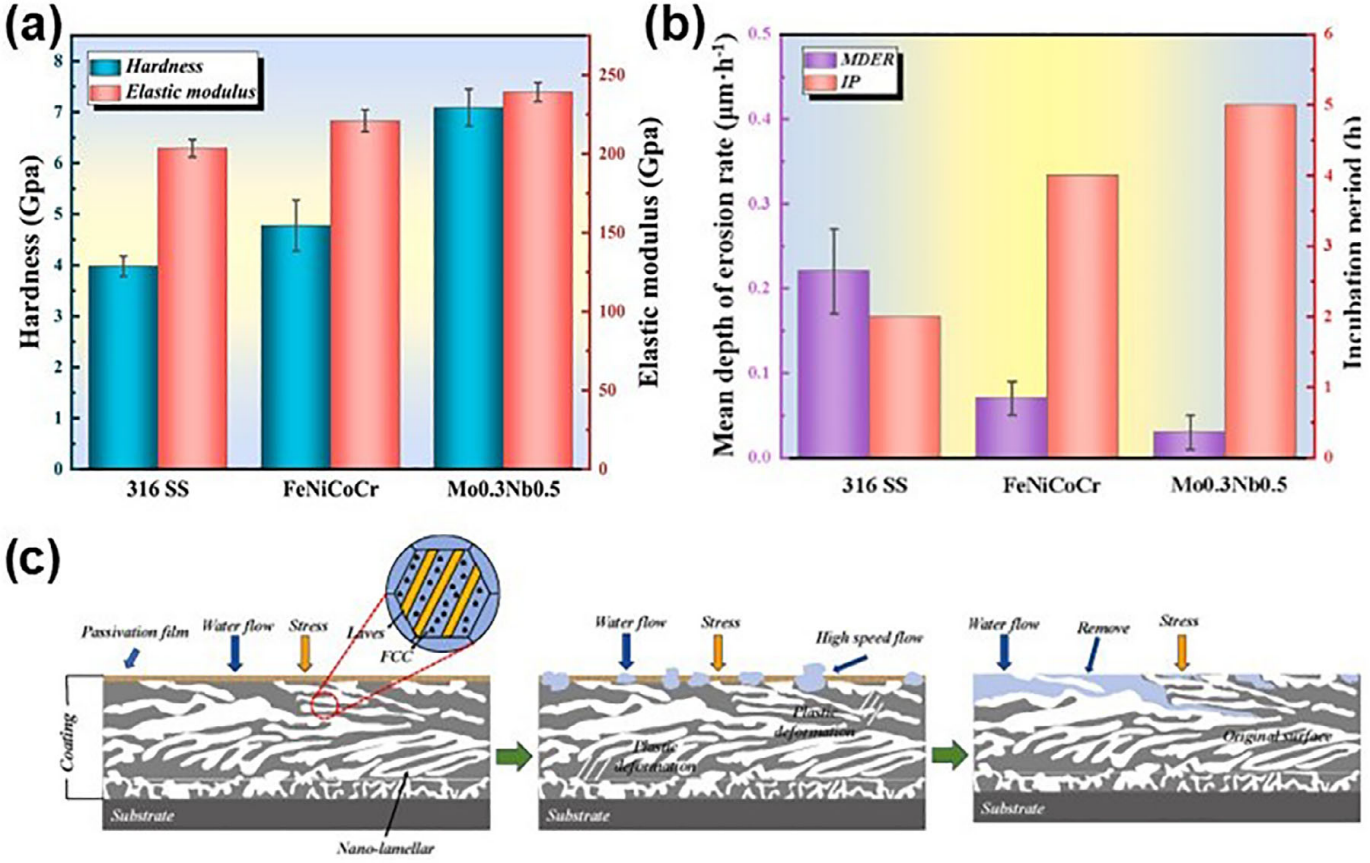
a) Hardness of 316 stainless steel and MPEA coatings; b) Mean depth of erosion rate of 316 stainless steel and MPEA coatings; c) Schematic diagram of cavitation erosion mechanism of FeNiCoCrMo_0.3_Nb_0.5_ coating.^[^ ^211^ ^]^ Copyright 2025, Elsevier.

Beyond wear resistance, recent studies have highlighted the significance of erosion and cavitation resistance in MPEA‐based films and coatings, particularly for hydraulic, marine, and energy‐related applications.^[^ ^207^, ^208^, ^209^, ^210^ ^]^ For example, a novel FeNiCoCrMo_0.3_Nb_0.5_ coatings prepared by laser cladding adopted a hypoeutectic dual‐phase architecture (FCC + Laves), where Mo and Nb jointly promoted lattice distortion and precipitation hardening.^[^ ^211^ ^]^ As depicted in **Figure** **23**a,b, this dual strengthening mechanism raised nanohardness up to 7.09 GPa and simultaneously enhanced cavitation erosion resistance, with the mean depth of erosion rate value 7.3 times lower than that of 316 stainless steel. The Laves phase contributed both structural rigidity and localized erosion resistance, stabilizing the surface under microjet impact (Figure 23c). Similar improvements were observed in CoCrNiTi_X_ coatings fabricated by laser melting deposition, where Ti content strongly influenced performance. The CoCrNiTi_0.15_ coating, with Ti fully dissolved in the FCC matrix, exhibited the optimal solid‐particle erosion resistance in acidic media. This was attributed to Ti‐enhanced yield strength and strain‐hardening capacity through extensive interactions between high‐density dislocations and twins, resulting in a cumulative mass loss only one‐third that of 904L stainless steel.^[^ ^212^ ^]^ In addition, rare‐earth doping has also proved viable and effective. AlCoCrFeMo coatings doped with Scandium (Sc) via flame spraying deposition exhibited refined splat morphologies, reduced porosity (1.1 ± 0.3%), and enhanced oxide formation.^[^ ^213^ ^]^ Notably, the AlCoCrFeMo‐0.3Sc variant achieved a 63% reduction in friction coefficient and a 25% lower erosion rate relative to the undoped coating, owing to the combined effects of grain refinement, solid‐solution strengthening, and the lubricating role of rare‐earth elements.

**Figure 24:**
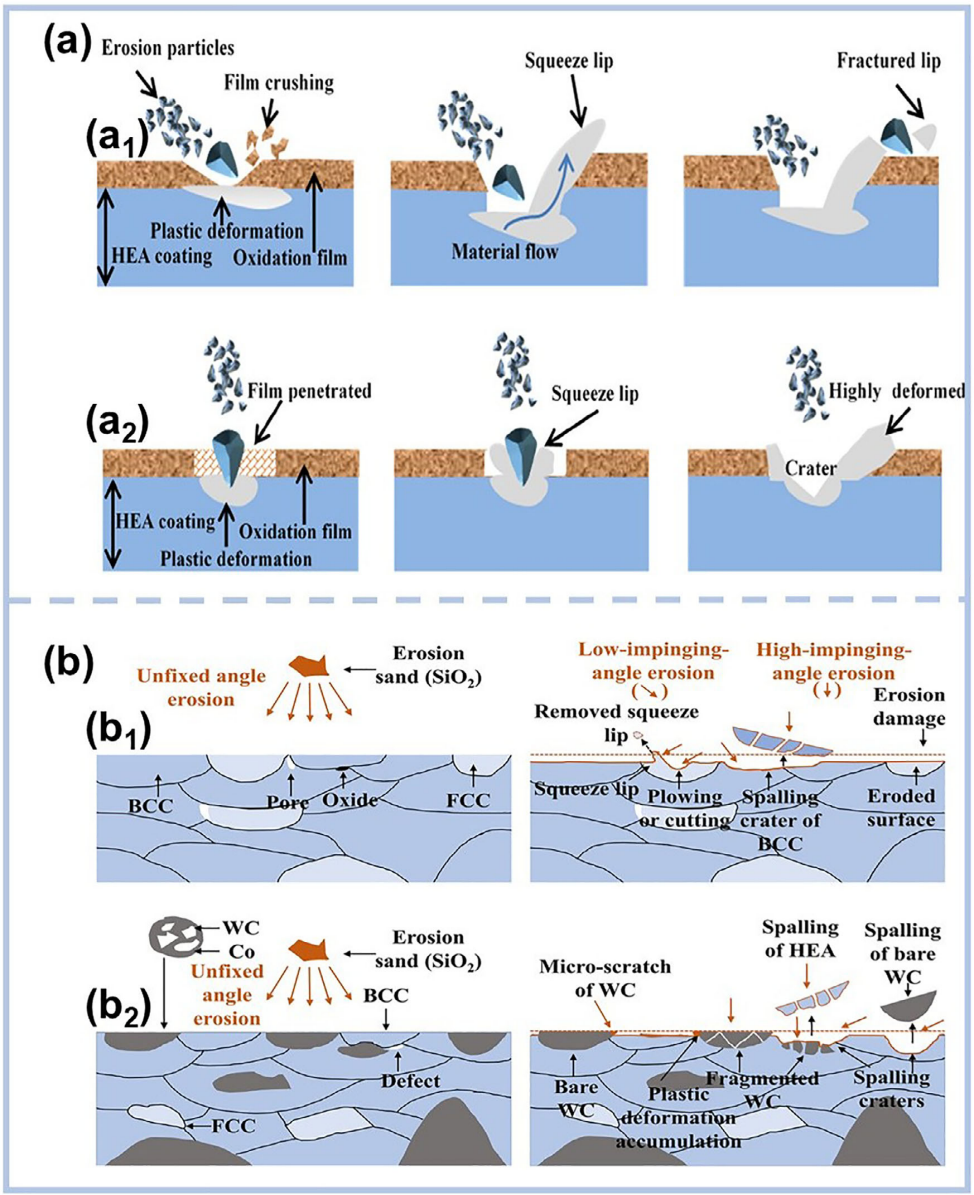
a) Schematic diagram of erosion mechanism for CoCrFeNiSi: a_1_) oblique impact angles; a_2_) vertical impact angles.^[^ ^215^ ^]^ Copyright 2021, Elsevier. b) Schematic diagram of the erosion mechanism of the coatings: b_1_) AlCoCrFeNi coating, b_2_) (AlCoCrFeNi)x/(WC‐10Co)1‐x coating.^[^ ^216^ ^]^ Copyright 2023, Elsevier.

Deposition techniques and structural design also exert a decisive influence on erosion resistance. For instance, CoCrFeNiTiMo coating deposited on a Ti‐6Al‐4 V substrate using double cathode glow discharge formed a duplex BCC + Co_2_Mo_3_ structure with a ≈10 µm deposition layer and a 2 µm diffusion layer.^[^ ^214^ ^]^ This gradient architecture increased hardness, enhanced H/E ratio, and achieved strong metallurgical bonding with the substrate, enabling the coating to resist cavitation erosion in chloride‐rich environments with markedly reduced mass loss. Similarly, laser‐cladded CoCrFeNiSi coatings benefited from rapid solidification (10^4^–10^6^ K s^−1^), which suppressed elemental segregation and stabilized a dense FCC matrix with pronounced lattice distortion.^[^ ^215^ ^]^ The coating retained high hardness at 500–700 °C and surpassed conventional Co‐based alloys in high‐temperature erosion resistance, underscoring the critical role of nonequilibrium solidification. As shown in **Figure** **24**a, erosion rates increased with temperature at different impact angles, with the lowest erosion rates observed at a 90° impact angle. In another approach, high‐velocity oxygen fuel spraying of (AlCoCrFeNi)_x_/(WC‐10Co)_1‐x_ composite coatings introduced hard WC phases that served as crack arresters and load‐bearing reinforcements.^[^ ^216^ ^]^ As illustrated in Figure 24b, these coatings achieved hardness up to 823 HV and halved erosion volume loss compared with single‐phase MPEAs, with performance boosted by the combined action of MPEA matrix dislocation strengthening and WC fatigue resistance.

Despite these advances, several challenges and limitations remain. Conventional CoCrFeNi alloys are prone to elemental segregation within dendritic and interdendritic regions,^[^ ^217^ ^]^ which act as initiation sites for cavitation erosion and can result in worse resistance than 304 stainless steels. This emphasized the necessity of processing routes that suppress segregation and stabilize homogeneous microstructures. Similarly, Al_x_Cu_0.5_FeNiTi coatings prepared by laser surface alloying formed mixed FCC + BCC structures with ultra‐high hardness (≈740 HV) but displayed brittle–ductile mixed erosion mechanisms.^[^ ^217^ ^]^ This trade‐off illustrates that excessive pursuit of hardness without sufficient phase balance can compromise overall durability. Overall, these findings reinforce that wear and erosion resistance in MPEA‐based films and coatings cannot be attributed to a single factor. Instead, it arises from the integrated regulation of alloying elements, microstructural features, processing routes, and interfacial bonding. Future progress requires coordinated design strategies, such as optimizing interfacial bonding through diffusion layers^[^ ^214^ ^]^ or improving densification via substrate bias,^[^ ^216^ ^]^ to sustain wear and erosion resistance under prolonged and cyclic loading.

The wear and erosion resistance of MPEA‐based films and coatings can be further advanced through a multifaceted design strategy that integrates compositional tuning, microstructural refinement, and interface engineering, and significant advances in deposition techniques such as substrate bias control, multilayer architectures, and the introduction of adhesion‐promoting interlayers, strengthening film‐substrate bonding and mitigating delamination and crack propagation under high‐stress sliding conditions. The development of next‐generation MPEA‐based coatings will greatly benefit from the convergence of high‐throughput experimental screening, machine learning‐assisted design, and advanced characterization techniques such as in situ tribological testing and nanoscale mechanical mapping. These tools will accelerate the discovery of novel MPEA systems with adaptive and multifunctional tribological behavior. Furthermore, emerging directions such as gradient‐structured coatings, compositionally complex multilayers, and environmentally responsive surfaces open new possibilities for tailoring wear and erosion resistance in dynamic operating environments. These advances hold direct relevance for critical high‐performance applications where MPEA‐based films and coatings must endure simultaneous wear and erosion, particularly in aerospace engines, marine equipment, energy systems, and chemical processing plants.

### 3.4 Thermal Stability

In addition to outstanding mechanical, corrosive, and tribological performance, MPEA‐based films and coatings are increasingly valued for their remarkable thermal stability, an essential property for applications exposed to extreme temperatures and harsh environments. Their inherent chemical complexity and sluggish diffusion effect contribute to superior phase stability, microstructural integrity, and oxidation resistance under elevated temperatures.^[^ ^218^ ^]^ These attributes not only ensure long‐term performance but also preserve the functional properties of MPEA‐based films and coatings in demanding service conditions, making them attractive candidates for high‐temperature structural, aerospace, and energy‐related applications. In this section, recent advances in enhancing the thermal stability of MPEA films and coatings are discussed, with a focus on composition design, microstructural evolution, and oxidation behavior at elevated temperatures.

**Figure 25:**
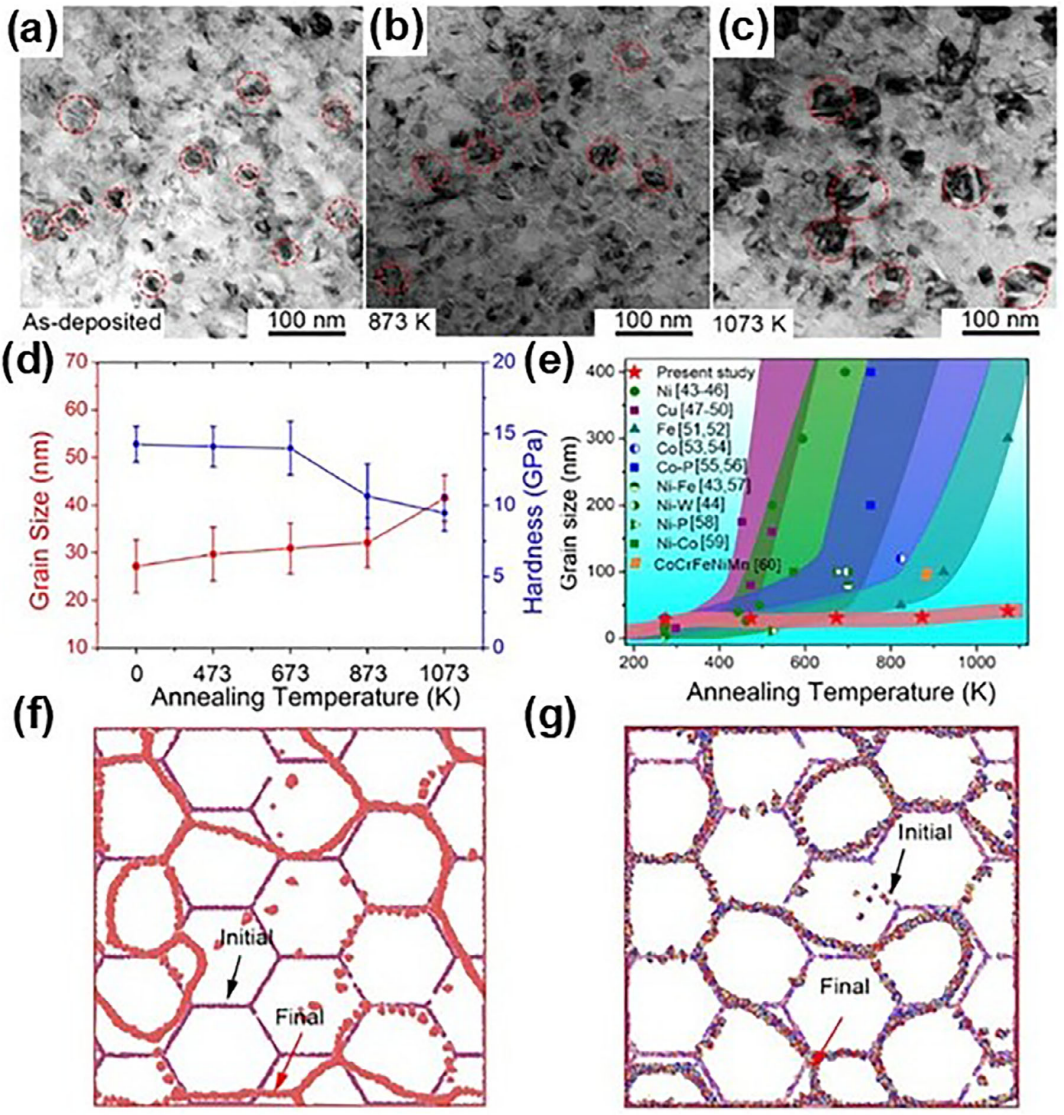
TEM images of a) as‐deposited, b) 873 K‐annealed, and c) 1073 K‐annealed CrCoNi films) d) Variations in grain size and hardness of CrCoNi films with annealing temperatures. e) Grain size vs annealing temperature curves for the CrCoNi and other metallic materials. Molecular dynamics simulations of the thermal stability behavior of f) Ni and g) CrCoNi at 1000 K.^[^ ^174^ ^]^ Copyright 2022, Elsevier.

The exceptional thermal stability of MPEA‐based films and coatings is primarily attributed to high‐entropy and sluggish diffusion, which significantly suppress atomic mobility during annealing even at elevated temperatures. This inherently low diffusion rate hinders elemental redistribution and grain growth, allowing these materials to retain their structural integrity under high temperature exposure or even thermal stress. For instance, Haché et al.^[^ ^27^ ^]^ fabricated nanocrystalline NiFeCo and NiFeCr coatings via electrodeposition and observed excellent thermal stability. The critical temperature for grain growth or phase decomposition was increased by ≈100 °C in NiFeCo and by 200 °C in NiFeCr compared to conventional alloys. Calorimetric studies further revealed that the activation energy for grain growth in NiFeCo was elevated by 0.4–1.1 eV, comparable to that of Co–P owing to the solute drag effect and Zener drag mechanisms. Similarly, Gao et al.^[^ ^174^ ^]^ prepared columnar nanocrystalline CrCoNi films with an average grain size of ≈26 nm using magnetron sputtering, as shown in **Figure** **25**a. Notably, after annealing at 873 K (Figure 25b) and 1073 K (Figure 25c) for 2 h, the films exhibited only slight grain growth, while retaining nanotwins and high‐density stacking faults within the nanocrystalline structure. These features played a crucial role in maintaining exceptional mechanical performance. The as‐deposited films exhibited a high hardness of 14 GPa, which remained at a notable 9 GPa even after annealing at 1073 K, as exhibited in Figure 25d,e, which was over three times that of the coarse‐grained counterparts.^[^ ^219^ ^]^ Molecular dynamics simulations revealed the combination of dense nanocrystalline structures, TBs with low mobility, and the reduced interfacial energy contributed synergistically to the outstanding thermal stability, enabling simultaneous enhancement of strength and thermal resistance, as demonstrated in Figure 25f,g.

Severe lattice distortion in MPEA‐based films and coatings serves as a key mechanism for suppressing grain growth during annealing, thereby enhancing the stability of the crystal structure under high‐temperature conditions. This structural stability is vital for preserving mechanical performance and phase integrity during thermal exposure. Wu et al.^[^ ^149^ ^]^ investigated the thermal behavior of Al_X_CoCrFeNiCu MPEA films fabricated via magnetron sputtering. Both films exhibited uniform elemental distribution in depth and nanometer‐sized grains with pronounced lattice distortion. The Al‐free film with an FCC structure remained stable up to 773 K under the same conditions. The film with Al addition, which adopted a BCC structure, remained structurally stable up to 873 K for 20 min, and its hardness and reduced Young's modulus increased slightly upon annealing.

Tsai et al.^[^ ^159^ ^]^ deposited TiVCrZrHf nitride films via magnetron sputtering, with the microstructure evolving from an amorphous, randomly oriented layer to columnar grains with an FCC structure, which was driven by severe lattice distortion and refined grain size. Notably, no significant microstructural changes were observed even after annealing at 1073 K, indicating robust thermal resistance. In a subsequent study, Tsai et al.^[^ ^220^ ^]^ synthesized (AlMoNbSiTaTiVZr)_50_N_50_ nitride films using reactive magnetron sputtering. The as‐deposited films exhibited an amorphous structure, which remained unchanged after annealing at 850 °C for 30 min. This remarkable thermal stability was ascribed to the combined effects of severe lattice distortion, reducing the diffusion kinetics of elements, and high packing density induced by the presence of multiple principal elements, which limited atomic mobility by reducing available free volume for diffusion. Further evidence of thermal resilience was provided by Sheng et al.,^[^ ^164^ ^]^ who prepared NbTiAlSiN and NbTiAlSiWN nitride films via magnetron sputtering. Both coatings initially exhibited an amorphous structure and maintained phase stability for over 24 h at 700 °C. Crystallization was only observed upon exposure to 1000 °C, demonstrating the films' robust resistance to thermally induced structural degradation.

**Figure 26:**
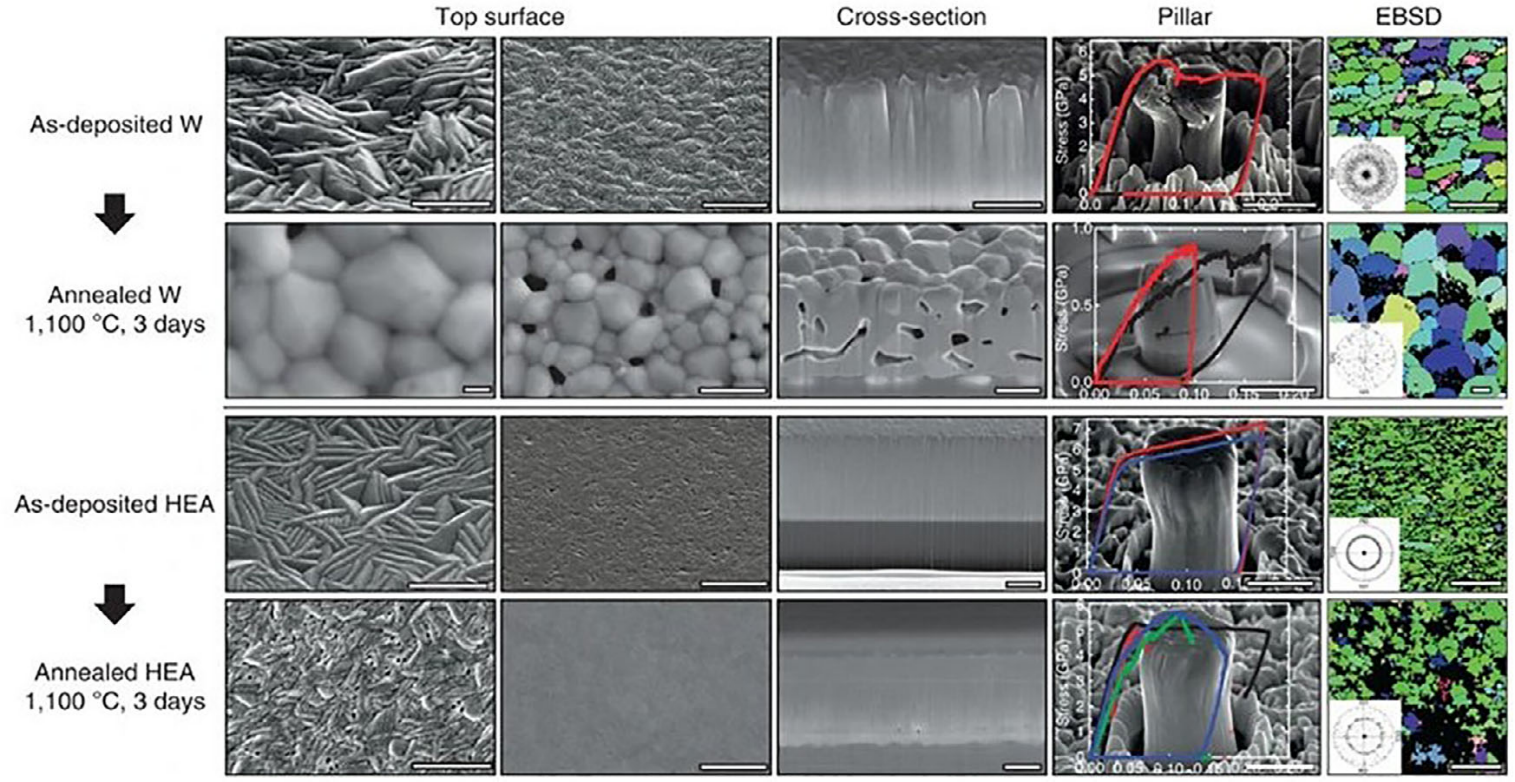
Pre‐ and post‐annealing structures of the W and NbMoTaW MPEA films at 1100 °C for 72 h.^[^ ^221^ ^]^ Copyright 2015, Springer Nature.

Compared to conventional MPEA systems, refractory‐based MPEA films, composed of elements with high melting points and low diffusion rates, exhibit superior resistance to grain coarsening, phase transformation, and structural degradation at elevated temperatures, representing a frontier in materials design for applications where both thermal endurance and mechanical performance are critical. Zou et al.^[^ ^221^ ^]^ demonstrated the exceptional high‐temperature phase stability of NbMoTaW MPEA films fabricated via magnetron sputtering. As illustrated in **Figure** **26**, these films maintained a stable microstructure even after prolonged exposure at 1100 °C for 72 h. In stark contrast to pure W films, the annealed NbMoTaW film preserved a uniform needle‐like surface morphology without significant grain growth, and the entire cross‐section remained dense without apparent porosity. Mechanically, the NbMoTaW film pillars retained superior strength and ductility before and after annealing, significantly outperforming W film counterparts (Figure 26). Notably, the yield strength of the annealed NbMoTaW film pillar remained at ≈5 GPa, nearly identical to the as‐deposited state. This exceptional thermal‐mechanical stability was attributed to the formation of a nanocrystalline structure characterized by highly disordered internal grains. The reduction in GB energy compared to pure metals dramatically decreased the driving force for GB migration, thereby suppressing grain coarsening during high‐temperature exposure.

**Figure 27:**
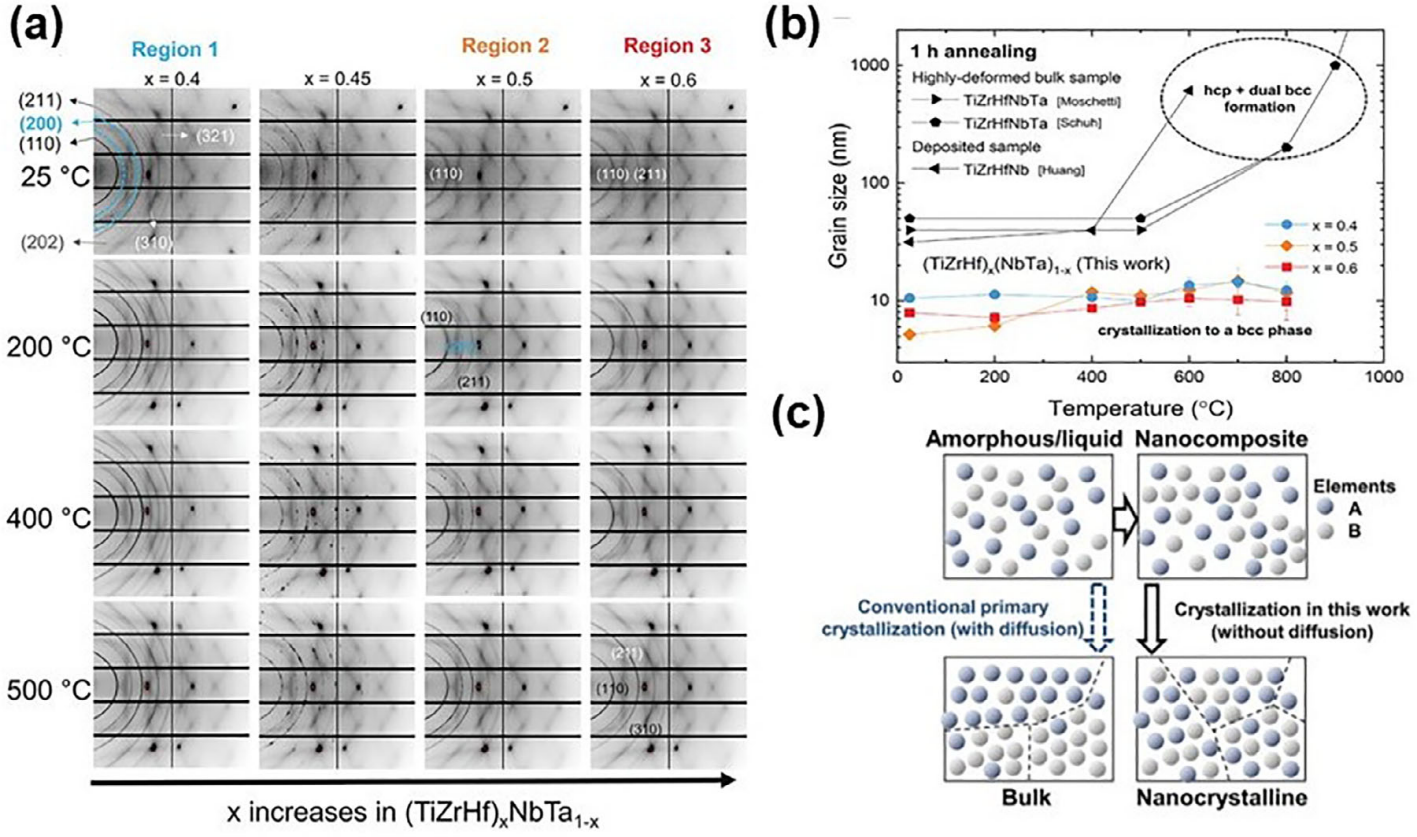
a) Synchrotron XRD results of the as‐deposited and annealed (TiZrHf)_X_(NbTa)_1‐X_ films up to 500 °C; b) Comparison of grain size stability between this work and other studies; c) Schematic illustration of primary crystallization mechanisms.^[^ ^11^ ^]^ Copyright 2024, Wiley‐VCH.

Expanding on the understanding of phase evolution and grain growth behavior, Cheng et al.^[^ ^11^ ^]^ employed a high‐throughput magnetron co‐sputtering approach coupled with in situ heating to systematically investigate the thermal response of (TiZrHf)_X_(NbTa)_1‐X_ MPEA films. As shown in **Figure** **27**a, the study utilized synchrotron X‐ray diffraction and detailed microstructural characterization to track phase transformations and grain size evolution across a temperature gradient. The thermal stability varied with composition: films rich in NbTa favored the formation of thermally stable dual‐BCC phases upon annealing, while TiZrHf‐rich films maintained a single‐phase nanocomposite structure that inhibited crystallization and grain growth. As depicted in Figure 27b, grain size remained below 15 nm, demonstrating excellent resistance to thermal coarsening even when compared to earlier studies where dual‐BCC or HCP phase formation led to considerable grain growth. Notably, the slight grain growth observed in some films was linked to a crystallization process. However, unlike conventional crystallization mechanisms,^[^ ^222^ ^]^ which were typically diffusion‐controlled and involved pronounced chemical segregation, this transformation occurred with negligible elemental segregation (Figure 27c). This behavior underscores a fundamentally different kinetic pathway for phase evolution in these high‐entropy systems, enabling them to retain their fine‐grained, thermally stable structures under extreme conditions.

**Figure 28:**
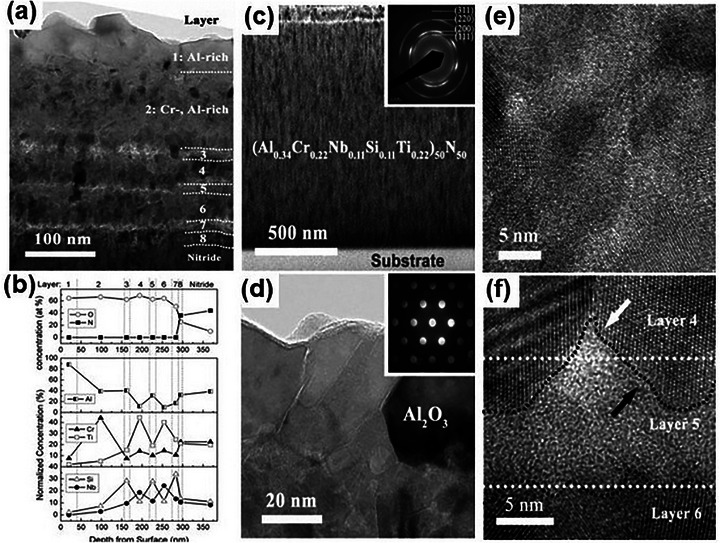
a) TEM images of the (Al_0.34_Cr_0.22_Nb_0.11_Si_0.11_Ti_0.22_)_50_N_50_ nitride films after annealing at 900 °C for 50 h; b) EDX results of the oxide layers; c) TEM image and diffraction pattern of unoxidized nitride film underneath the oxide layer; d) TEM image and nanobeam electron diffraction pattern of α‐Al_2_O_3_ oxide particles at surface; e,f) HRTEM images of layer 2 and layers 4 to 6.^[^ ^225^ ^]^ Copyright 2013, IOPScience.

Enhancing the high‐temperature oxidation resistance of MPEA‐based films and coatings is essential for their application in extreme environments, such as aerospace and thermal barrier systems. Strategic elemental additions, particularly Al, Cr, Si, Ti, and W, have been effective in tailoring oxide scale formation and film density, thereby significantly improving thermal durability and oxidation resistance. Tsai et al.^[^ ^223^ ^]^ prepared (AlCrMoTiTa)Si_X_‐N nitride films using reactive magnetron sputtering and systematically investigated the effect of silicon content on oxidation behavior. Silicon prominently enhanced the oxidation resistance of the (AlCrMoTiTa)N films by promoting the formation of a bilayer oxide structure on the surface of the film, consisting of an outer amorphous Al_2_O_3_ layer and an inner amorphous SiO_2_ layer, which effectively blocked further oxygen diffusion. Feng et al.^[^ ^224^ ^]^ explored the oxidation resistance of TaNbTiW MPEA films and found that higher Ti and W content substantially improved thermal oxidation resistance after annealing at 900 °C. This enhancement stemmed from the higher mixing enthalpy of W─O bonds relative to Ta─O and Nb─O, along with the ability of Ti and W to promote a denser microstructure that impeded oxygen ingress. Shen et al.^[^ ^225^ ^]^ further demonstrated exceptional oxidation resistance in (Al_0.34_Cr_0.22_Nb_0.11_Si_0.11_Ti_0.22_)_50_N_50_ nitride films, where a complex eight‐layer oxidation structure developed after annealing at 900 °C for 50 h, as shown in **Figure** **28**a–c. This structure included a top Al_2_O_3_ layer (Figure 28d) and multiple dense amorphous sublayers arranged in a networked configuration (Figure 28e,f), collectively acting as a highly effective barrier to oxygen diffusion.

The exceptional thermal stability and high‐temperature oxidation resistance of MPEA‐based films and coatings stands as one of their most distinctive and valuable attributes, enabling their use in environments where conventional alloys rapidly degrade.^[^ ^226^, ^227^, ^228^ ^]^ The synergistic effects of sluggish diffusion, severe lattice distortion, and stable phase structures contribute to the remarkable resistance to grain growth, phase decomposition, and structural coarsening, even under prolonged high‐temperature exposure. These attributes, further enhanced by tailored microstructures and strategic elemental incorporation, position MPEA‐based films as front‐runners for applications in extreme environments. The development of next‐generation MPEA‐based coatings will increasingly rely on a deep mechanistic understanding of thermally activated processes at the nanoscale and uncover the complex interplay between composition, microstructure, and thermal response. By integrating emerging computational and experimental approaches, future research can accelerate the discovery of ultra‐stable MPEA‐based coatings with adaptive microstructures.

## 4 Applications and Perspectives

### 4.1 Applications

**Figure 29:**
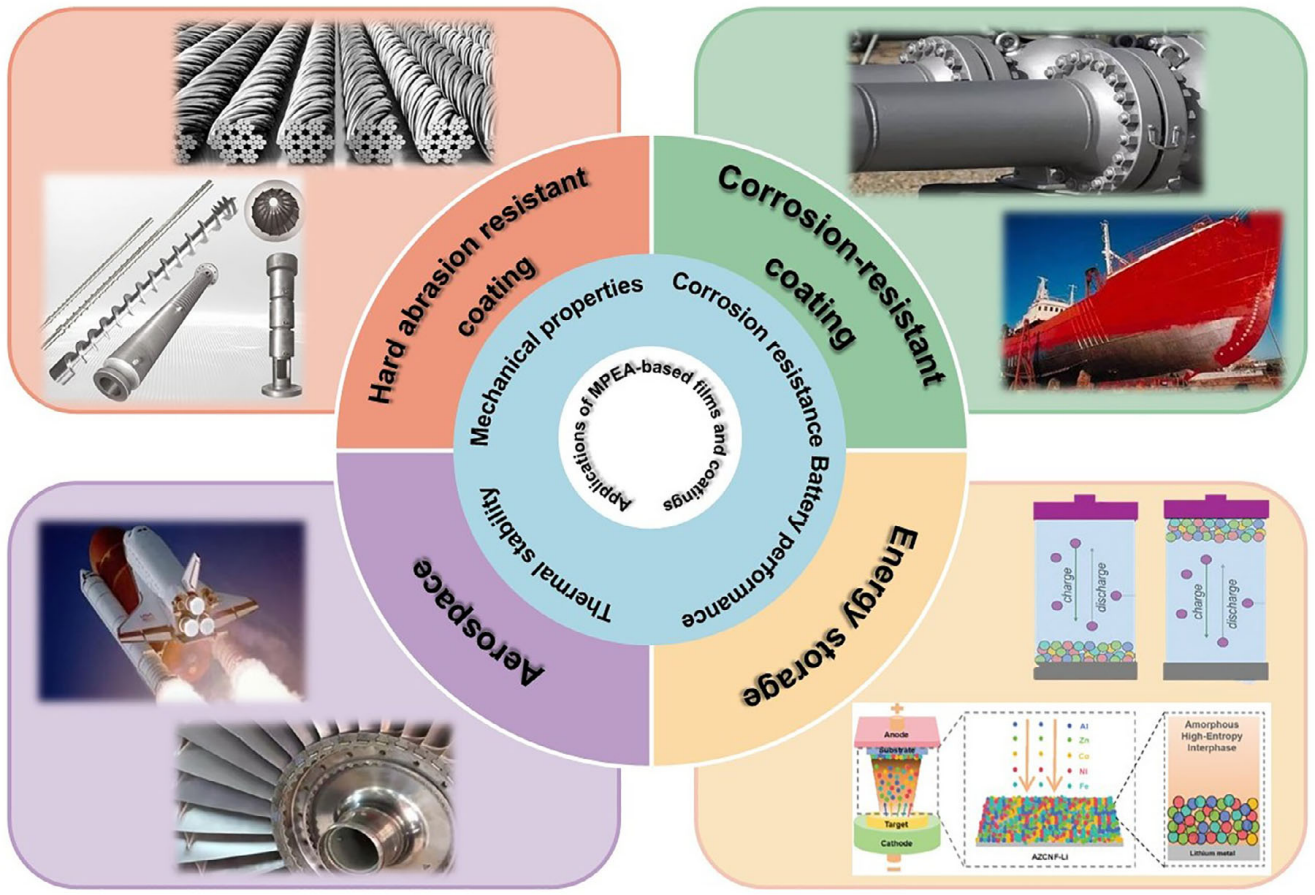
The perspective applications of MPEA‐based films and coatings.

The development of MPEA‐based films and coatings marks a significant leap in materials science, offering a compelling suite of properties that traditional alloy systems struggle to match. With their exceptional hardness, high elastic modulus, remarkable thermal and corrosion stability, and intrinsic compositional tunability, MPEA‐based films and coatings are rapidly emerging as game‐changers across multiple high‐performance applications (**Figure** **29**). Their multifunctionality positions them at the forefront of next‐generation coatings, tailored for increasingly demanding service environments.

One of the most immediate and impactful applications of MPEA‐based films and coatings lies in cutting and mining tools, where their superior hardness and wear resistance translate into longer tool life and enhanced machining efficiency.^[^ ^162^ ^]^ In environments plagued by aggressive chemicals or moisture, MPEA‐based films enriched with corrosion‐resistant elements like chromium form protective barriers that outperform conventional coatings, maintaining structural integrity in marine, chemical, and industrial settings.^[^ ^229^ ^]^

For high‐temperature applications such as aircraft engine components and turbine blades in the energy sector, MPEA‐based coatings demonstrate outstanding thermal stability and oxidation resistance, offering durable protection where traditional materials often degrade.^[^ ^230^ ^]^ This positions them as ideal candidates for thermal barrier and oxidation‐resistant coatings under extreme operating conditions.

The growing need for energy‐efficient technologies also highlights the relevance of MPEA materials. Their stable solid‐state phases, coupled with adaptable electronic structures, make them highly promising in rechargeable batteries, catalysis, energy storage systems, and sustainable technologies, where enhanced electrochemical performance and cycle stability are crucial.^[^ ^231^, ^232^ ^]^ Unlike traditional doped systems, MPEAs offer a more robust platform for sustainable energy applications. In the realm of microelectronics, the sluggish diffusion and lattice distortion effects characteristic of MPEAs slow atom migration. This makes them ideal as ultrathin diffusion barrier layers in nanoscale devices, even at thicknesses of just a few tens of nanometers.^[^ ^159^, ^233^ ^]^ Finally, by tailoring elemental compositions to eliminate toxicity, MPEA‐based coatings, such as alloy carbides and nitrides, can be designed for biomedical applications, offering hard, wear‐resistant, and biocompatible surfaces for implants and medical devices.^[^ ^16^, ^234^ ^]^

### 4.2 Perspectives

Since the concept of MPEAs was introduced in 2004, the field has seen an explosion of innovation. By stepping beyond the constraints of traditional alloy design, MPEAs introduce a vast compositional landscape, enabling millions of unique combinations. This unprecedented tunability, combined with their potential to deliver superior mechanical and functional properties, makes MPEAs a fertile ground for materials discovery. Yet, while the promise is evident, the translation of MPEA‐based films and coatings into widespread industrial use remains an ongoing challenge, demanding a more integrated, forward‐looking research agenda.

#### 4.2.1 Composition Optimization via Computational Design

Unlocking the full potential of MPEA coatings requires the integration of high‐throughput first‐principles calculations, molecular dynamics simulations, and machine learning. These tools enable accelerated screening of candidate compositions and predictive modeling of composition–microstructure–property relationships, advancing beyond empirical trial‐and‐error approaches. Particular emphasis should be directed toward non‐equiatomic compositions, as controlled deviations from equiatomic ratios provide powerful levers for tuning phase stability, tailoring microstructures, and optimizing performance across mechanical, thermal, and corrosion/erosion domains.

#### 4.2.2 Phase Engineering through Tailored Deposition

Tuning the element types and proportions in magnetron sputtering targets or electrodeposition solutions allows for the creation of diverse architectures, ranging from single‐phase solid solutions to amorphous‐nanocrystalline hybrids and complex multiphase systems. Leveraging the high‐entropy effect, sluggish diffusion effect, and synergistic strengthening mechanisms offers a pathway to tailoring performance at the atomic scale.

#### 4.2.3 Enhancing Structural Integrity and Deposition Quality

Adjusting substrate bias can enhance density and induce beneficial residual stresses, while multi‐source co‐sputtering or reactive sputtering techniques can promote elemental homogeneity and structural integrity. In electrodeposition, fine‐tuning current density, potential, and pulse parameters can significantly improve crystallinity, reduce porosity, and ensure uniform co‐deposition of constituent elements. Additives such as complexing agents and surfactants further stabilize solutions and enhance deposition efficiency, ensuring compositional control for uniform, smooth, and defect‐free coatings.

#### 4.2.4 Hierarchical and Heterogeneous Nanostructure Engineering

Next‐generation MPEA‐based coatings will require us to judiciously engineer nanostructural heterogeneities across multiple length scales to meet the strength‐ductility synergy needed in extreme applications.^[^ ^12^, ^235^, ^236^ ^]^ Techniques such as high‐power pulsed sputtering or thermal treatment can be used to develop nanocrystalline or nanotwinned architectures. Moreover, exploiting short‐range order and compositional fluctuations at the atomic level can enable the construction of heterogeneous nanostructures with controlled strain gradients and interface distributions, prominently enhancing both mechanical robustness and work‐hardening capacity.

#### 4.2.5 Real‐World Durability Exploration

Industrial adoption of MPEA‐based coatings demands rigorous validation of reliability and durability. Their wear and corrosion resistance under complex service conditions, such as temperature cycling, salt spray, and mechanical fatigue—need to be thoroughly characterized. Applications in harsh environments like marine platforms, aerospace engines, and chemical reactors demand coatings that perform consistently under simultaneous thermal, mechanical, and chemical stresses.

## 5 Conclusion

MPEA‐based films and coatings have emerged as a transformative class of materials, offering a unique combination of ultrahigh hardness and strength, excellent plasticity, outstanding wear and erosion resistance, exceptional corrosion tolerance, and thermal stability, surpassing the capabilities of many conventional alloy systems. Their unparalleled performance is ingrained in the synergy of compositional complexity and structural tunability—made possible through advanced deposition techniques such as magnetron sputtering and electrodeposition, alongside post‐deposition treatments that refine phase structure, promote nanocrystallinity, and introduce beneficial heterogeneities.

Several critical challenges remain at the forefront of research. These include the precise control of composition‐process‐property relationships, a deeper understanding of nucleation mechanisms during deposition, and the suppression of brittle intermetallic phase that can undermine mechanical integrity. Bridging these gaps will require a multifaceted strategy—integrating computational modeling, machine learning‐guided design, and in situ experimental validation—to accelerate the rational discovery and optimization of new MPEA compositions with unprecedented properties and functions. Moreover, future research must broaden the practical applicability of MPEA films and coatings in extreme operating environments, such as high‐temperature aerospace components, radiation‐shielding layers for nuclear systems, and biocompatible surfaces for next‐generation medical implants. Both equiatomic and non‐equiatomic MPEA compositions should be systematically explored to satisfy diverse performance requirements of engineering applications, balancing mechanical performance with wear/erosion resistance, corrosion tolerance, and thermal stability under service conditions. Key to this effort will be the design of coatings featuring hierarchical microstructures, multilayered and gradient architectures, and engineered atomicscale and nanostructural heterogeneities, unlocking unparalleled combinations of mechanical and functional properties that were previously out of reach. Equally important is the development of scalable and cost‐effective manufacturing techniques is also essential to translate laboratory‐scale breakthroughs into real‐world technologies. The journey is still unfolding, and the path ahead is rich with opportunity and paved with promise, positioning MPEA films and coatings as a cornerstone of next‐generation materials innovation.

## Conflict of Interest

The authors declare no conflict of interest.
